# Identification of DNA-Binding Proteins Using Support Vector Machine with Sequence Information

**DOI:** 10.1155/2013/524502

**Published:** 2013-09-16

**Authors:** Xin Ma, Jiansheng Wu, Xiaoyun Xue

**Affiliations:** ^1^Golden Audit College, Nanjing Audit University, Nanjing 210029, China; ^2^School of Geography and Biological Information, Nanjing University of Posts and Telecommunications, Nanjing 210046, China; ^3^Graduate School of Chinese Academy of Agricultural Sciences, Beijing 100081, China

## Abstract

DNA-binding proteins are fundamentally important in understanding cellular processes. Thus, the identification of DNA-binding proteins has the particularly important practical application in various fields, such as drug design. We have proposed a novel approach method for predicting DNA-binding proteins using only sequence information. The prediction model developed in this study is constructed by support vector machine-sequential minimal optimization (SVM-SMO) algorithm in conjunction with a hybrid feature. The hybrid feature is incorporating evolutionary information feature, physicochemical property feature, and two novel attributes. These two attributes use DNA-binding residues and nonbinding residues in a query protein to obtain DNA-binding propensity and nonbinding propensity. The results demonstrate that our SVM-SMO model achieves 0.67 Matthew's correlation coefficient (MCC) and 89.6% overall accuracy with 88.4% sensitivity and 90.8% specificity, respectively. Performance comparisons on various features indicate that two novel attributes contribute to the performance improvement. In addition, our SVM-SMO model achieves the best performance than state-of-the-art methods on independent test dataset.

## 1. Introduction 

DNA-protein interaction has diverse functions in the cell, and it plays an important role in a variety of biological processes, such as gene regulation, DNA replication, and repair. Identification of DNA-binding proteins is the theoretical basis on many commonly used medicinal techniques. For instance, it is considered as selecting activators and inhibitors in rational drug design [1–3]. It also plays an essential role in discovering potential therapeutics for genetic diseases and proteome function annotation. Therefore, recognition of DNA-binding proteins becomes one of the most important questions in the annotation of protein functions.

In recent years, DNA-binding proteins can be annotated by several experimental techniques such as filter binding assays, X-ray crystallography, and NMR. However, experimental approaches to identify DNA-binding proteins remain time-consuming and expensive. Hence, the computational prediction of DNA-binding proteins is important. Most studies on computational prediction of DNA-binding proteins were based on structures of a query proteins [4–9]. But the problem of consuming time and money, arisen by procuring structure of protein, exist still yet. Therefore, it is important to develop computational methods for identifying DNA-binding proteins directly from amino acid sequence instead of structure information.

Machine learning technique is an effective tool which is widely used to distinguish DNA-binding proteins from nonbinding ones. Cai and Lin developed support vector machine (SVM) and the pseudoamino acid composition, a collection of nonlinear features extractable from protein sequence, to construct DNA-binding proteins prediction [10]. Yu et al. proposed the binary classifications for rRNA-, RNA-, and DNA-binding proteins using SVM and sequence features associated physicochemical properties [11]. A web-server DNAbinder (http://www.imtech.res.in/raghava/dnabinder/) has been developed for identifying DNA-binding proteins and domains from query amino acid sequences. It was constructed by SVM using amino acid composition and PSSM profiles [12]. Shao et al. constructed two classifiers to differentiate DNA/RNA-binding proteins from nonnucleic-acid-binding proteins by using SVM and a conjoint triad feature which extract information directly from amino acids sequence of protein [13]. Patel et al. used an artificial neural network to identify DNA-binding proteins using a set of 62 sequence features [14]. Kumar et al. reported a random forest method, DNA-Prot, to identify DNA-binding proteins from protein sequence [15]. Lin et al. proposed a new predictor, called iDNA-Prot, for predicting uncharacterized proteins as DNA-binding proteins or non-DNA-binding proteins based on their amino acid sequences information alone [16].

In this study, we attempt to predict DNA-binding proteins directly from amino acid sequences. We propose a novel method for predicting DNA-binding proteins using a support vector machine-sequential minimal optimization (SVM-SMO) algorithm in conjunction with a hybrid feature. The hybrid feature is incorporating evolutionary information feature, physicochemical feature, and two novel attributes which represented DNA-binding propensity and nonbinding propensity. Those novel attributes were constructed by DNA-binding residues and nonbinding residues predicted by our previous work DNABR [17], respectively. Our model achieves 0.67 Matthew's correlation coefficient (MCC) and 89.6% overall accuracy with 88.4% sensitivity and 90.8% specificity, respectively by 5-fold cross-validation. In addition, the results demonstrate that the two novel attributes we propose in the research are discriminative to distinguish between DNA-binding proteins from nonbinding proteins. 

## 2. Materials and Methods

### 2.1. Data

We collected DNA-binding proteins and nonbinding proteins from release “2013_02” of UniProtKB/Swiss-Prot database (http://www.uniprot.org/) [18]. To make sure of the reliability of data, we only selected manually annotated and reviewed proteins.

“DNA binding” was used as a keyword to search the UniProtKB/Swiss-Prot database. Then 29866 DNA-binding proteins were retrieved and designated as rough “Positive” dataset.

A “Contrast” dataset was obtained by the similar procedure which was proposed by Cai and Lin [10]. 158121 proteins in “Contrast” dataset were retrieved from UniProtKB/Swiss-Prot database by searching with a list of keywords which possibly imply RNA/DNA-binding functionality using the “or” logic.

Then the proteins in “contrast” dataset were removed from UniProtKB/Swiss-Prot database, and 158121 proteins were obtained to form rough “Negative” dataset.

As indicated by previous research [13, 19], the protein sequences with the length range from 50 to 6000 amino acids are retained. Proteins including irregular amino acid characters such as “*x*” and “*z*” were removed. Moreover, the redundancy among the sequences in “positive” and “negative” datasets was removed by using BLAST package available from NCBI with a threshold of 40% identification. The longest amino acid sequence within each cluster was retained for reaching nonredundant dataset. Finally, 6653 and 60548 proteins were produced in nonredundant “Positive” dataset and “Negative” dataset, respectively. To deal with the imbalance problem between positive data and negative data, we created a “Negative subset” dataset by randomly selecting from “Negative” dataset which has the equal size to the “Positive” dataset. Therefore 13306 proteins contained in “Positive” dataset and “Negative subset” dataset consisted of the main dataset.

 To evaluate the performance of our method against previous works [15, 16], an independent test dataset was used. 1332 DNA-binding proteins from “Positive” dataset and 1332 nonbinding proteins from “Negative subset” dataset were randomly selected to build independent test dataset. We made sure that the proteins in test dataset were not used in previous works [15, 16]. Those remaining proteins in “Positive” dataset and “Negative subset” dataset were designated as the training dataset. Therefore, the training dataset (TrD_10642) obtained 10642 proteins and test dataset (TeD_2664) obtained 2664 proteins (more details can be seen from Table 1 and see Supplementary Material available online at http://dx.doi.org/10.1155/2013/524502).

### 2.2. Feature Vector

#### 2.2.1. Binding Propensity and Nonbinding Propensity (BP and NBP)

It is well known that DNA-binding residues should exist in DNA-binding protein and tend to appear on the surface of DNA-binding proteins. DNA-binding proteins have much more binding residues than nonbinding proteins and tend to gather together spatially. Therefore, these two characters of DNA-binding residues would be applied to identify binding proteins. We already had built a DNA-binding residue prediction model DNABR [17] (http://www.cbi.seu.edu.cn/DNABR/). The performance comparisons with other approaches showed that DNABR has an excellent prediction performance for detecting binding residues in putative DNA-binding protein. Consequently, we proposed binding propensity measures and nonbinding propensity measures which were made based on the prediction results of DNA-binding residues and nonbinding residues, respectively.

 According to two characters of DNA-binding residues mentioned above, two binding propensity measures were defined for as follows:

(1) $$\begin{array}{c} \text{BP}\left(1\right)=\frac{\sum_{i=1}^{n}\text{RI}\left(i\right)}{10N}, \end{array}$$

where *N* is the number of amino acids in this protein, *n* is the number of DNA-binding residues, and RI(*i*) is the predicted reliability index of DNA-binding residue *i* obtained from DNABR. The reliability index is a positive integer range from 0 to 10:

(2) $$\begin{array}{c} \text{BP}\left(2\right)=\frac{\sum_{i=1}^{N-1}2^{-i+1}\sum_{k=1}^{n(i)}\bar{\text{RI}}\left(k\right)}{10\left(N-1\right)}, \end{array}$$

where *N* is the number of amino acids in this protein, *n*(*i*) is the number of two DNA-binding residues with the distance *i*, and $\bar{\text{RI}}\left(k\right)$ is the mean of reliability index for DNA-binding residue *k* and binding residue *k* + *i*.

For a query protein, BP(1) and BP(2) describe the information of the appearance and correlation of DNA-binding residues in the amino acid sequence, respectively. Due to the usage of predicted DNA-binding residue in this paper, reliability index is applied in BP(1) and BP(2) formula. BP(1) represents the frequency of DNA-binding residues. BP(2) represents the relevance of the two DNA-binding residues with different gaps from 1 to *N* − 1 amino acids. The BP(2) formula takes into account the fact that the correlation value between two DNA-binding residues is smaller when the distance *k* is larger. 

Meanwhile, nonbinding proteins have two similar characters on nonbinding residues. Therefore, the definition of NBP(1) and NBP(2) is similar to that of BP(1) and BP(2):

(3) $$\begin{array}{c} \text{NBP}\left(1\right)=\frac{\sum_{i=1}^{t}\text{RI}\left(i\right)}{10N}, \end{array}$$

where *N* is the number of amino acids in this protein, *t* is the number of nonbinding residues, and RI(*i*) is the reliability index of prediction on nonbinding residue *i* by DNABR:

(4) $$\begin{array}{c} \text{NBP}\left(2\right)=\frac{\sum_{i=1}^{N-1}2^{-i+1}\sum_{k=1}^{t(i)}\bar{\text{RI}}\left(k\right)}{10\left(N-1\right)}, \end{array}$$

where *N* is the number of amino acids in this protein, *t*(*i*) is the number of two nonbinding residues with the distance *i*, and $\bar{\text{RI}}(k)$ is the mean of reliability index for nonbinding residue *k* and nonbinding residue *k* + *i*.

The vector size for BP features and NBP feature is 4-dimensional.

#### 2.2.2. Physicochemical Property (PP)

Physicochemical property feature was usually used in the prediction of DNA/RNA-binding protein [5, 10, 11, 19, 20], identification of protein-protein interaction [21], protein fold recognition [22], and protein family classification [23]. This feature was constructed from amino acid composition and six biological properties of each amino acid including hydrophobicity, polarity, polarizability, secondary structure, solvent accessibility, and normalized Van der Waals volume.

The global composition of each physicochemical property was described by three descriptors, composition index, transition index, and distribution index. Composition index is the percent of amino acid of a particular property. Transition index is the percent frequency of which amino acid of a particular property is followed by amino acid of a different property. Distribution index measures the percent of length of a query protein within which the first 25%, 50%, 75%, and 100% of the amino acid of a particular property are located respectively. Detail information of physicochemical property feature can be found in previous studies of proteins [5, 10, 11, 19, 20]. The vector dimensional of physicochemical property feature is 132.

#### 2.2.3. Evolutionary Information (EI)

Position-specific scoring matrix (PSSM) which represents evolutionary information of amino acid sequences was used mostly in the prediction of DNA-binding residues [24–29] and plays an important role in distinguishing DNA-binding residues from nonbinding residues in those researches. Therefore, we considered to apply PSSM to identify DNA-binding protein in this research. PSSM scores are generated by PSI-BLAST [30] to search against the nonredundant dataset of amino acid sequences in NCBI, and 20 values are obtained for each sequence position. If the protein has *N* amino acids, the feature vector of PSSM is 20∗*N*. However, different proteins may have different number of amino acids. PSSM could not directly be used as a feature in the prediction work based on machine learning method. In order to convert variable feature vector into fixed vector, we improved PSSM in following step.

First, we normalized the values of PSSM using formula as follows:

(5) $$\begin{array}{c} p\left(x\right)=\frac{1}{1+\exp\left(-x\right)}. \end{array}$$

Second, we pooled all rows which belong to the same amino acid in this PSSM and together to form a new matrix. Then we obtained 20 new matrices with the size *Na*∗20, where *Na* is the number of amino acids of type *a*. 

Third, we converted each new matrix to a vector. We added all values in each column in new matrices. For each new matrix, we produced a 20-dimensional vector. Then we obtained 20∗20 = 400 dimension vector which represents feature of evolutionary information in this work.

### 2.3. Algorithms for Classification

Five machine learning algorithms were used in our study to select a best-performance algorithm to identify DNA-binding proteins: support vector machine-sequential minimal optimization (SVM-SMO) [31], simple logistic regression [32], random forest [33], naive Bayes [34], and decision tree [35].

Support vector machine (SVM) [36] is a supervise machine learning algorithm and widely applied in classification researches. The principle of SVM is to find a hyperplane as a segmentation of two classes to minimize the classified error. Sequential minimal optimization (SMO) is an algorithm for training support vector machine to efficiently solve the optimization problem. Simple logistic regression is a statistical model suitable for probabilistic binary classification. Random forest is a classification algorithm that uses an ensemble of tree-structured classifiers. Naive Bayes classifier technique is a simple probabilistic classifier based on applying Bayesian theorem independence assumptions and is particularly suited when the dimensionality of the inputs is high. Decision tree classifier generates tree-like graph or model of decisions for classification. It is developed using the classification and regression trees method. 

These machine learning algorithms are implemented by WEKA software (http://www.cs.waikato.ac.nz/~ml/weka/) [37] which provides a collection of machine learning algorithms for data mining tasks. 

### 2.4. Model Evaluation Procedure

To obtain a reliable result with low mean square error, *k*-fold cross validation was always used in empirical works [7, 8]. In this study, 5-fold cross validation method was used to access the performance of each classifier on the main dataset. The main dataset was randomly divided into 5 equal parts. Each run of cross validation is comprised of one part as the independent test dataset and remaining 4 parts as the training dataset. For each classification, performance evaluations of 5 repetitions of 5-fold cross validation were averaged and calculated. 

The following performance evaluations were calculated: accuracy, sensitivity, specificity, and Matthew correlation coefficient (MCC) [4]. Equations of the performance evaluations were represented as follows:

(6) $$\begin{array}{c} \text{Accuary}=\frac{\text{TP}+\text{TN}}{\text{TP}+\text{FP}+\text{TN}+\text{FN}},\\ \text{Sensitivity}=\frac{\text{TP}}{\text{TP}+\text{FN}},\\ \text{Specificity}=\frac{\text{TN}}{\text{TN}+\text{FP}},\\ \text{MCC}=\frac{\text{TP}\times \text{TN}-\text{FP}\times \text{FN}}{\sqrt{\left(\text{TP}+\text{FP}\right)\left(\text{TN}+\text{FN}\right)\left(\text{TP}+\text{FN}\right)\left(\text{TN}+\text{FP}\right)}}. \end{array}$$

ROC curve is widely accepted and effective method to compare the overall prediction performance of different classifier method. An ROC curve is constructed by plotting the sensitivity versus 1-specificity for varying cutoff values. The area under the curve (AUC) is the evaluation criteria for the classifier. Therefore, we used AUC values to compare our work with previous studies [15, 16].

## 3. Result and Discussion

### 3.1. Performance of Various Machine Learning Algorithms

The performance is shown in Table 2 for predicting DNA-binding proteins based on support vector machine-sequential minimal optimization (SVM-SMO), simple logistic regression, random forest, naive Bayes, and decision tree with 5-fold cross validation on main dataset. As shown in Table 2, the results demonstrate that SVM-SMO classifier achieves the best performance. The combination of all features achieves the best performance with accuracy, sensitivity, specificity, Matthew correlation coefficient, and AUC equal to 89.6%, 88.4%, 90.8%, 0.67, and 0.90, respectively. The performance of simple logistic regression classifier is slightly worse than that of SVM-SMO classifier but much better than other three classifiers with 88.3% accuracy, 86.7% sensitivity, 90.2% specificity, 0.66 Matthew correlation coefficient, and 0.88 AUC. Naive Bayes classifier achieves the worst performance which predicted DNA-binding proteins at 84.3% accuracy with Matthew's correlation coefficient of 0.59, and with a sensitivity of 82.6% and a specificity of 86.0%. The performance of random forest classifier is better than decision tree classifier. Considering the performance, we chose SVM-SMO classifier to identify DNA-binding proteins in our research.

### 3.2. Importance of Novel Attributes: Binding Propensity and Nonbinding Propensity

The novel attributes: binding propensity (BP) and nonbinding propensity (NBP), were firstly proposed in this research. Those two features were constructed by prediction results from our earlier developed prediction model DNABR which has excellent prediction performance in DNA-binding residues. In order to know the importance of those two features, BP and NB were combined with evolutionary information feature (EI) and physicochemical property feature (PP) to construct DNA-binding proteins prediction model using SVM-SMO algorithm, respectively. Seen from Table 2, when BP and NBP were combined with PP, the value of accuracy significantly increased and achieved 85.6%. The similar result were appeared when BP and NBP were combined with EI, and the value of accuracy increased considerably to 87.3% with MCC 0.66. Those results which were evaluated by 5-fold cross validation proved that BP and NBP play a significant role in distinguishing binding proteins from nonbinding proteins. 

**Figure 1:**
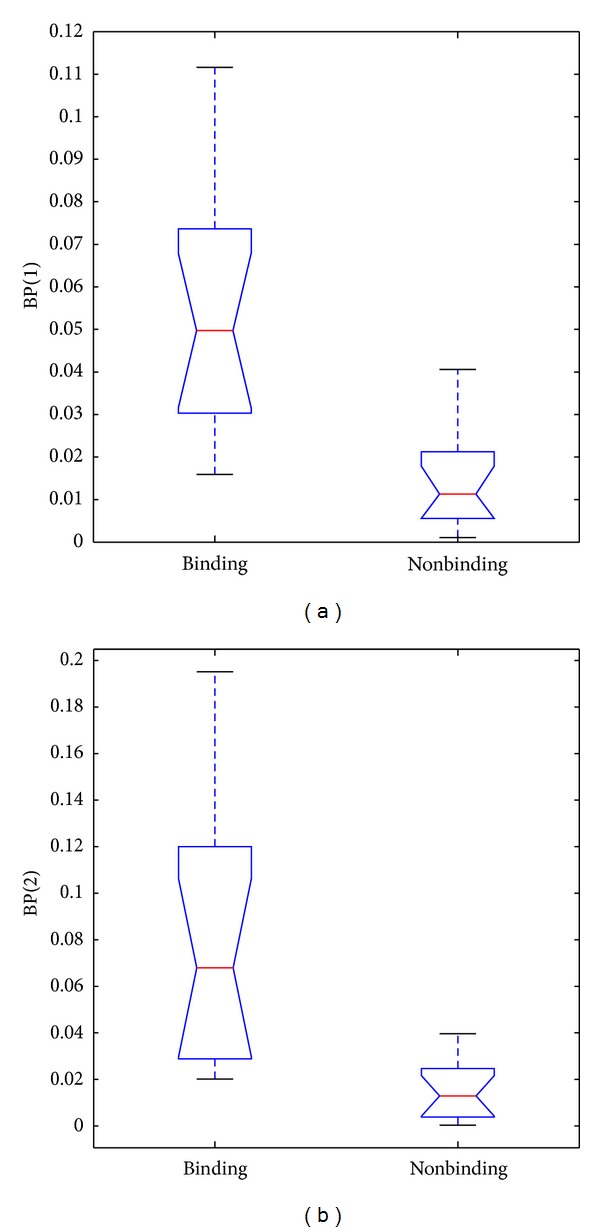
Box plots of the two components of BP feature for binding and nonbinding proteins. (a) BP(1); (b) BP(2).

**Figure 2:**
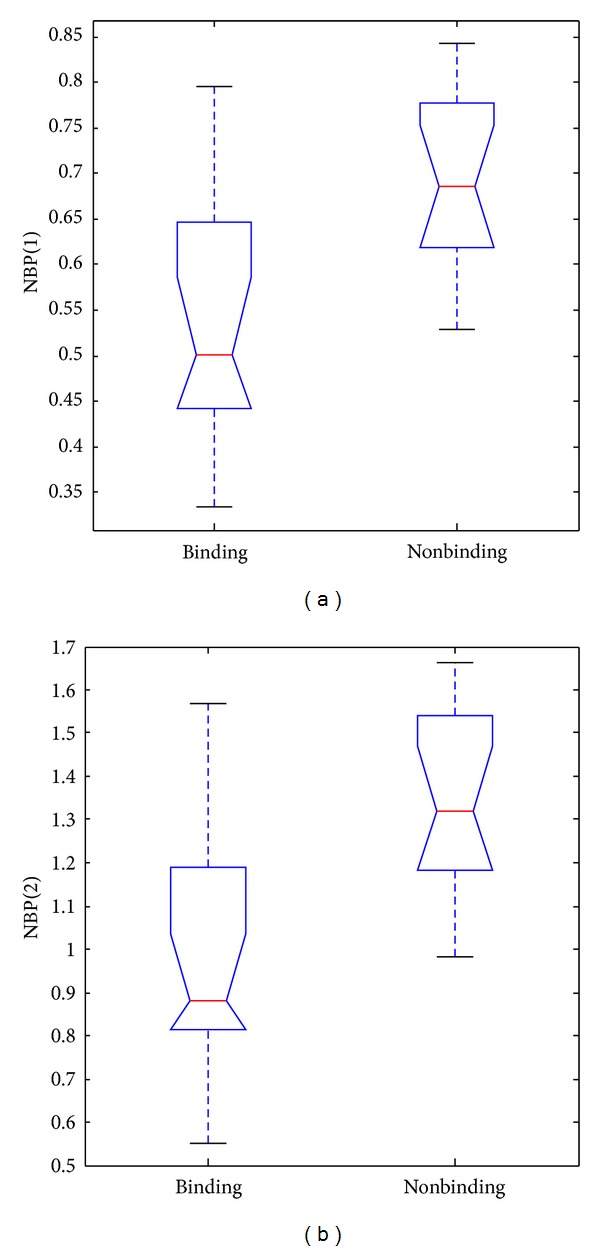
Box plots of the two components of NBP feature for binding and nonbinding proteins. (a) NBP(1); (b) NBP(2).

 Figures 1(a) and 1(b) present that binding and nonbinding proteins show contrasting behavior in terms of two components of BP feature. Figures 2(a) and 2(b) also show significant difference between binding and nonbinding proteins in terms of two components of NBP feature. We also calculated the *P* values of two BP components and two NBP components to measure the ability to separate the binding proteins from the nonbinding ones. Each of them was less than 0.00005. These results also proved that BP and NBP play a vital role in achieving excellent performance in our study. 

 The character of DNA-binding proteins and nonbinding proteins can illuminate the importance of BP and NBP features. (1) It is obvious that each DNA-binding protein has several residues which bind to DNA. DNA-binding residues should be much more in DNA-binding proteins in comparison to nonbinding proteins. The structural distribution of DNA-binding residues also has some regular pattern, such as DNA-binding residues which tend to gather together spatially on the surface of DNA-binding protein [38]. Two components of BP feature revealed the character of DNA-binding proteins for sequence level and spatial level, respectively. (2) On the contrary, proportion of nonbinding residues should be much higher for nonbinding proteins in comparison to DNA-binding proteins. Hence it is rational to propose NBP feature. Therefore, we predicted DNA-binding residues and nonbinding residues in a query protein by DNABR model which achieved the best performance in comparison to previous similar works. It was observed that using information of binding and nonbinding residues worked well as we expected. BP and NBP can successfully discriminate between DNA-binding proteins and nonbinding proteins.

### 3.3. Performance Comparison with Other Methods

**Figure 3:**
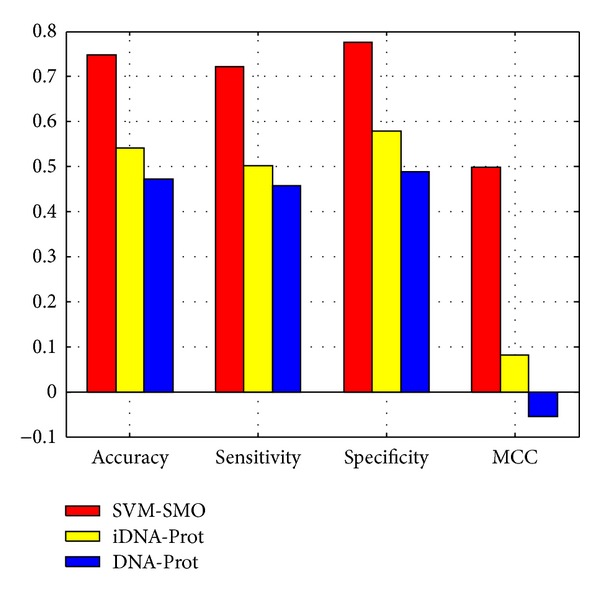
Three classifiers were tested on the same testing dataset TeD_2264. The predictors have the following accuracy value: our SVM-SMO 74.88%, iDNA-Prot 54.06%, and DNA-Prot 47.30%; sensitivity: our SVM-SMO 72.22%, iDNA-Prot 50.22%, and DNA-Prot 45.72%; specificity: our SVM-SMO 77.55%, iDNA-Prot 57.88%, and DNA-Prot 48.87%; MCC: our SVM-SMO 0.4981, iDNA-Prot0.0814, and DNA-Prot −0.0541.

iDNA-Prot (http://www.jci-bioinfo.cn/iDNA-Prot) [16] predicts a query protein as a DNA-binding protein or a non-DNA-binding protein only based on its amino acid sequence information. The iDNA-Prot was constructed by incorporating the features into the general form of pseudo amino acid composition that was extracted from protein sequences via the “grey model” and by adopting the random forest model. The overall success rate by iDNA-Prot was 83.96%. Kumar et al. proposed a random forest method, DNA-Prot, to identify DNA-binding proteins from protein sequence [15]. DNA-Prot was proposed to encode each protein sequence with 116 features by incorporating various physicochemical properties of amino acids. Using the dataset in research [15] DNA-Prot could identify DNA-binding proteins from non-DNA-binding proteins with more than 80% accuracy. As mentioned in Section 2, to evaluate the performance of our method against the state-of-the-art algorithms, that is, iDNA-Prot and DNA-Prot, an independent test dataset TeD_2664 was obtained by selecting 1332 DNA-binding proteins and 1332 nonbinding proteins randomly from “Positive” dataset and “Negative subset” dataset, respectively. The iDNA-Prot prediction results of 2664 proteins in TeD_2664 dataset were obtained by web-server (http://www.jci-bioinfo.cn/iDNA-Prot). TeD_2664 dataset used to test DNA-Prot and the prediction results can be obtained from its standalone version (http://www3.ntu.edu.sg/home/EPNSugan/index_files/dnaprot.htm). Here we also trained another SVM-SMO model on training dataset TrD_10642 using the same strategy as original SVM-SMO model with all features and the model used to identify DNA-binding proteins in the TeD_2664 dataset. As shown in Figure 3, the accuracy is 74.88%, 54.06%, and 47.30% for our SVM-SMO model, iDNA-Prot, and DNA-Prot, respectively. The SVM-SMO model attained 72.22% sensitivity, 77.55% specificity, and 0.4981 MCC. The results show that the SVM-SMO model achieves the best performance.

## 4. Conclusions

In this paper, we present a novel approach based on support vector machine-sequential minimal optimization (SVM-SMO) and a hybrid feature for the prediction of DNA-binding proteins using only the primary sequence of a protein. Two novel attributes, denoting DNA-binding propensity and nonbinding propensity, were constructed by DNA-binding residues information and nonbinding residues information. The results prove that these two attributes markedly improve the prediction performance. The SVM-SMO model with the hybrid feature that includes two novel attributes, evolutionary information feature, and physicochemical property feature has a prediction accuracy of 89.6% with MCC of 0.67. We believe that our SVM-SMO method is currently the most effective method for predicting DNA-binding proteins using only sequence information.

## Supplementary Material

The training dataset (TrD_10642) and test dataset (TeD_2664) used in this paper are listed in the Supplementary Material. Because of the limited space, only the listed accession numbers of UniProtKB/Swiss-Prot entries are listed.

## Figures and Tables

**Table 1:** The distribution of proteins in main dataset, training dataset, and independent test dataset.

Dataset	Number of binding proteins	Number of nonbinding proteins	Total number of proteins
Main dataset	6653	6653	**13306**

Training dataset (TrD_10642)	5321	5321	**10642**
Independent test dataset (TeD_2664)	1332	1332	**2664**

**Table 2:** The performance of different kinds of feature descriptors with various machine learning algorithms based on main dataset using 5-fold cross-validation.

Machine learning algorithm	Feature descriptor					
	PP	EI	BP + NBP + PP	BP + NBP + EI	PP + EI	BP + NBP + PP + EI
	Accuracy (%)					
SVM-SMO	83.2	85.7	85.6	87.3	87.3	89.6
Simple logistic regression	81.8	84.2	84.2	87.0	85.7	88.3
Random forest	81.3	84.3	83.5	86.7	85.9	88.1
Naive bayes	78.6	77.2	82.8	82.3	82.6	84.3
Decision tree	80.2	82.5	82.6	84.4	84.1	86.2

	Sensitivity (%)					
SVM-SMO	82.4	84.9	84.4	86.5	85.8	88.4
Simple logistic regression	80.7	83.1	82.3	84.4	85.6	86.7
Random forest	81.1	83.6	82.8	86.0	85.3	86.2
Naive bayes	76.9	76.1	79.4	80.8	81.1	82.6
Decision tree	78.6	80.4	81.7	82.7	82.5	84.7

	Specificity (%)					
SVM-SMO	84.6	86.3	86.7	88.2	88.6	90.8
Simple logistic regression	82.9	85.5	86.0	88.8	85.9	90.2
Random forest	81.6	85.2	84.1	87.5	86.3	90.0
Naive bayes	80.2	78.5	85.6	83.8	84.7	86.0
Decision tree	81.8	84.7	83.5	86.2	85.7	87.7

	Matthew correlation coefficient					
SVM-SMO	0.55	0.58	0.62	0.66	0.66	0.67
Simple logistic regression	0.56	0.55	0.64	0.62	0.64	0.66
Random forest	0.55	0.56	0.60	0.62	0.63	0.66
Naive bayes	0.52	0.49	0.56	0.53	0.54	0.59
Decision tree	0.53	0.55	0.61	0.63	0.62	0.64

	AUC					
SVM-SMO	0.83	0.86	0.86	0.88	0.87	0.90
Simple logistic regression	0.83	0.84	0.85	0.86	0.85	0.88
Random forest	0.81	0.84	0.84	0.86	0.85	0.87
Naive bayes	0.78	0.76	0.80	0.79	0.80	0.82
Decision tree	0.80	0.82	0.83	0.84	0.84	0.86

BP: binding propensity feature; NBP: nonbinding propensity feature; PP: physicochemical property feature; EI: evolutionary information feature.
