# Supplementary material for: Identification of DNA-Binding Proteins Using Support Vector Machine with Sequence Information
Source: Comput Math Methods Med. 2013 Sep 16;2013:524502. doi: 10.1155/2013/524502 (PMC3787635; doi:10.1155/2013/524502)
Supplement: Supplementary file 1 — The training dataset (TrD_10642) and test dataset (TeD_2664) used in this paper are listed in the Supplementary Material. Because of the limited space, only the listed accession numbers of UniProtKB/Swiss-Prot entries are listed. [file 524502.f1.pdf]

Training dataset (TrD_10642)

5321 DNA-binding proteins

P16375	P16376	Q8V2N7	O41107	Q01980	P67664	P43463	Q9R9M8
P29552	O76762	P15856	Q05007	P29555	Q26430	P29556	Q07961
P33293	P14164	Q02486	P77744	A0MES8	C7J2Z1	Q9SJN0	P00519
Q9USY4	Q9FGF8	P08874	Q24174	P39970	P40535	P32104	Q96AP0
Q9P8W3	Q96WN6	O14258	P21192	O31551	P28614	P78704	P0ACT0
P0C5S3	P87000	C5GF27	A7EAG1	P19219	P06134	Q5XEA3	Q3AA35
B8FXD7	B0TDI0	Q1GAA9	B2GEY4	Q38X69	B1MZM2	Q2RL77	B2A610
A4W0M7	Q67MD5	P23477	P71073	O06008	P33234	Q8CHC8	Q9H2P0
P07248	P40634	Q8IUX7	Q7SXV2	Q6GR30	P39413	P55197	P51827
P41765	P52957	Q9ZN78	P25941	Q2TA45	P43464	Q38841	Q38847
Q7XJK6	Q4PSU4	Q9FKK2	Q9FJK3	Q9C6V4	Q7X9H9	Q9C633	Q42464
P29369	Q5HEG2	O02219	Q5TGY3	A9YTQ3	O44712	Q8R4S5	P0A3J5
Q9LES3	Q9M7Q3	P33224	O95831	Q1PFE1	Q6PQQ3	Q52QU2	Q91VJ1
Q5VK71	Q7Z591	Q9YAX2	Q971T6	Q9FHA2	P04546	Q40359	P15276
Q10630	O31249	Q0VKZ4	Q9L4M7	Q57S50	P33997	Q04778	Q91574
Q9H161	Q26657	Q06453	P40641	P24734	Q9URT4	Q9ZVX2	P41772
Q84BZ4	P10275	P49699	P79775	E9PGG2	Q9NLA3	Q9CQM6	Q07683
Q38914	P19655	P56095	Q01663	Q9N0N3	Q76HI7	Q94AN4	Q8GWK2
Q9UBZ4	Q06481	Q06335	Q9SAK5	Q6LA43	Q9FJ16	P29673	P61798
Q8MSG8	O74859	Q08702	P52659	P07642	Q9KBQ0	Q24248	A7X715
Q01582	Q5NB85	Q94JM3	O23661	Q9ZTX9	P93022	Q6YVY0	Q01K26
Q7XSS9	Q84WU6	Q653H7	Q0D9R7	Q9LP07	A6NJG6	P07249	P05085
A9WQ89	O14497	Q62431	A6NKF2	Q9JKB5	Q03989	E7F888	Q14865
Q9LDD4	Q68CP9	Q940Y3	Q6NQ79	Q9Y4B4	A4IHD2	A7ZKT2	O15945
Q04052	Q9KJC4	Q9FXD6	P62598	Q9FGT7	Q9M9B9	Q940D0	Q9LZJ8
P13027	P46003	P15905	P45949	O42115	A6YP92	P24242	P50553
O35885	Q9NQ33	Q6XD76	Q7RTU5	P96677	P40467	Q6R0H0	P34233
P0ACI8	Q9LS40	P09774	P10083	Q8S9N6	Q4PSR7	Q9LZR0	Q9FN29
P0CJ65	B4JND4	P18846	P52890	P15336	Q09771	Q2KII1	Q6NW59
O70191	P18850	Q99941	Q8R0S1	Q9HFR4	P48731	P92953	P46667

Q8GXM7	Q9UTN9	Q9M3G7	Q62388	Q8N100	Q99NA2	P48987	Q9U7E0
P46100	P82798	P70486	P83946	Q9VXG8	Q9DE14	P0C7T5	P54253
Q8IRC7	Q9SSW1	P41696	Q9SSW2	O07920	P0C7L5	P22015	P15308
Q9W0K4	P97302	Q9BYV9	P97303	O07458	Q6NTS2	P22809	Q8VBU8
Q9VYG2	P22544	Q24256	Q9NY43	Q9HBU1	O08686	Q9N0M2	P22035
Q9NR55	P97876	E1BD44	Q9UIF9	Q9DE13	Q9UIF8	Q6PQQ4	Q7XH85
O06703	Q8WY36	P09081	P55924	Q9NYF8	Q8GWK7	Q38897	O56862
P29169	O68014	Q8G2M8	Q9L4K2	O23487	Q84TK1	Q8W2F2	Q8W2F1
O23090	Q8S3F1	Q9SVU6	Q9T072	Q9S7Y1	Q9LTC7	Q2HIV9	Q9FLI1
Q9SN74	Q8VZ02	Q9CAA9	Q9XEF0	Q8LEG1	Q9M128	Q3EAI1	Q8GY61
Q9ZUG9	Q8S3D1	O81037	Q56XR0	A4D998	Q9LK48	Q9FJL4	Q9LV17
Q9LSQ3	Q84WK0	Q9FJ00	Q8S3D2	Q9FIX5	Q9LSL1	Q9FXA3	Q9C7T4
Q9FYE6	Q8VZ22	O80674	Q5XVH0	Q9FYJ6	Q94JL3	Q9LT67	Q9LRJ4
Q9FLI0	Q9LT23	Q9C690	Q8GXT3	Q9STJ6	Q7XHI7	Q8H102	Q9ZW81
P0CB25	Q9CA64	Q93W88	Q9M041	Q9FGB0	Q9LSN7	Q9C8Z9	Q9M9L6
A3KNX5	Q7RTU4	Q9QYC3	Q8NDY6	Q9C0J9	Q8UAA8	Q9PFB1	Q9LEZ3
Q9FMB6	P20384	Q9FLU1	E0U174	P46363	P42179	P33651	P06555
Q9FXG8	Q1PFD1	Q9SJ56	B3LP78	Q94KL5	Q8S897	O65685	Q9SJJ3
O18017	Q9I920	Q9VGI8	P54132	Q8WYA1	P39075	Q01954	Q9KPS0
Q8L999	O82286	Q0JXE7	P37335	P56158	Q9U903	Q01295	O01409
Q95153	P51587	P10069	O36398	Q66652	Q01012	Q91998	Q01108
O08335	P94403	Q6RFL5	Q04787	Q24266	P40408	P52665	P52666
P24649	P39736	P55965	P36627	Q9FGX2	Q9FUD3	Q1HVG1	O22763
Q9M1G6	Q54WU0	Q54Y73	Q55E93	Q54RZ9	Q86AF3	Q54GG8	Q554P0
Q54Q90	Q54T17	Q54LU5	Q54GH0	Q54IJ9	Q54GH2	Q54GG9	Q9LN63
Q6P1N0	Q9C7P1	Q94JI5	Q9LQM3	Q3ECU8	Q6H7U2	Q9C9N3	Q9C9F5
Q6YVX9	Q9ZUM0	Q9SJ41	Q6K687	Q6K977	Q8GX84	Q5VR07	Q0DVU4
Q10MN8	Q10M00	Q8L7S3	Q10EL1	O48772	Q84VG7	Q0JDM0	Q5PP65
Q7XSB2	Q9ZWA1	Q7XM16	O22243	Q0J952	Q6AT25	Q6L4N4	Q9C7C3
Q941Q3	Q65X92	Q9LIH5	Q9LT81	Q0JP11	Q93XW7	Q9LVX1	Q5ZA07
Q0DBW8	Q9STM4	Q5Z5Q3	Q9SD34	Q0DA50	Q9SV09	Q5Z807	Q8GW05
Q6Z358	Q9FNG6	Q84ZT0	Q9FL40	Q6ZK57	Q94CJ8	Q6YYC0	Q8L7N8

Q69KP0	Q9FKR9	Q69NK8	Q9SY74	Q9FNZ1	A3CEM4	Q9LTS7	Q2QTY2
Q2QPW2	Q56XU4	Q5NAW2	Q3ED78	Q5ZDJ6	Q9C7P4	P30339	P23890
P26950	P14984	Q8QME6	P54089	Q91EK1	P29178	P29073	O56129
Q89551	Q88886	P31616	P27256	Q06909	Q97Y84	P38036	G3ECR1
Q7M3M8	P39113	P54269	Q95RV2	O74954	O74412	P49379	P17106
P40969	P35203	O43102	Q1I490	Q46995	Q03513	P25144	P37517
Q8N726	O77618	Q64364	Q09184	P92948	Q54WZ0	Q9YFU8	O27636
Q97WM8	P81413	Q8W1E3	Q93ZL5	Q9SEZ3	Q9HTH5	Q8R4E9	Q9I9A7
Q05095	Q99626	O14627	P81193	P53566	O02755	P49716	Q15744
Q02638	P34618	A8AHC2	Q756C3	Q6BLT3	Q03654	O87590	B2RD01
Q0MXD1	Q7RXR3	Q0MXD0	Q9Y812	P48988	P49451	Q03188	Q9USR9
Q9D084	F1NPG5	Q96BT3	Q561R1	Q5EE01	A8MT69	A8WW61	Q20646
Q94126	P20385	O02326	Q8N884	P35168	Q00858	P43634	P17410
Q7KU24	P32657	Q22516	O16102	Q8TD26	Q06A37	B0R0I6	Q09XV5
Q966L8	Q9NK54	A7UXD4	A7ERG1	Q10103	O42934	P33647	P08365
P40685	Q07783	Q9U1H0	Q96RK0	Q9V6Q2	P17867	P52688	P39127
P19538	O77027	P30373	Q95YE9	O61735	Q8PQ45	Q9HAW4	Q10241
P14204	O53922	Q54JH6	Q94F87	Q94F88	Q6NPP4	Q80Y50	Q9FYG2
Q9LSP8	P67732	P53996	P20482	P37978	Q9CJ45	Q9BQW3	P75974
A6QNQ6	F1R983	Q99708	O74986	P40396	P75952	P25921	P62536
P14269	P03267	P41046	Q24478	Q00096	O22059	P22058	P43079
Q99090	Q66HA2	A2VD01	Q91XE9	Q502F0	P0ACP3	Q32M00	Q8L925
Q61817	Q02930	Q8K1L0	P29747	P51984	P51985	Q03060	Q9YEY2
A3MUI7	P45815	Q9SUQ2	Q9FK12	Q6PG95	O76906	P32027	B3LSR0
Q54YU6	Q5IZC5	Q7NMD1	Q651U1	P53968	Q57824	Q97Y88	P62604
P52106	P37338	P71635	E7S4M0	Q7U0Y5	Q41188	Q94C69	P41018
Q45100	Q5R638	Q9H175	P59055	P43460	D0EL35	A0JMF1	Q2NKJ3
Q9R1D1	P0C1D3	Q9USQ1	Q6NU40	P52958	P52959	P0CG11	Q65ZA6
P89105	Q88XZ6	O00841	O07586	O94588	P15315	P41817	Q9BL02
P39881	P53564	P53565	Q752S4	Q9P6R8	Q4P400	Q6CB23	P53769
O61734	O34527	Q8X4M5	P22697	P52675	P39647	P27369	Q55854
A0R8S0	P28875	O31844	P21969	Q925Q8	Q09555	P26343	P21657

Q16IB4	Q29CW2	Q82IW0	P13483	P32325	P0A3H7	Q92HL4	Q44625
Q9RZ89	P02345	O83278	Q9PQK9	Q32PF6	A5PKG8	Q17QR5	Q91975
Q14AI0	Q09732	P45876	Q8X5P3	A1A4K3	Q21554	B0M0P5	O13807
Q5ZJL7	Q99J79	Q755A0	Q08949	Q9XFH4	Q9RX92	Q9RY80	Q96FC9
Q0IIK5	Q9JJY4	O00571	Q24180	Q8QZV0	Q75DE9	Q5AMM4	C4YHS3
C4Y1P0	Q6CTH5	A5E406	C5E4K0	P15263	P54662	P41894	Q7TNV0
P0ACK7	P95193	P07548	P16931	P45512	Q9RAJ1	Q9CIV9	O70133
P06965	Q9SNW8	Q24533	P98149	Q54ER9	B6VQA1	P54394	Q8X7X9
P29741	Q5HP88	Q47150	Q82EA6	B2SB45	Q8S9H7	P53772	P20009
P40764	Q64204	P87394	P56179	Q9SR66	O49498	Q9PUE0	Q9QZ59
P57690	Q9Y5R5	Q80WT2	A2A9I7	Q96SC8	Q6DG03	Q8CE22	B0I1G7
P13123	Q6ZQJ5	Q9URU2	P38859	O66659	B8FFL8	Q6ARL8	B8DN19
Q72WD6	P24116	P35889	Q59549	Q6YRL3	P34028	A0LE53	C5BKL9
P35907	P07908	O78411	O25916	Q9F5P4	P46394	P75539	P51333
Q1RI04	O83097	P03264	Q65956	P05662	P36704	Q03444	P28932
Q18LF9	P17147	Q6UDK2	P32761	Q9Y6K1	O88509	P34881	Q9Z330
P15017	Q06259	P68349	Q9FZA4	Q9SX97	Q84JQ8	Q8LE43	Q9ZV33
Q9ZPY0	Q94AR6	Q39088	Q9SVC5	Q43385	Q9M161	Q9SUA9	O49550
Q9LZ56	Q84TE9	Q8LDR0	Q9FM03	Q9NDJ2	P15330	Q8TEK3	Q04089
Q5UZ40	Q18ER3	Q9V2F4	P81409	O28484	B0R7U1	Q58113	O57863
Q9FNY3	Q6FSK8	P0CN25	Q6BQR8	Q6C030	Q9FNY2	Q26263	O67779
O08307	P52027	Q9ZJE9	P09804	Q92GB7	Q9RAA9	Q55971	P74933
P05468	P63988	Q98JM5	Q8YC76	A3DH61	Q8REB0	B0R4P0	Q8EQ56
Q9AK82	Q4JB80	Q73P36	B1AJ79	P13382	P28340	Q54RD4	A7YWS7
Q54Y85	Q3SZN5	Q75JQ9	Q9CQ36	Q92076	P54098	Q0VC30	Q9W6G7
Q12704	P15801	O93745	P26811	Q07635	P95979	Q54SV8	P26019
O48653	Q27152	Q6DRD3	Q9UGP5	P42494	Q7T6Y4	O60673	A4ZU91
Q64751	Q88469	O36363	P43139	O64235	P03680	P10479	Q38087
P06225	P20311	P19822	P30319	P03162	Q6GZR5	P08546	P07918
P28859	Q196U0	Q9QSK2	Q58295	P52025	Q5UQR0	P30318	Q84173
P30321	A7U6F2	A7U6F3	P61875	P77933	Q51334	Q85428	O70736
O33845	P74918	P30317	Q4U3V0	P12899	P11292	P30322	P22374

P10582	P33537	P33538	Q01529	P14284	A6NFQ7	Q9RS64	Q8YQL3
A0R692	Q8U1L3	Q6REU5	Q0J3Y6	A2WL19	Q5W6R4	Q8LFR2	Q84ZA1
Q65WX1	O80917	Q10R18	Q9SVX5	Q9SIZ0	Q9LYD3	A6NNA5	Q4H3P5
Q8MQH7	Q9M548	Q7PN68	P24274	P46068	P55265	Q99MU3	P23023
Q4JV96	P0CJ85	O43812	O75505	Q6RFH8	A6NLW8	O81900	Q01094
P56931	O00716	Q16254	Q15329	Q08DY6	E1BE02	Q5RIX9	F1QZ88
Q9FV71	Q9FV70	Q9LFQ9	Q8LSZ4	Q66K89	P11536	Q7M3M6	P17672
P17671	P50239	O77245	P45447	Q0SN44	P06846	Q1HVF7	Q46967
P32644	O69280	P49880	P49881	P34021	O18531	B3LNB2	O80337
Q9FKG1	Q9LY05	Q9SZ06	Q70II3	Q9LYU3	Q9LY29	Q8GW17	Q9M9B2
Q9LUA2	Q9FGC9	Q38Q40	P29692	Q09228	Q19849	P17486	P15370
P18146	P26634	P11161	Q05215	Q32LN0	O23115	O23116	O24606
P32519	Q15723	Q4V7E1	Q99607	Q04688	P41969	P28324	P28515
Q8WYP5	Q5U249	P25032	P66800	Q6PJG2	P15858	P18488	Q04741
Q58030	P63541	Q9WYK0	P64159	A8AJA5	P27871	P25696	P0ACT3
Q96L91	Q8CHI8	P97481	P30198	A2BGR3	Q2NKX8	Q9MA98	Q55GG6
P14629	Q03468	Q94AW5	Q9CAP4	Q9C591	Q9S7L5	O80542	Q84XB3
Q9LQ28	Q1ECI2	Q9FJ90	Q9CAN9	Q9LU18	O80654	Q9ZQP3	Q52QU1
Q9SKT1	Q9SIE4	Q40478	Q9C7W2	Q9C995	O22259	Q8H0T5	Q9ZWA2
Q9LDE4	Q9M8M5	Q6J9Q2	Q9FZ90	O49515	Q9LND1	Q9LTC6	P93822
Q8L9K1	Q56XP9	P04892	P70459	Q01414	O95718	P62508	Q6C5G3
Q6FML7	Q6BY37	A5DF43	C4R1K8	D8Q8R5	Q4PD88	P26337	Q04635
Q01797	P25932	Q01068	P96228	Q9SAD4	Q91424	P49886	P16058
Q9FYK5	Q9PTU5	Q9TTE5	P57781	O74804	P17214	P52667	Q8N693
Q9YVX9	O93120	Q9YW29	Q9QB84	P38071	P13474	P15062	P18756
P29773	P29774	P29775	P29776	Q6ZN32	P28322	P41161	P97360
P36547	P06602	P0ACZ6	B7ZRU9	Q24312	O67550	Q9KAV6	P54161
Q49406	Q98PK2	Q10699	A8R3S7	Q8L6Z7	Q54ED2	Q24558	Q9UQ84
P53695	P39875	Q9FKK6	Q75A64	Q59ZZ6	Q6FJK6	C5M3V9	C4Y732
Q6BKP5	C4R7Q5	A3M0E6	Q9Y7L4	B3LMZ5	Q84T68	P96440	P26993
Q9J5B7	P68454	P20222	A7ZUI3	A5F6Z2	Q56YJ8	Q9NVI1	Q8IYD8
P23774	B0K9Y3	Q8JIT7	Q9USU3	O93870	Q8X229	Q02360	Q02361

P32029	Q84JK2	Q47129	P23484	P00214	Q923Z4	Q9VGJ5	A3FEM2
A0PJY2	Q9ZVH0	P87114	O74842	O14270	P39521	P19323	Q6QHK4
P37928	P0AEL9	P18809	P64130	Q8PQ10	Q5F881	Q5F882	Q0V7X4
P26488	P13295	Q26486	P40466	P41813	Q19802	P14734	P17899
Q39LF8	D5W6H7	Q0K2R3	Q46TH1	Q2NX28	C5CXR6	Q39LF7	Q9X600
P29248	P52618	Q59QW5	P29284	O13360	P35692	P51007	P24290
Q46158	Q9CMY2	P60622	Q8IX07	Q8WW38	B4JYN3	B4NBL5	B4PPK2
P23050	Q9Z2Q8	P01102	O02761	Q99853	Q5VYV0	Q12948	Q63246
O60548	Q12950	Q60688	Q5J3Q5	Q9PTK2	O54743	Q9VS05	Q1A1A6
Q9I9E1	O88621	P70056	B5RHS5	Q8BUR3	P85037	Q01167	Q12952
Q08050	O15353	Q28F43	O00409	Q3BJS0	O43524	Q70KY4	O16850
Q5W1J5	Q8HZ00	Q9BZS1	Q9DBY0	Q6PJQ5	O43638	Q0VG06	O80358
Q5UQ00	Q4FUU7	P35693	Q8X835	O31713	Q8IN81	P32152	Q2YJF8
Q8XRH0	Q9JUK9	Q9RXB5	Q8ZS46	O83045	Q8G4H3	O51272	Q0PA12
Q8KBK0	Q8FPC1	P45264	O25722	O05560	Q8ZQD5	Q8EER3	Q8E418
O83964	Q87QP4	Q8ZGC7	P49867	P48590	Q3U0V1	Q96I24	P0ACK8
P31380	O69450	P0A583	O28051	A8FKI7	P45599	Q55244	P74739
Q8J1X7	P35637	Q4VC44	Q9NU39	Q9PT68	Q66IG8	Q708W2	P32805
P25858	P46672	P15417	O80294	P03671	P03672	P68676	Q06546
P63206	P63202	Q9EYV5	Q10280	P03373	O56860	P10258	P03343
P04386	Q06433	O84905	Q88JX7	P25748	A2WW87	O07008	Q8VZP4
Q9M1U2	Q8LG10	Q9LIB5	Q8LC79	P23768	P43574	Q5HZ36	Q8LC59
Q8W4H1	Q8H1G0	Q9LT45	P40209	P40569	Q8LAU9	P17678	P15976
P23769	Q8L4M6	Q9FH57	Q92908	O65515	Q9SV30	O82632	P52167
P36930	P42774	P42777	P36417	P52661	P25547	Q14549	P82976
P16383	Q9NP62	Q9VLA2	O75603	P03069	Q9PU65	P07261	O73673
P0A9F8	P34333	P11470	Q6DCW1	P70338	P39572	Q03833	P27900
P13360	P0ACL6	P94591	P55878	Q91690	P55879	P10070	P10075
Q8K1M4	Q7K0S9	Q9BZE0	Q6XP49	Q1BYA7	Q58815	P18494	P40759
Q05943	P20668	P54717	O87389	Q9Y692	O05509	P46833	Q06003
Q3KR53	Q28FE5	Q9U405	O51157	Q82I57	O83063	Q8ZJH2	Q8TE85
Q43472	F8DT92	Q9C469	P09083	P54366	A1YEY5	Q754J3	Q6FLG1

C4Y4V1	Q6CK33	A5DU46	C4QV17	B3LQ10	C5DX31	P31316	Q9JI57
A4IFA3	Q54L82	Q44929	P78347	Q8H181	P29588	P15081	P39143
Q9CAF6	P21557	Q49166	Q57532	P72065	P34030	P0C0Q9	Q44273
Q9FAX2	O33367	Q1RHT8	Q9H116	Q99282	Q94890	P40274	P40268
Q5RKG3	Q8IZA3	Q86QH9	Q86QI0	P22974	P22975	Q99284	Q18336
Q6FJX6	P32103	P84408	P37218	P12305	P53551	Q9XG56	P09589
A2Y5G8	Q9T0H7	P0C5Y9	Q54HV7	Q5Y2C3	Q8SSG3	P59890	P82897
Q6DRA6	Q9SGE3	P0C1H6	Q7Z2G1	Q8SRE2	Q9MBF7	P81195	P06353
Q9XG57	P40287	Q12753	Q8TFU8	P41546	O29910	P29303	O43593
Q8I7Z8	Q0VCE2	P0CE41	P53768	P06774	P40914	P36611	P13434
P79007	Q02516	P46665	P46604	Q02283	Q9SEZ1	Q9XYD3	P23922
Q8R316	Q54PU1	Q54LB3	Q54I65	Q54F11	P42585	Q86IH1	P55813
Q54ZP8	Q54YI1	Q54X22	Q54VB4	Q47141	Q01239	P25364	A8XA80
O33817	Q9PLI1	Q46397	Q8SW18	Q8SRR1	Q8SUB7	Q9FDX8	Q8SR09
Q8STR8	Q8SVT3	Q8SVT6	Q8SW50	Q7SI91	A1JI47	Q9FFI0	P51858
Q8UW74	Q9FHA7	Q9LXD8	O32215	Q6QB00	A6NFD8	Q7TS99	Q9BYK8
Q5IW40	C0H4W3	O57337	Q00P32	Q9HCC6	P70120	Q9BYE0	Q93CE9
P46118	Q9QUS4	Q7KM13	Q84MN7	Q93VB5	Q1PDN3	Q5Z6A4	Q9LUH8
Q9M1V5	P27343	Q7XRX3	Q652B0	Q6VBA4	Q942D6	Q6EUG4	P51979
Q9FE22	P0ACE5	D2KQB0	Q05502	Q9JLZ6	Q7N1I3	Q3KJK7	Q61221
Q9Y2N7	Q9KMG4	Q9KMA5	P67702	Q5PEB1	E1WAC0	P60008	Q9QYL0
Q9BQA5	Q8FHF3	Q8NCD3	Q02308	Q20561	Q18053	P90953	P34555
Q01622	P21523	Q92GN5	Q14527	P52695	P53547	P20269	P20268
Q94398	P56407	P41935	P17487	P12902	P20271	P34326	P15857
P26797	Q5ZKF4	Q32L68	P41779	P41936	P34663	Q9NLC2	P34522
Q23175	Q94166	Q94165	Q93352	Q19720	Q22812	Q18273	P15142
P17138	P0CY07	P0CY09	P13545	P10179	Q6NT76	P06116	P31537
P09065	P09076	O02491	Q05640	P02836	P09145	P23397	A9ZPC9
P09532	Q9LG02	Q941D1	Q09390	Q9MAT6	P40622	Q24537	Q23794
O49595	P10103	P07156	Q9SUP7	Q9SGS2	P40626	P11873	P40628
P82970	B4F777	Q12766	Q9UGU5	Q10370	P10035	Q00401	Q12039
P05527	Q03973	P22810	P31264	P0CY13	P58378	Q12398	P47175

Q03576	Q9DE09	Q9NP08	A2RU54	Q9VEI9	Q26656	Q6NU14	Q90867
P41235	Q14541	P49866	P42587	O14979	Q3SWU3	Q14103	Q3T0D0
P0AEV2	P0ACG0	P12258	Q8IX15	P20050	Q54UM1	Q9P2W1	P53187
A2Z1U1	Q67UE2	A2XDK5	A2XDD6	Q01I23	A2YGL9	A2XE76	P46605
Q8S7W9	Q01IK0	A2Z0Q0	A3BYC1	A2Y931	Q5QMZ9	Q84U86	P50901
Q9XH36	Q9XH35	Q99160	P09932	P62573	Q9JN69	Q56185	Q65LS8
P49870	P49869	P31396	P49868	Q24142	Q24143	P18182	P18184
P31778	P37929	P37930	P26316	Q00613	P22335	P38530	P38531
Q1HGE8	Q5ND04	Q40152	Q8GYY1	Q6K6S5	Q9S7U5	Q9LVW2	Q10PR4
O22230	Q9LV52	Q9UBD0	Q96LI6	Q8SS62	B7XIV9	P22121	C4V6H6
P41154	P10961	P0ACH0	P83211	P83183	P40183	Q10076	Q60420
P51514	O53478	O46339	Q8RVQ9	P31505	O18326	O96785	O46238
Q01778	Q01791	P26408	Q09811	Q7TMY8	P51593	P42505	P42507
P31310	P50210	P31271	Q08821	P31261	Q00056	P81192	P09635
P50208	P09637	P24061	P09634	P51783	Q98924	Q1KKX5	P31357
Q1KKX7	O13074	P14838	P14839	P23681	P09078	P18863	Q9PWL7
P28174	P18865	Q1KKU7	P31262	P49925	O42503	P18866	Q28601
Q9YGT0	P24341	P31277	P13544	Q1KKR7	P28356	Q1KKR8	O24646
Q8W191	Q8V518	P20499	O72902	Q9RQQ0	Q9LSE2	P16528	P29128
P28284	P29129	P28925	Q02362	P90493	Q6UDF2	P09310	Q6Z1Z3
P06435	Q9QJ16	P09309	Q9UTM0	O14210	P40154	Q9P7S9	Q16666
Q9R002	Q5EA95	Q9K4Q3	Q8Y0Y3	Q6NRM0	Q6XDT6	Q6XDT4	Q9VG73
Q6DD04	Q6GL57	Q9FH37	P52670	P20839	P13772	P96631	P0CF49
O81313	P0CE57	P26798	P13902	Q2UTQ9	P0CO17	Q6BGY8	Q9VDY1
A5E0W5	Q6ZPV2	Q872I5	Q4PGL2	P0CF60	P0CF66	P0CF79	P03835
P19769	A6H7J1	Q96T92	P75679	P39213	Q47718	P76102	P71956
P69342	P46337	P24350	P43610	P14316	Q90643	Q14653	Q15306
Q92985	P70434	Q02556	Q61179	P81067	P78413	Q9JKQ4	P78412
Q9KTY3	P53409	P15025	P16944	Q08773	Q2M1V0	Q90683	P33417
A4H7G5	Q60976	Q9FUY6	P18289	P13468	Q68DE3	Q8GGL1	Q8UVQ4
Q941I2	Q9FJV5	O61016	Q8IS98	A1C9M5	P50844	P76268	P37728
Q5U263	P41249	O65155	B0X9H6	P60319	Q14807	Q2VIQ3	P19653

O60870	P42968	O65154	P52604	Q52279	P13967	O14901	Q9Y4X4
Q19A41	Q9UIH9	Q5JT82	Q13351	O43474	Q13887	O75840	O95600
O53838	O04134	P48000	Q84JS6	Q94LW3	Q75LX7	Q948L5	A2Y007
O65034	Q0J6N4	P56659	P13054	P22405	P07674	P22404	Q52277
P07247	Q25515	A0R4Z6	Q0S868	P0CO51	Q54MA9	Q23976	Q7F1M0
O94395	Q6CCK2	P32807	Q2H6C5	Q9HGM8	Q6C7B9	Q04437	Q64756
P08657	P03023	Q01936	Q7WTB0	O33813	Q9M0K4	Q03474	P42166
P25084	Q7XAQ6	P40796	P23913	Q1XID0	Q6XYB7	Q3SX41	Q8N3X6
Q1PFR7	P41453	Q9M644	P10151	A8MZ59	P23914	A0LUZ1	O32506
Q00958	P0C7P8	Q7XJU0	Q9XIN0	P36198	Q25132	P50481	Q9UPM6
O35652	Q68EY3	Q6R0H1	Q27395	O85057	P20154	Q11107	Q9GNL2
P34683	Q10574	P34427	P34684	Q08CM4	A1Z9E2	Q5RBN8	Q9ZN79
Q8UVR3	P42283	Q7KQZ4	Q867Z4	Q9V5M3	Q7KRI2	Q54YV4	Q550C8
Q6FPE6	Q7KUT2	A2YQ56	Q6C0B5	Q9W349	Q06260	P42704	P96652
P96582	P45265	P56901	P52691	O93774	Q9Y802	Q10135	P65649
P52689	P13894	P03073	P03070	Q51872	O07007	O87455	Q9KT84
P21308	Q9H930	Q13342	P12980	Q980W9	P03030	Q740J9	Q4L8V4
Q6P0N0	Q80WQ8	P37935	P37938	O18214	P10038	P35192	P01549
Q6VAM4	A2YQK9	Q655V4	Q84NC2	Q2QW55	Q5K4R0	Q2V0P1	B3MB79
B3MZY6	Q16YA8	B4IB36	Q96JY0	A7Z017	P53338	P18811	P96158
Q93K57	Q56201	P0A4T1	Q7N976	A6QQ70	P78926	P0ACH6	P27245
O59851	Q02991	Q9UW23	Q708A7	Q9HG12	Q707Y6	Q707Y3	Q71U11
O93958	A7ZI28	P36981	Q10116	Q6WRX9	Q6WRX8	A7MFW6	P52684
P52161	P0CL59	Q8VJR2	P0AE73	P56270	P56670	Q9S7G7	Q9XI36
Q9FZP6	Q9LTJ8	Q5XEN5	Q9Z2E2	Q9Z2E1	Q4PSK1	Q9LYB9	O95243
Q9LTJ1	Q9FJF4	Q9LME6	Q9SGH2	P13657	Q52BY4	Q95YM8	P39678
P76114	P67741	Q1LWL8	Q0VBD2	Q5AFP3	P11746	P49736	P40377
P30666	P24279	P30665	P29496	P49731	P53091	P38132	Q0V9Q6
F1QDI9	Q9VF30	Q9UXG1	P15005	Q8QHI2	Q9IAC6	P79404	Q9BDJ7
Q54S00	A6NDR6	Q04719	O10368	Q02574	Q00566	Q15648	Q9FT73
Q5XVF0	Q9FRI0	Q02080	O55087	P40791	O13606	O00470	O14770
A2SXS5	P50221	P50222	Q9DGW5	Q51773	P0A183	P22874	P30346

Q9BRJ9	Q0VG99	P40573	Q03081	P32389	A7MXB2	P0A9G0	P45349
P52003	P37474	O51568	P57381	P30958	Q9ZJ57	P64327	O52236
Q55750	Q9UT23	Q9LW85	P95037	Q6FK91	Q8MYF0	O14354	Q4P4Y2
P32787	P53050	Q8IWI9	Q6TDU1	Q6CPW2	A5E7M8	A3LZM4	A6ZXD3
Q2G0B1	P77569	O31672	Q7T105	P35201	Q9Y7G2	P52288	P27705
P39943	Q96T58	O88368	P21711	Q9H2W2	P28482	Q8BIA3	P50456
Q32KY3	P40631	Q03164	Q8NEZ4	P33358	Q9HAP2	Q9UH92	P28809
Q32L19	P53102	P41218	P13669	P67711	O08789	Q9QZW9	A1KNB8
P07112	Q9UT46	P49309	P58497	Q92DX9	Q7JQ07	Q07608	O43065
P22915	P29904	Q75AA7	Q2URJ5	Q6BRF0	Q6CQX2	A7EFH4	A7TSV4
P0ACS1	P43161	Q46864	Q86567	Q96LU7	Q9Y2G1	P37960	P21649
Q90ZL1	A6NI15	Q84LK0	O13921	O74773	P25847	A6R7S1	O65607
Q59Y41	P0CO93	Q1ZXH0	P20585	Q4P6I8	Q6CHE5	F4JP48	O94065
P40965	F4JEP5	Q19272	O43196	O04716	Q55GU9	Q9VUM0	P52701
Q03834	Q9SMV7	O63852	P50534	Q00753	P22148	P33748	P33749
P28361	P52953	P70354	Q13330	Q9BTC8	Q9UW19	Q874N0	Q08398
Q9UW22	Q874N1	Q9HDS5	Q707Y8	P71039	Q96E29	Q99551	Q14872
Q01214	Q9Y829	Q9US51	Q9Y483	Q870I4	P96574	P0AF11	P83733
P23737	P34877	P15840	P34496	A8APW0	O68583	Q4QK86	P43640
Q5W9E7	Q8GT06	O60682	Q9M8K6	O51125	Q3ABU1	C4XS37	C1A4A9
B1MXB4	B2V693	P73625	Q9X105	Q5UQU6	Q0SM54	Q04RT2	Q4FV41
P14981	Q9QPN4	P50541	P0A2S7	Q9FNV8	Q2V9B0	P81392	O22264
P20024	O13493	P80074	Q8IEF6	P80073	Q8GWP0	P20027	Q9FDW1
Q9LX82	Q9SZP1	Q7XBH4	Q38850	Q9LTF7	Q8LPH6	Q9ZTC3	Q9S7L2
P52550	P34127	O15816	P48972	Q54TN2	P10290	Q54K19	P01105
Q54IF9	Q54Z40	Q54HX6	Q869R9	Q54NA6	Q55GK3	Q54FL0	P27898
P01104	P01103	P04197	Q9PSJ0	Q7Z401	Q17103	Q9W4S7	Q9P2K5
Q9NPC7	Q17295	P15172	P22816	Q86VE0	B6MUN4	Q5F3F2	P97500
Q6GN21	Q8H115	A2YMR0	Q9LPI7	Q9C932	Q0WV96	Q84TE6	Q8GY42
Q9S851	Q5PP28	Q9SK55	Q84WP6	Q9FRV4	Q7EZT1	A8MQY1	Q52QH4
Q9LR74	Q53NF7	Q7GCL7	Q5Z6B6	Q84K00	Q9FWX2	Q9C598	Q94F58
Q9FMR3	Q9FIW5	O04017	P70670	Q47005	P39667	P27278	Q9EXL7

Q7Z5D8	Q9H9S0	B7UJV9	P25293	O53856	Q3MHZ1	Q9FLJ2	Q2YDP3
Q9VJQ5	Q9WUB5	Q8QG78	Q9Y618	P0A3S0	P46581	Q62414	P22392
P25233	Q02835	Q96FI4	Q969S2	P67431	P06903	P06020	P46496
Q5ZL67	Q5NUA6	Q9Y4A8	Q91431	O88942	O77638	Q12968	Q14934
Q9WV30	Q5EAD3	Q02780	P97863	P14057	P13623	Q04861	Q63369
Q18034	Q54BK0	Q12986	P32265	Q9LXV5	Q9M9X4	Q93ZH2	Q8VY64
Q945M9	Q54S29	P23511	Q9SLG0	O23310	Q60EQ4	O04027	Q65XK1
Q84W66	Q9SIT9	Q9SFD8	Q54WV0	P25211	Q9FMV5	Q8L4B2	Q58CM8
Q557I1	Q13952	Q9MAN1	P70660	Q9Y4Z2	O17611	O16966	O45449
Q9TZ99	O16391	O17082	O16963	O01931	O17657	O17683	O17748
O17934	O18048	O02305	O18086	Q6NV18	Q6AYI4	Q03435	P0CO25
P87057	P41999	Q23294	Q21701	O02151	Q09528	Q21878	Q25604
Q09587	P41828	Q19345	Q17905	Q18192	Q21006	Q17771	Q9XTJ4
Q9N4B8	O76828	Q22555	Q17370	O45666	O45436	O17927	O17933
O16425	Q9TXJ1	Q9NA51	O62389	O44960	Q9XVV3	P91829	P41829
O16604	O02316	O18141	Q20765	Q965W2	O02235	Q9XYB7	Q9U2R6
Q23489	P52392	Q9X1T8	Q88FY0	P30667	P56269	P24426	O26703
B0R2R5	Q8TV90	C3N649	Q8R5U7	Q02441	Q07597	P19212	P28349
Q15270	Q9UD57	P50220	O95096	O15522	P97436	P78367	P70061
Q9C056	Q7X9B9	Q0JC27	Q9SVF1	Q9LE38	Q8RWY4	Q84TH9	O22864
O69711	O60393	Q8VIH1	Q00678	B1HPM0	P04682	P15940	P22537
Q5FVM4	Q2FKF1	Q01709	Q00790	Q06615	P97459	Q8IXF0	Q1ECW2
B1Q2A8	Q61937	P43130	P68950	Q89707	O10440	Q3UV55	P55055
O35627	P51179	P45448	Q64249	Q2JVG1	Q90X44	Q62792	Q03125
Q923B3	Q1W1G1	O96028	P03565	Q06661	P27476	Q9L132	P33779
O07567	Q96KG9	P29814	O72907	P10577	Q04849	P49790	Q9W0T1
Q0P569	Q9FVQ1	P08199	Q9HSL7	B3DTU8	Q8ZWG7	Q8U4R1	O60356
P39720	Q75BN0	Q6FJJ1	Q6CXS0	A7TGQ2	B3LRD9	C5DUX2	P42641
P52379	Q6UDH3	P0A4T4	P24068	P23803	Q50EX6	P41933	Q9FNY7
Q8TAK6	Q13516	Q24432	Q6XBJ3	P12959	P39768	P21957	P27872
Q95RW8	Q58DC8	Q6BSE2	Q54RM2	Q8I615	P54789	P54784	Q55CU7
Q9JK30	Q10067	P54790	Q2YDI2	Q9Y794	P54791	Q54C22	Q9ZVH3

Q9Y1B2	Q9WUJ8	O74796	P38826	Q9M1K0	Q9FVS2	Q681I0	P72171
Q9N6K2	Q9SX99	Q8GXH3	Q8GWJ4	P56672	Q5XKR4	O76971	P80205
Q26417	Q9BRP0	P51521	O87883	Q89769	Q65245	Q04073	O43422
Q00366	Q64662	B3DM25	Q9H3D4	P28813	Q753Y2	Q873Y3	Q6FV94
P0CS63	Q6BSZ4	Q6CQ07	Q8J257	Q6H8R9	P78978	P33400	P10590
A8XSQ8	Q86YC2	Q3U0P1	P91943	Q9SX27	Q8NDF8	Q68ED3	Q5XG87
Q77MR9	P10226	Q6UDI9	P36702	P22996	Q9RZE7	Q9PHE9	O51395
P07621	Q9ZK75	P0A5R3	O83295	O51066	O68891	P75352	P73077
Q59189	O54479	F1MF21	Q9ZP54	Q08824	Q9UGN5	Q9SWB4	P19071
Q9HBE1	P47236	P15863	P09084	Q02962	O43316	P47237	P26630
P23759	Q9PUK5	P47242	Q86U86	Q6IR52	P40426	P0A4T6	Q52154
P57721	Q0VCU0	O23875	A2YXQ1	A2WM14	Q8LT07	A2XMN1	A2WV68
P39784	P35227	P40883	Q9YFT8	Q8ZTY0	Q4JAI6	Q9Y9V7	Q00265
Q8ZYL6	Q5JFD3	P57766	Q9YEZ5	P24314	A0RXH7	B9LU30	Q8TWK3
Q7T6Y0	Q74MV1	O10308	P61074	Q9HJQ0	P52679	Q09926	O60035
Q2YDC9	P0ACM1	P31368	P31369	Q24705	P12383	P33200	Q06149
Q8NS92	P23801	P32512	P42195	Q86TG7	Q7TN75	Q7M3K2	P13975
P41073	P43459	P0C961	P0C962	Q5HN74	P31078	Q97QZ2	Q96BD5
P06184	Q17381	Q64028	Q8IXK0	Q8NDX5	P52698	P36093	O80805
Q7RTV0	P14309	Q04621	P16684	P07269	P07270	Q05534	Q57QC3
P39769	Q04449	P34205	P27526	Q28811	P12768	Q55081	P61496
Q8N3S3	Q9V9A8	Q10328	Q99453	O75925	Q6AZ28	Q8N2W9	Q94131
Q80SX8	Q9UUA2	A6ZM04	O80536	Q570R7	P10030	Q8L5W8	A6QPY8
P79149	P77170	P52960	P10036	O93385	Q99697	O18400	P20665
Q96680	Q64749	Q8BG99	O34381	Q5YGP8	P58195	Q9N4H4	Q60953
P52168	P51022	P51023	Q28466	P31503	P09086	P20264	Q29087
Q63934	O97552	Q90270	P31367	P78424	Q05587	Q04088	Q9P215
P10266	P63135	Q01842	P38485	P03599	P36341	Q6XKE6	Q9Y253
Q9UNA4	Q9UBT6	O74944	P94544	P19560	P25059	Q89703	P11204
P03362	P04586	P18096	P12894	P11283	P03354	O92815	A8WWH5
P27536	A9JTY4	O13988	P62597	P31370	P23757	P23758	O13852
Q96QC0	P37233	O18971	P07272	Q52620	O22785	Q3UTQ7	Q9H4Q4

Q9GZV8	P57071	Q9HAZ2	O23277	Q13029	Q80V63	Q9NQX1	Q9NQV8
P03857	P0A0C5	Q5HJZ6	P10025	Q9HCU5	P13925	P22262	Q9GLW0
Q8AYI2	Q28590	P48785	P48786	O02334	Q55BM5	Q9VPH2	P49643
P20457	Q45032	P57220	Q9PLE5	Q9ZKE4	P0A5A6	P05445	Q1RI08
Q9WY22	O83258	A3N1H2	P67674	P49412	A9LZQ2	Q82XQ7	P10277
Q8FDB5	P19782	P15342	Q64256	Q3LU38	P29617	Q9U6A1	Q92786
P11826	Q9IZT2	Q7KWK5	Q5Z8Q9	O14283	Q90963	Q99811	P62089
Q06552	Q06553	B0Y6K1	A7BJS7	P08433	P17502	P0C230	Q09838
P35820	Q5XXA9	P37344	Q7RTS3	O04682	Q9LZS0	P35233	P40193
P72131	O32138	Q3UEB3	Q00577	Q8R4E6	P37551	A7MFD3	Q9SKZ1
P09546	Q44333	P0A9U6	O42173	P16146	P11638	P0A0N3	P56260
P10563	P11637	Q96B01	Q9SW63	Q9SK02	O15315	O35719	Q54PJ7
Q9LQQ2	Q1ZXF0	O55230	Q9DG67	Q9KC68	Q65DU9	Q5KVB2	P76062
P53709	O59753	P28519	P36617	P48581	Q75EN0	Q5A4N5	Q6BLM3
Q9QXK2	P33288	O74747	Q6CHI1	P06777	Q00578	Q5T890	P40352
P14736	P36601	B3MMA5	A4PBL4	P32863	Q4WVM1	P0CQ67	Q6BIP2
Q7S1P9	P36607	Q4PGG5	Q6C2R8	O58001	Q5JET4	P74391	B0R636
P43465	Q90523	Q91187	O54452	Q39204	Q96TL7	P11938	Q8LC30
P42736	Q8H1E4	Q7G1L2	Q7VKV0	P18515	P03050	Q91392	A2T928
Q9C6P5	Q9C688	Q9FG68	Q9M2Y9	P73123	Q00312	Q24472	Q08999
Q92804	Q9K6K2	P07760	P06400	Q01473	P18754	Q13YL3	Q2RNI6
P03036	Q37907	P18679	P09964	P20098	P69409	Q47588	Q09772
Q9LUJ3	Q8NG50	O13862	Q4I0C5	P25611	P19541	Q05950	Q9P6H9
P40379	Q9ZUP2	Q3EAS6	Q39199	Q75JL2	Q08784	Q02347	P0A5U5
Q9F407	Q59592	O86382	Q54428	P65982	B7GUX7	P49997	A0ZZA1
Q3A8P5	B0T360	Q5L648	Q48AS5	Q11NR3	C1CW06	B0U178	Q2SQZ4
A0L3I9	Q1DFP6	Q3JF36	B1ZSD3	Q3A8M6	A4SC23	Q4UNG8	Q2S6G1
C5BKM1	O83049	Q83NZ4	Q8PRG0	O51528	Q8XD86	Q9ZJA1	O69460
O50224	O32044	Q5SJ47	P46063	P73421	P50729	P33228	P39792
P14199	Q9V2M0	Q9V2K6	Q9V2D7	O58782	Q9V0Y9	Q9UY16	Q01751
P13870	P68922	Q9T1U3	Q02582	P0C079	Q01201	O50461	O33347
P65067	P64528	Q04864	P0DH85	P0CAP4	P0CAP5	O23076	Q8RYD1

Q9XIB5	Q84J39	Q8LAV5	Q9M8K2	Q9LXE1	Q9SB79	Q9SB80	O81782
Q8H2D1	Q8S8E8	F4IQL7	F4KFT6	O54003	Q52221	P18921	P20044
P03568	Q00338	Q934T6	P31617	P05683	P03856	P60470	Q9BWE0
Q9RMZ4	P15419	O80302	P03660	Q38617	P18016	Q9IG45	Q80GM6
Q47274	P76161	P33520	P41412	A3EWL3	Q4KWZ7	O94623	P12689
Q9RVT3	Q7UW59	Q9WX14	Q8VUP4	O97472	Q23696	Q8VEE4	Q92372
Q9ZQ19	Q8LFJ8	P0AFW1	P46358	P35600	P35251	O60182	P38630
P38629	O94449	O94697	P34348	P48743	A0JMF8	A2BGA0	Q9JL61
Q2KHR2	D3YU81	O00287	Q9Z205	O32237	Q00453	Q750N3	Q6FLP2
C5DGQ7	A7TE38	B3LR49	C5E4G1	Q9YC75	Q97ZF5	Q58907	O58530
A7FN79	Q6DA22	P0C7M4	P81128	O81242	P54292	P77732	Q63630
Q10445	P87235	Q9Z3Q6	Q8NHV9	P32445	Q03182	Q6NI56	Q8FQS2
Q9FGD1	Q9LVU8	O81791	E0TZF9	Q12224	P32338	Q10160	Q02685
Q5ZM20	P03049	Q9LBW6	P04487	Q8N5U6	Q9QZS2	Q9HSF6	F9VN79
Q99020	P0ACI1	A2ZAI7	Q7Y0V7	P38022	B1JS04	O34857	Q0J235
P51448	Q92753	P51449	Q9SJQ6	P10181	P25042	P62182	P03048
P06227	P62177	P24219	P38944	Q01194	O83149	P04524	P30333
P52434	Q92399	P20436	Q80TE0	P17546	P11414	P28364	Q6GZW7
A5DCV3	P14248	P08707	P16117	Q37906	P51704	P18680	P06019
P03041	P03034	P21678	P03043	P11189	P17890	P15238	P04132
P08979	P39956	P23250	P0ACS8	P52323	Q9Z7F0	Q9ZMY3	P0A603
P17531	Q2K619	P43344	P74565	O68887	P37970	Q9KI19	O51804
P06574	P19433	P66810	P10726	P19940	P02964	Q06198	P12254
P26768	Q3UH06	P33395	P38766	P40992	Q9S7B2	P38781	Q06639
Q9P7W8	P46974	P39650	P78871	O34419	O85264	Q9P7D9	Q05543
Q2UMB1	Q6CQ86	A5E4Y7	Q6CHJ8	Q8TUS2	P38035	A8WS58	B4L1Z2
A2AQ19	Q9UUI6	P32607	P38165	E0TY12	P40962	Q6PF39	Q9XSB7
Q08775	Q9Z2J9	Q8X4Z7	P70827	Q5L6Z3	C0QKP5	B2KCD9	Q8REJ7
Q04RG9	Q2SS60	Q7NAN2	Q4A7W5	P75243	B2A5L5	Q2GDJ1	A9BIJ5
A2C1Y5	A9B9X1	Q7V7T9	Q7USB0	O83554	B1H0B1	B1AJ90	Q9PVY0
P51128	Q5TJF7	Q9W2Q1	O97039	Q7SYB3	Q5EG55	Q7XLX6	P42554
Q9FMF4	Q80YR5	P55109	P48383	Q9Y467	Q8BX22	P39806	P40847

Q2FJ20	P82979	Q2FEJ8	P0C0R2	Q2G1T7	Q2FVY9	Q2FIX1	Q2FEB2
P10571	Q60611	Q0K846	P19601	P30052	Q25214	O14335	P37076
P74892	P24508	Q9BWW7	P15859	Q64124	P24349	P34706	O32242
Q8JJ26	Q9HB31	P70368	P87164	P21344	O43058	Q65U90	A1STA5
Q09415	Q9Y6X0	Q9BYW2	Q53H47	O14229	Q9UST7	O74458	O60174
P20134	P32432	P23246	A7ZHN7	C0SP86	P56644	P39361	Q17241
O60902	O35750	O15266	Q91848	O22056	O24621	Q9ZSL6	Q9ZNX9
O31654	A1TEV0	O07582	O53590	A7X3L7	O05404	Q45585	P35165
O05409	P81133	Q14190	Q24167	P22753	P37459	P46954	O82802
P11978	Q8Z4A6	Q9AWB2	P25294	Q62233	Q9UIU6	Q8N196	O93307
P35207	P34707	P38889	Q02100	Q4QLV2	P22807	P32030	P32031
Q6D5V7	P45897	Q9I8V2	Q1HE26	O43541	P38935	Q29L50	Q9MA17
P51531	Q969G3	Q56A18	O66878	P51834	Q8KBS6	B9E1H0	Q81ZL2
Q8REH4	B8CW13	Q1INB1	Q88WJ9	P47540	P41508	Q10970	Q8TZY2
Q6N1B7	P73340	C4ZJU1	Q9HK21	P32566	Q26600	Q26601	Q26602
Q26604	Q26605	P38128	Q08142	P76053	E1B7X9	G5EDG2	Q9VL72
P30340	Q6P5C5	Q9YGN6	Q8NB12	Q3KNW1	P08044	Q8K0S9	Q5E9M5
Q8BP86	P25357	Q9VQS7	P53438	Q5JUK2	Q6IUP1	Q9NX45	Q9D489
Q9QX47	P62559	P37078	Q8R2Y9	Q9VM17	P35716	Q8AXQ4	Q9UN79
P40657	O60248	Q62247	Q61473	Q9W7R5	Q8CGW4	P41225	Q06945
Q7YRJ7	Q51506	P0A9E4	Q27350	P52933	P58253	P52937	Q9N1Q6
Q9N1Q5	O35892	Q9HB58	Q3KRF1	Q01714	Q5E9U0	Q9HAJ7	P15281
Q6BEB4	Q3SY56	Q5XGT8	Q86W54	Q700C7	O95238	P80304	P17433
Q9Y657	Q75GR5	Q8S9L0	Q653Z5	Q6Z461	Q7EXZ2	A2YX04	Q0J0K1
Q9LGU7	Q9S840	Q0JGI1	Q6H509	Q0E3F8	Q94JW8	Q75LH6	Q8S9G8
Q8GXL3	Q01KM7	Q700W2	Q6I576	Q9M4A2	Q22236	Q8SVS9	Q9Y5K1
P23179	Q9M4A1	Q17768	O17582	P06843	O13472	Q9FUA4	P24417
Q6AXY9	O97676	Q6ZRS2	P09007	Q92259	Q54TY7	Q54RY6	Q55F37
Q90718	Q24535	P11831	P15082	Q9R9U0	P52172	Q52KI8	Q10213
P07665	P15619	P07664	Q03256	Q05738	P36394	P36395	Q83NU2
Q8KAM2	Q8ZSD2	Q82CI4	P66857	Q84J78	Q9CYR0	P60471	P28042
O66475	O51141	Q38504	Q823K0	P59927	Q9RY51	Q890K1	Q72UU3

P75542	Q98PV9	Q8EA81	Q8CNK0	O83101	Q9PPT7	Q9NEL2	P37549
P29840	O14470	Q05153	Q293F6	P11020	Q9TUZ1	Q61AP6	Q9WVL2
Q6DV79	P42226	O00910	Q20977	Q54BD4	Q7QDU4	Q24151	P50104
P26047	P40798	Q870J1	P36631	Q08400	P13574	P39015	Q9LMK5
Q0E7J7	Q6ZVD7	P17305	B3LFK1	P38704	Q7M2P1	O97965	Q09821
P32579	P54000	P33181	P08970	P28159	Q9Y1T5	P31266	Q00492
Q38895	O75683	P20193	Q9C5P1	P25172	Q6NVH7	Q8VCI7	Q06182
Q8W475	Q84JG2	Q9XI07	Q8VY05	P25302	P08153	P40418	P09959
Q6FK48	P0CO19	Q7S133	O13682	Q6CA87	Q60563	Q03410	Q9BX26
F4IHS2	P55733	Q08812	Q00840	Q05309	O35003	P10486	Q47281
Q60295	P08956	P17224	P75431	P10485	Q57594	P19704	P75604
P75279	P75416	P06991	P75435	P44152	Q60296	P71344	P17222
P75487	P75488	P59697	P12249	P14506	Q54KJ8	Q9D902	P79011
Q9SU25	Q54WV4	Q05913	O94416	Q54KT7	P41900	O94424	P41896
P71366	P08763	P12364	P40814	P11901	Q55566	Q45620	P16942
Q00461	Q00462	P24607	P35878	Q5ZJF3	Q7KSD8	Q9ATB4	Q8I8V0
Q5ZLN5	P97357	A8XLS0	F1R3W0	B8AX23	F7BLM1	Q15572	Q9D4V4
P51123	Q80UV9	Q99142	Q92750	Q90YI8	O73823	Q16559	Q8T0D4
O16867	P03409	P79005	Q93WI2	P52653	Q9LL45	P26356	A7UFC2
Q5UQP9	P26354	P32085	O74045	Q27850	P13393	Q9NL40	Q16650
O75333	Q20257	P90971	O02073	O43435	P70323	Q9UMR3	Q9UL17
Q9N2K7	Q9TZL0	O61764	O45291	Q09376	Q23622	Q9U2C9	Q9NA56
O15119	Q22813	Q861Q9	P79779	E1BEA8	Q8JIS6	A1YF56	Q66JL1
Q22292	P03934	P34257	Q9F4G3	Q2KI09	Q9EPQ8	Q7RTU0	Q8T2A4
Q9UL49	Q9HEQ9	P39885	O82277	A0AQW4	Q9S7W5	Q93Z00	Q9C9L2
Q9LEZ9	A1YKT1	Q9LT89	Q9FYG7	Q9C7G4	Q93V43	Q8LPR5	Q94045
P53999	Q872F4	P87294	Q9FME3	Q9C518	O64647	A5F384	O07776
P11866	O87940	Q94818	Q94819	Q15562	P29549	P29548	Q06183
P18412	P97516	P07387	P53038	Q9D7K2	P54274	Q9PU53	O35144
P11455	Q9SPU7	O14746	Q8LKW0	O13339	O77448	Q06163	Q8NFU7
P28816	P03039	Q5ZAY0	Q9UNN4	Q8IUE0	Q9UTL5	P03001	P39933
Q9Y5Q9	Q9UKN8	Q54GS8	Q8R2T8	P98152	Q9NQB0	Q924A0	Q0II87

Q8WVM0	P32367	P34111	P33339	Q12415	Q12308	Q22703	Q14188
Q46M57	P42427	P19532	P19484	Q9YAD5	O29501	A8M8Z5	A0RYF4
A8ABJ9	Q8TY11	A2SQM6	Q2NEV4	Q8ZYH0	Q8U3H5	A3DN07	C3MZJ0
P49373	P07273	O27001	P14233	Q15583	P70284	Q9GZN2	O43952
Q39117	Q9SDW0	B0R9H2	Q5NVM3	Q98867	B5XCB8	Q9H0W7	Q0P5B4
Q5ZHN5	Q0IHI7	Q8TBB0	Q9BT49	Q9H5L6	Q9PUA8	Q9W6I9	O97716
P18119	P10828	Q28571	P43462	P38141	O94278	Q96FV9	P59924
Q4W5G0	Q6B0B8	Q8BUZ3	G3MY25	Q6NT04	Q39127	P22711	Q9U3V5
Q805B4	P47974	P23949	P97273	P14083	P18102	P43345	P82094
Q641M3	Q4V7N7	Q46731	P21580	P08504	Q45119	Q06126	P20189
P04130	Q45666	P13988	P13989	P13991	P05845	P34219	Q9LVG2
Q7T6X9	Q969P6	Q5UQB5	Q9PHK2	O66893	P30181	O28469	P40114
O51768	P93119	Q54RC3	P30189	Q9ZMV7	Q59046	Q9JN65	O73954
P07799	Q9X909	Q9HM08	P41511	P41512	Q9PEV8	Q9X3X7	O42131
P90520	Q00942	P27570	Q9QSK1	Q9NG98	O70157	O96651	A0R979
Q8T2T7	P14294	P13099	P07065	Q8TQF8	A2XFC1	Q9YE64	B0R4D3
Q58434	B8BDQ4	Q8U0K8	Q92547	P09176	P23992	Q2EES9	P53147
O94842	O74205	P15795	O94900	Q8N7U7	O14246	P15533	P35883
Q04222	Q45968	P08770	P60231	P03009	Q51761	Q54513	P35884
P80011	P18416	P37247	P35881	Q45618	P37248	P59800	Q6ZDF3
Q00184	P14565	P17909	P33785	P10026	P33788	P0CK38	Q06924
P55407	Q46087	P06627	P12059	P07636	P35879	O05069	Q8VWK4
Q8BXJ2	P39796	P36673	P05476	P07676	P02788	Q27896	Q24119
Q6E2N3	Q8L7L8	Q9FFY9	Q9M347	B6EGC9	Q822W7	B0B9S0	C6DF27
P58700	Q9UHF7	Q24742	Q8GV05	B3DJM5	P22265	Q9P7V3	Q99598
Q9FJA2	Q9FT81	Q8XBK9	Q15361	Q9UNY4	Q93PU7	P42282	P17789
P70785	A7FHW9	O96642	Q11094	Q9GNV2	Q8MIH8	P42681	P33122
P44694	P25490	Q6AUG0	P49413	P25976	P0CB48	Q9NZI7	Q4G0F8
Q23946	P83949	P26370	P0AGA8	P0ACT8	A7ZV62	Q8GZA8	P39001
Q93705	P29506	Q50D79	O77215	A8WL06	Q9N5D6	P13528	Q19203
O22768	P21968	Q86B79	Q28ZA9	P40349	Q04411	P32326	Q15853
Q9VPQ6	P20153	Q12132	P18957	Q9Z985	Q46577	P29927	P28009

P25735	Q08518	Q9CD72	P53528	P75437	Q9ZD95	P66798	P44487
P23541	Q623D4	Q93899	Q8W4L5	Q47744	Q57475	Q57534	Q57120
A0QRY6	Q46560	P21681	Q46561	P17255	Q8TUT0	Q2NKI2	P03698
P07695	P11107	P12551	Q9PTN2	P06455	P36720	Q02513	P08345
P36784	P27552	P27223	P36798	P06931	Q8BDG7	Q89808	P03127
P36806	P50775	P36808	P27555	P30734	P30735	P22159	P06933
Q705F5	P03130	P27556	P03131	P87377	O95231	Q9W769	Q14119
P03006	P14815	P17311	P14816	P13342	Q05286	P06023	O05070
P07928	Q26366	O21945	P20313	P68742	P40522	P40041	Q9C2N1
P19893	P24647	P32511	Q9FIE3	P0A247	P08062	P0A2T2	P62722
P37398	Q2YJ50	P08342	Q8BDG3	Q80905	P27558	P27235	Q80932
P22856	P23848	Q04580	P22808	Q9SLB9	Q9T1U6	P17637	P30020
Q77MR5	P06492	P09265	P17638	P24849	P03098	P04011	Q3L6L8
P20224	P36322	P16790	P52439	P03763	P96343	P06022	P71388
P25476	P08557	P71389	P08558	Q9I1P2	P03690	P03691	P22562
P03688	P14327	P22625	Q00572	Q02123	Q01687	P16654	O42477
Q9T1Q8	P20710	O22002	Q05244	P04889	P05998	O22010	P75970
Q03631	Q54F46	P41846	Q01371	O75717	Q84LH3	P71592	Q6MX01
B2GH83	Q8G5J9	O05847	Q8G5K1	Q7TWJ2	O69649	P96215	A0QTT1
Q7AKN0	Q7AKI9	B2LXS7	Q9LL85	Q9XI33	Q5AP80	Q5ANB1	A3B6V0
A3BKM2	Q9LM84	Q7XM13	Q6X7K0	A2XZR3	Q6S3I3	Q9SIB4	A2ZH47
Q8H1D2	A2WWU7	Q9ZVF5	A2XG77	Q0JKK6	Q6X7J5	Q5QMM3	Q6X7J4
Q6X5Y6	P40151	Q9LG05	Q9SV15	Q9SVB7	Q9SA80	O22176	Q9FL92
Q9SZ67	Q93WV0	O04609	O22900	Q9C5T3	Q9FLX8	Q9SUS1	Q9FL62
P59583	Q8S8P5	O65590	Q9CAR4	Q9SR07	Q8GY11	Q9ZUU0	Q9S763
Q9ZSI7	Q9FGZ4	Q9FHR7	Q8VWQ5	Q9FH83	Q9SUP6	Q93WU8	Q9SHB5
Q9C983	Q93WU7	Q9SJ09	Q8VWV6	Q9LP56	Q93WV7	Q93WV6	Q93WV5
Q9LXG8	Q9FYA2	Q9SI37	Q554C5	Q9FG77	Q9XI90	Q9C519	Q9STX0
Q9C9F0	Q19546	O93530	P50902	P49953	P36219	Q9SB92	Q8LL11
O35426	P40489	Q8XTL6	O26979	B3DQV1	B3QM22	C4ZGY6	Q8KRZ2
C3PLU8	Q8KUV2	A8F7B4	Q8RA66	Q9PQS0	Q9CG32	Q5M1I1	Q8U9U6
O31490	P22876	P39781	A1C7P9	P28518	Q24595	Q01831	Q4JC68

Q9LKI5	Q54PN5	Q24087	Q682D3	Q9FKM5	Q9CXE6	Q54LY5	P13010
P23789	P97789	P16557	Q44406	P21940	P06519	P27159	Q05092
O53626	Q60328	P67705	P67737	Q8NYT6	Q54UV8	Q60386	Q6GKL1
P44978	P47347	Q9SX41	Q7XFU9	P45008	Q5N6V0	F4I8U3	Q7XGM6
Q8CSL5	Q58520	Q53N90	Q2NF79	Q53QI0	Q2R9D2	Q92GC8	Q9ZU26
Q2QMT7	Q2QMT2	Q8S2E6	Q5JNA1	Q11063	C4KH08	O27346	P67159
Q58721	Q0JP99	P67434	Q9HIH6	P0A0Z0	P68913	Q58793	Q83BT6
P44540	P45215	Q9XIB4	P44207	Q74NA9	P72979	A6USD6	Q58940
P67436	Q58958	Q5RM09	Q9MA32	O28646	Q8ZWT6	Q59029	Q9FX77
O05086	A0RYF8	Q9SYL8	O28487	P67438	P67669	P67671	P44558
P26833	Q97MK5	O53647	P67665	A3A463	P62087	O33060	Q9SIC7
Q9SIC3	Q5JHH2	Q7A3W9	Q8RV83	Q6DSS2	Q9SJ07	Q8IRH5	P44580
Q1G3B8	P67667	Q57682	P37371	O23659	Q9KPI4	Q9SJ98	Q6K3B1
O66625	Q9XIP5	Q7JUR5	Q6EU30	Q10809	P67747	P67440	Q6K5K2
Q01856	P0A648	Q10Q26	Q851V1	Q10GP0	Q851W4	Q851V5	Q10GM4
P67442	P75438	Q9SZ05	Q60275	Q57807	Q1PES7	Q57809	B7EIH2
O53712	P44675	Q3V4U8	Q10QS9	Q84R27	Q54B29	Q87K77	Q3V4S9
Q05954	B3H469	Q9SY12	Q9ZR15	O33682	Q9SB61	Q7XS74	Q3V4R9
Q04310	O53750	O53757	O53759	Q7F9W2	Q7XLP4	O05023	P67739
P55360	P55373	P55389	P55409	P55449	P55559	P55565	P55576
P55613	P55615	P55626	P55629	P55643	P50337	P55658	Q53198
P55729	Q8VKK5	Q8DET1	Q9LST3	Q5JF28	Q3E7N5	Q680D9	Q6L4H4
Q9FNI3	Q3E922	Q57988	F4KIX1	Q9FHH1	Q5KQI4	Q9FFX1	Q58006
O66861	Q5VS55	Q9LHY9	O86029	Q67VL7	Q9RMY4	Q9Z9W1	P44030
Q8T053	Q057E0	Q55963	Q84YL3	Q5UP03	Q58161	Q0D5G4	Q9ZCH6
P67432	Q58184	P44876	Q9YDZ1	Q8H507	Q8H506	A3BH91	Q8EUW6
Q6Z0D2	Q6YTQ4	Q6Z0D9	Q55433	P81315	Q10550	P44923	Q1ZXA9
Q58352	Q58371	P12552	O13527	Q9XFB1	Q9LDT3	Q8GW46	Q76EJ0
Q47156	Q47149	P21514	P77700	P23823	P23822	Q10086	G2TRN9
P19880	P24813	P38749	P40917	Q03935	Q08182	Q06596	P46117
Q10186	O31417	P0A9T7	P32788	P77634	O31432	P77746	P30979
O31449	P39901	O31456	O31459	P0ACU0	P75811	Q10332	C0H3S9

P42239	P70955	O34464	P51342	Q1XDE4	P49536	P51237	O94720
P94387	P43461	P76034	P77615	P94423	P94433	O59830	Q08792
P25279	P96625	P77171	P77626	P67700	P96660	P96662	P96663
P96673	P96676	P96683	P0ACM3	P96690	P96701	P96705	P96708
P0ACR3	O05494	P77402	P77721	P39961	P76241	P76369	O35034
P0ACM7	P0ACR6	P0A9F0	P25992	O31517	O31522	O31541	O13706
P45785	O31497	Q7WY76	O14130	P0ACR7	Q48660	P37767	P33634
O31560	O31564	P52133	O06474	P53243	Q99315	O60056	P77295
P58664	Q46802	P52044	Q9Y7K9	O13535	O60130	O60131	P67661
O31592	P54590	Q9P6I9	P32398	P30791	P37640	P37641	Q796S4
Q04013	Q04116	Q9HE16	G2TRT1	P37671	P37682	P96194	P31449
A8ACM1	P31475	P0ACN0	P32144	P32677	P10427	P32425	P40331
Q9P7H9	C0H3Y4	P47024	O74915	P16694	P0ACU8	P39334	P39360
P39376	P39389	P39399	P0ADD8	P77601	P77379	Q95QY7	Q9HDX1
P34303	O34827	O31679	O31690	Q12244	Q5UPI1	Q11101	Q05854
Q9UTA7	Q9UTA1	O07627	O33812	O94392	P34447	P14707	P34454
O31761	Q03615	O59741	O59744	P53749	P34536	Q8X7R5	P77309
P77559	P45968	P42547	P34670	O34701	O34647	O34901	O34892
O34844	O35009	O74308	O32006	O34791	O34336	O34766	O34449
P40923	P34161	P34664	O31834	Q8FFE0	P77396	Q8ILR9	P54182
O07127	P50831	Q09452	P45902	P45904	Q46942	Q46855	P54529
Q5UPI0	Q5URB4	Q5UQS2	Q5UQA4	Q5UQQ9	Q755R9	B3LQP5	O07906
Q12172	Q8WTZ3	P14307	P97247	Q10127	O34533	O32071	P0ACN2
O35038	O34712	O34970	P70790	Q9JMT8	O05236	O05261	O32181
C0H3Q8	O32228	O32238	O34709	O06978	O06987	O07001	O07019
P40762	O34692	O32235	P25150	P39601	P70993	P71036	P94578
O05217	Q10938	C0H3S6	P42103	P46330	P55181	P37510	P37503
P37486	Q45591	O66401	Q86YH2	Q6N043	P0CG31	Q96PM9	Q96KM6
B2RRE4	Q6GNP2	Q4V348	A8MWA4	P47043	Q0IH98	O96006	D2EAC2
Q96DT7	Q9WTY8	O95625	Q9Y330	Q05516	Q90625	Q13105	Q9HC78
Q5TJE2	Q80X44	A1YEX3	Q9HCK0	Q5TC79	Q5EXX3	O15060	Q9NUA8
B2RXF5	O43298	Q8NCP5	Q8R0A2	Q9UFB7	P10074	Q6ZSB9	O95365

Q8NAP8	Q9Y2K1	Q8N680	Q9H5J0	O15062	Q0V8G8	Q96C00	Q84JE8
P09090	Q24648	P15822	P31629	Q24762	Q6PGE4	Q9NPA5	Q9NTW7
P28166	P28167	Q9C0A1	Q61329	Q42430	Q8NHY6	P10078	Q8N8Y5
Q9NU63	Q8C6P8	Q62396	Q6PCR6	P80944	P18712	P18715	P18723
P0C6A0	Q1WG82	Q7PYU6	Q8N5A5	A1YF22	Q80VX4	Q9H4I2	Q55940
Q61467	Q96T25	Q9NZV7	Q10424	Q6CR69	A7TPF8	A6ZTU2	Q63HK3
Q9P0L1	Q5H9K5	Q3V0C1	Q28EG9	Q0VD35	Q15326	Q8NC26	P38621
P52741	P52743	P52744	P52746	A0MS83	Q15697	O14628	O14709
O95201	Q5R8K4	Q9UDV6	Q9UL58	Q9P2Y4	Q9UL36	Q1L8W0	Q16600
P15620	Q96GC6	Q9NSD4	Q8N554	Q9Y2X9	Q9UDV7	Q9EQB9	O60281
Q9NR11	Q5JNZ3	Q9P2F9	Q8BZ89	Q5BKZ1	A2RV70	Q96JL9	Q9H4Z2
Q9BYN7	Q6P1L6	Q9GZX5	Q9NW07	Q8N895	Q7RTV3	Q9EQJ4	Q9UJN7
Q8TD17	Q9C0G0	Q9H9D4	Q86VK4	Q96IQ9	Q09FC8	A1L1R6	A1Z9R4
Q7Z4V0	Q8N0Y2	P59923	Q9NWS9	Q9Y4E5	Q96JM2	Q7Z7K2	Q96JG9
B1APH4	Q96MN9	Q9ULM2	Q96IT1	O60304	Q8TCN5	Q7ZZ00	Q8NB15
A4FV61	Q8N8E2	Q92618	Q96C55	A6QR00	Q9HCE3	Q6NXK2	Q8K083
Q96MR9	Q32KN0	Q8BY46	Q86XF7	Q9H609	Q9BSK1	Q8NAF0	Q9UK33
Q86UQ0	Q92610	O00488	Q96LX8	Q8N2I2	Q6ZSS3	Q96I27	Q5EBL2
Q14966	A5PK30	Q96N77	Q9H582	Q5T619	Q96CK0	Q8IZM8	Q8N720
Q8NDT4	Q2TA17	Q96BR6	Q8IZ20	P0C7X2	Q96CS4	Q5VV52	Q3TDE8
Q9H7X3	Q569E7	Q96C28	Q3U288	Q8NE65	Q3U133	Q96N20	P51815
Q8R0T2	Q8BIQ8	Q7L3S4	Q8BI73	Q8N8C0	Q6ZMS7	Q8NCA9	Q8N393
Q6ZN11	Q2TB10	Q6ZRF7	Q6PD05	Q17R98	Q9Y2P0	Q96EG3	Q08AG5
P0CJ78	Q810A1	Q9ERU3	P17041	Q96MX3	Q15929	Q16587	P51502
P17098	Q05481	A8MXY4	P0ACS6	P18739	P18745	P18748	P18743
P18751	P18752	P18853	F8DT24	Q3URR7	A1YEP8	Q8TBC5	P17040
Q6NSZ9	Q8IWY8	Q9NX65	Q80VJ6	Q8NAM6	P34450	Q9Y7I8	P54479
O05839							

5321 non-binding proteins							
B4IBI9	Q86YV6	Q9UUG3	O31959	P27096	Q09931	A3PSL5	P14534
O29714	B1HNY1	Q92338	Q4JBF2	P50110	Q5CKJ0	O31983	Q6PL45

O32037	O42895	O44662	Q12019	Q2V3B2	P46666	Q9LD55	O53475
Q9NZY2	P0A462	A4X915	P50080	Q7YR70	Q96KT0	Q55E44	P75711
Q98DV6	Q18801	O31696	P22536	P46315	Q9EXU9	Q5UR09	Q5UR15
Q06412	Q8RVQ5	P32285	Q65CC7	Q57787	Q0P427	Q58DE2	Q750S5
P48447	P54608	O42646	A0JYG9	P32940	A8XP97	O83880	Q32LK1
P37000	Q6UXU0	Q9A0L9	P09711	Q4Q0V1	P38747	Q08014	P72161
Q9C948	A3DGF4	Q73JF6	Q54YS6	Q972G5	A6NHG4	P93422	O43044
A3PCN6	O94625	Q6UDF7	P45701	Q54LS8	P43974	P21860	O66959
Q58124	P22502	O66112	P0A5A0	Q4R4V2	Q9J507	O95263	Q63603
Q6GZU7	P25616	P36170	O60503	A5HJM1	Q1JU72	Q54WC0	Q6P444
P30527	Q9F723	B2ZB95	Q03219	A5F964	Q7RTY8	Q9M3A8	Q9Z7F9
Q00871	P40004	P13782	Q57696	B8PYG1	Q10189	P53270	Q67593
Q7UXN7	Q8MP00	P18813	Q9CQY1	Q6CVX2	P49175	Q6IPT2	P41469
P94575	Q53143	Q6GQI8	Q47418	Q9BZD6	Q888L8	Q8QFP1	Q54GL3
Q9BQN1	Q5ZMV8	O53279	P23200	P15782	Q10934	Q8RFX9	Q7KRY7
O67413	Q8IZM0	Q55BM0	Q9D7S0	Q96MD7	O86109	Q99570	Q8R5K6
Q3V4U6	P42425	Q9IA02	Q5UQ52	Q97JN9	Q9FYC8	P12546	Q42598
Q23977	O15050	P05670	P15278	Q4JSJ5	Q03196	Q6Z7L3	P42539
O64218	A8AG49	Q8TA86	P24252	Q58336	P64561	P63702	Q08419
E1BRC3	O83966	P77129	Q9DUB9	Q5A312	Q9ZMM1	Q94AR4	Q196V7
Q02505	Q6FRE7	Q8YRV2	Q18788	Q8K4H1	Q7MV19	Q9J5A5	O74959
P38869	P26745	P37127	O93654	P44221	Q17004	Q60279	Q1E8C2
Q9X095	Q9MB58	P06624	P45408	Q60W34	Q870L2	P38068	O31882
P45866	O23406	Q9DG58	P14774	P07882	Q8BYZ1	Q9PUR0	P81993
O31126	Q5JES6	P37789	P54078	Q5JZQ8	Q00609	Q6L0N2	Q9FIG9
Q5EAF2	O31557	Q3L7M0	Q55CE0	Q8J1G7	B0JFB8	A9ILJ3	Q5H7N6
Q9NJU9	P71850	Q05181	Q9CP15	Q9ZEC0	P45301	Q32LZ8	Q96MT0
P35855	P40457	P40899	Q10952	Q08842	Q98QK3	Q16222	Q72XU4
Q9BR76	Q9CLV1	Q5NLB9	P76122	Q9U2M8	Q9XW78	Q4FS43	O95626
Q86BA1	P10946	P33775	Q9R4G8	Q9RA11	A7ZHD4	O94624	P33848
Q8CFV2	P36096	Q9E6M6	Q10PZ6	P75329	P49107	Q57152	P19004
P24919	P28193	A2AED3	Q21929	P46551	Q9UHT9	P08702	P29041

Q09791	P34572	P64543	Q13401	P45756	P09496	Q68D86	Q6UXT8
Q9Y225	Q05380	Q9USI0	P94559	P53170	Q5RII3	P0C1T3	Q5UPH8
P45275	Q58805	Q9FMQ9	Q9ZQC5	Q8XBV9	Q11076	Q01961	Q9CMP7
P95260	Q00799	Q9MUL5	A1JT33	A7KAJ7	Q54433	P0AA79	P0C5H5
Q8R205	P29131	Q9LYV6	B2A2I4	Q9FMK7	Q5UQ93	Q4WYA5	O88917
Q55BZ5	P37569	O71153	P32668	Q89717	Q6MG64	Q8GUK7	Q4WFX9
P54952	P81718	Q5UQA1	G2TRM4	Q12092	O13909	P38256	Q28199
P29306	Q91G76	P01813	Q6ING9	O28150	B8H364	Q70LC1	Q9Y6F1
Q9Y227	O86281	A2BN59	Q04004	Q5MZD9	Q83C31	Q89WN1	P44569
Q9JKC9	Q75E45	D7GXG3	Q9S733	P40974	Q96MP5	P03785	Q53U08
Q17353	P59467	Q9ZDQ0	P55370	O69726	Q57486	O04211	O07940
O32157	P45670	Q89AQ9	P17735	A1ZB91	Q99N50	Q5UQF1	Q8GZ43
P35556	C0H3W0	Q40863	P26640	Q12791	A8MU10	Q2NU62	Q9HDX4
P20405	Q8IUR5	O31594	Q75E74	A4ZUB2	Q6P8Y0	Q6P461	Q54UD4
Q7CGA7	P38675	G2TRL0	Q9C9K3	B2V8C9	A6QL50	Q47836	Q91G56
Q57950	Q25BG1	P46470	O34861	Q60294	Q9ZDY1	Q12344	O48529
P49914	O31799	O43080	P47653	P49806	P29705	Q8HDG8	Q65195
Q9ZDT5	Q9H910	P54507	O51924	O35004	P38290	P23226	P53756
Q90115	P26504	Q9RMV5	D3Z7P3	O32611	O25093	Q00104	B3EWI4
O60318	P40809	P13186	Q6FU42	Q9FME4	P52714	P02765	P41140
O13738	B2HGH0	A3MCH1	Q9LFC5	P22226	Q9ZEP3	Q9BLM0	G2TRL6
Q9KD20	Q88417	P54442	P39328	Q9ZQ34	Q25BG7	P0A922	A3MSS2
Q9UST3	O53449	Q08358	Q8T2U0	Q9FDA3	Q9LQ95	O60575	Q86I75
Q9LVT1	P21741	Q9STF9	Q6BDI9	P15033	P07771	Q9H943	Q53562
P46463	P23305	P54389	Q8NFA2	Q01416	O66404	O34930	Q58317
Q9RDL7	Q5EA01	C0H3V8	P75528	Q5XK85	O06727	O29190	Q10078
Q9SI57	Q9YD08	O67421	P21395	Q6R7F0	P81286	P95257	A1AJL2
O35600	Q9RU24	Q5XPJ6	Q46817	Q5UQC3	P63716	P36274	P45285
Q89592	P52911	P30541	P08304	O05455	Q7AA80	Q2R1Z5	Q5TFG8
P17616	Q14DK4	Q5UQI8	P76559	Q9P7I8	Q5XBB2	Q9N2L7	P40350
Q8N0N9	Q9UJC3	Q7UNR2	O04251	Q7T321	Q9BWU1	P47649	Q5UPB1
Q9Z5E9	P13847	P27882	Q5UQM3	Q568R1	P0A5B8	Q9I3N6	Q6PDG8

P50464	P76254	P80343	Q9UTR5	P53974	P80680	Q9I8Q3	Q96EN9
P87163	Q869Q8	P55532	P29835	P21361	Q19425	Q7CI09	O94520
P36022	P53908	C5DD31	Q1RIY2	G2TRR8	P83310	Q9ZVN6	Q09213
P0CI14	P24391	O55647	P44583	Q8VKH3	Q54MJ9	Q7TS55	Q3E784
Q9STX3	O13671	Q04434	O45319	P16784	Q94A82	Q5UPV7	O32201
Q3E743	P21016	A7TMZ9	A9MP50	C5DII3	Q58T65	Q9AGA7	C0H439
C3XR70	P14084	Q5UP28	Q9NPH5	P58989	P46850	P03771	P86146
P43018	O67364	Q9LW20	Q28557	Q04833	O74504	P40547	Q980I4
P42567	P0A9K5	B3EWF5	Q9LQ55	P30393	P53943	Q7CGI0	Q63QJ0
P09295	P76548	Q86VZ4	O85674	P12572	Q6ZNR8	Q7KWM9	Q9VFX1
P78605	Q2V2P6	Q5S007	P43112	Q03831	Q54MC9	Q24498	Q9TKZ2
Q8Z8E5	O67553	P05993	Q1KXP6	Q9ZE59	Q9UTB1	P70791	Q58947
A6SFL7	Q8IY50	P26800	P29818	Q5RE57	O36382	O93517	Q98FL2
P43991	P0AEX3	Q5T036	Q6AQ14	Q9UTG1	P15754	Q9VSB9	O07177
Q58589	Q8CQD9	B6JMJ2	P38810	P27989	A9HC14	Q9LMR4	Q62470
Q57951	P27379	Q08238	P77858	Q7WY77	Q9RYM9	Q94AZ2	P45385
P15260	P86208	P10234	Q9XC90	P42510	O06483	Q8WS88	P68355
O31647	Q4R1I9	Q18JL4	P32157	Q02643	Q55FC6	C5DE09	Q9KXJ6
P38332	B0XPR3	Q9BXT5	P54915	P47065	A8XDX2	P26012	P15399
Q9FL07	A4GZ97	Q72NP4	P01000	D4AIS3	Q01259	Q45358	Q8RVH5
P09885	P0A1I2	Q60522	Q12374	A8XPL7	P11864	Q00940	Q8AW42
Q85230	Q5URB1	Q50830	P64092	P37495	Q5K5C1	P20347	O74316
Q5L6W5	B1AI60	P75491	Q47745	Q10Q63	Q9K1H0	Q9WZF6	Q9C4B4
Q55116	Q9GMB7	Q65158	P03718	Q54VV5	O36394	Q0IHV1	Q9SY07
C6Y4C6	P10426	Q8L4M1	P72699	Q86T13	O06091	Q9QZR8	Q27533
O28325	F0KFM4	Q84Y95	Q9ZU97	Q93W20	Q59017	Q61333	Q8NEP4
Q6PK57	P40177	A9BGS7	A3DDH7	Q9US27	Q09202	Q9ET22	P38800
P55080	Q6DRP2	Q9Y6E0	P43988	P26305	Q587J8	Q8SQ26	Q32KY8
P49788	O07188	Q12519	Q09GK2	Q50849	Q32NS2	Q08315	Q5UQN0
O66565	O29830	Q00571	P94135	Q9Y3A3	P26362	P03753	P43004
Q8SPZ1	P15099	O27252	O67472	A7WZC5	Q6FU40	A7F679	Q90352
O67138	Q2NKV2	Q1JUQ1	P08726	P25203	Q831P3	P31236	Q5UR97

Q54J35	Q914J5	Q2HJF2	Q09242	Q0WQ91	Q874C0	O14120	Q9V2X1
Q5L7W2	B8FNN4	P50519	Q9UBI9	P53199	Q01249	O51073	P32438
Q7N3M3	Q62924	P46934	P34296	Q8BHA1	Q5ZKN5	O28095	P48299
Q83KI5	Q8ZA34	Q47499	O31287	O35926	Q497Q6	O97401	P77610
Q67ET0	P51239	Q53U10	Q9HGM2	Q99027	Q09638	Q5I149	Q6L8F9
P17791	O94595	Q9LJF8	P75978	B1ZUK1	O34350	Q12453	Q4FQK7
Q6CPX2	P16450	Q3UYV8	Q8SVF7	Q80U56	Q5AI58	Q8RU95	Q2G1F3
Q0IIB1	O28377	Q5UQU3	P01067	Q8L3R2	Q9BZR9	Q1CT10	Q51705
O89085	P13194	A3A8W6	Q54YD9	P55684	Q9NI63	Q54FF3	Q3M5M3
A6UU98	Q68DA7	P49013	P32464	P70997	O83469	Q54MZ8	Q4V7X9
P19781	Q6PKH6	Q56953	Q03546	P0C9J0	Q86YR7	Q8BVU0	A8WE67
Q12111	P03783	P96688	C4XIS0	P53253	C4V8L1	O14301	Q54CC5
Q6PI77	P31446	P55103	D4GPU7	P02775	P53585	Q502E5	Q65371
P34026	P84988	Q58048	Q5NDL3	Q54PG5	Q5SF96	C0H3U0	Q84V39
P06486	Q54FK5	Q9EXD2	Q2V2Y0	Q5XF75	O95500	Q96BF6	P53129
Q9SG96	P31381	Q914G3	Q86HQ8	Q99210	P00739	Q752N3	Q19189
Q566Z6	Q8TDU5	Q5URA5	P09730	Q02817	P02671	Q5LLE7	Q08CL3
Q08486	P27750	Q9BPD7	Q9KSB4	P04538	Q64964	Q9LK58	Q9FG85
Q9JMS7	P68571	P48960	Q9YB03	B4N369	P31226	O60052	Q92396
Q8XJL8	P55461	Q07967	P47273	Q5JR59	O07009	P37270	Q54WE5
Q54C55	P00143	Q10268	O94594	P22577	Q07978	Q9LSV3	Q9HLP8
P80564	Q9SM43	A1L253	Q20010	O32130	Q9XDM4	Q54JL7	Q54NW7
Q54LV7	P75859	P79017	Q96LB3	Q12359	P43606	P17177	Q6FM98
Q6T6T4	P16028	Q9D676	P23451	P38079	P70175	Q92JJ7	Q6R7J9
P13284	P36626	O64511	Q9BRK5	Q54V44	Q92JM6	P37294	Q9ZLE0
O28790	P55240	O36017	Q50047	E6RM71	Q9PTL1	O29145	Q9LUH2
Q3E7Y4	Q8D3J6	O83645	Q54DB1	Q8ZH96	O55749	Q3IRT2	Q9P6L9
Q84WW2	Q12V67	Q8NEN9	Q88FY5	Q750S2	P10259	P53918	Q80TA1
P65574	Q5E9L7	Q9NQE7	Q44840	Q58205	O28476	P0C1T6	Q36967
Q60282	Q8LL17	Q49775	P39713	O74920	Q740Y5	B1VTI8	P61032
P20500	C6Y4B8	P25954	P39497	Q5TCY1	Q9LZ19	O95562	Q96PY0
P08138	Q14194	P21521	O55736	Q4KWH8	P45434	P23355	Q880Z2

Q5VZP5	Q7XTS4	Q0V9V5	P42707	O06079	Q9ULI0	P04201	Q9M2D8
Q5UPU2	Q02805	P17055	Q9P7N9	Q05016	Q60414	Q59R24	Q495Z4
Q9VH95	Q39202	Q93Z24	P25806	O28611	P80220	O31523	Q9KJF0
P0AFW8	P55678	P45552	Q6FMV8	Q08632	A5VU91	Q5RKN4	P68583
Q09871	Q9SV72	Q8RF47	Q750L8	P75737	B0SII1	O32007	Q6WB94
O42996	Q14002	P31561	P24871	Q0V987	B4UN34	P87309	Q03611
P16779	Q9TT92	P03239	Q6CIT9	Q62471	O55237	P96600	Q5RHU7
P21602	P04370	Q26418	O51750	Q9MA92	P37007	A8X0C4	Q54VI1
Q75W84	Q9SV05	P62368	Q6FJ45	P14109	P03709	P48334	Q13YI7
Q9P6K5	Q5XC60	Q9UPY8	Q9D9W1	A2RI45	Q9A777	A1WUX4	P47699
Q6NV34	B2C6F2	P23984	Q90125	P09425	Q3T0E9	Q5FKQ9	Q9JWJ3
Q93Y38	A5UUJ5	O94279	P0C655	Q2S433	Q5X8H6	P38971	Q77MQ6
Q96X49	A7X4L4	O61397	Q8JFG3	Q12511	P42536	Q01026	Q8BUP8
P29582	Q5FRR4	Q6YNI5	Q8ZU30	P29815	P39042	P33554	O05406
Q3AAW3	P18670	Q70Z53	P32278	Q5I133	A8XEQ8	P52291	P07748
P55815	Q8CH01	Q7JK24	Q9H7H1	Q29BR3	O35609	A2YGP6	P07789
O07621	Q7SXX7	P26225	Q54BD1	Q5TCS8	Q4P7C7	P18896	P51102
P31382	P44279	Q9SVZ9	Q5EFU4	Q2NKQ1	Q46943	Q75JK6	Q02373
P13092	P68656	Q56564	Q9ASR4	Q9USK0	P53888	P42955	Q01344
Q9STY3	O26638	P76551	Q03550	P44053	P68178	Q5UR53	P55677
Q9VPT5	Q54WD7	Q9VAC8	P93227	Q7SEW2	Q3ZBA3	O06477	Q8I5I1
P47111	P75169	A7TQS6	Q640Z9	Q9SKK8	P25659	P40949	Q9XV66
P13082	O30300	Q97CA8	Q88426	Q81CW7	Q8BHB3	Q59750	A8QZJ5
P25571	Q54MC8	Q9A4F4	Q9FH28	Q86CS2	Q53GA4	P40761	Q91ZW7
P09565	P0CV97	Q8VBV4	Q57651	P32800	Q5GH76	Q57895	B7Z8K6
Q84MC0	Q8SVC0	O05813	Q6R7L3	Q8N5Q1	Q67669	Q9H169	Q9IW99
A0JNH1	Q08DK1	Q8AIH6	Q9CLQ8	Q8T690	Q8TEV9	Q04912	C7YY76
A5E0V1	P92127	Q03021	Q8QL28	O29998	P14499	Q573B8	Q5UPD4
Q8KCK7	O32166	P30606	P03481	A2YP56	O49146	P40503	P0A369
O28598	P35648	P45176	Q9RY24	Q55FQ9	Q58411	Q12155	Q553D3
Q92IC3	P65530	Q2KIL1	Q3E7A4	Q4R6N0	Q5EAB0	P52135	Q27245
Q9FLP7	P41771	Q92ID7	O94644	Q89A44	Q9I310	P13234	O88634

Q65132	O13476	A6NMS7	P0C8Q6	Q9C104	Q9SWE5	P37182	Q5UR19
A8LS52	P72151	Q8MUF9	Q8K0W3	Q9FIM2	Q82PZ0	Q80T22	Q5UQ67
P53904	B4YNF6	P54468	P40691	D0KQM8	D1BJD0	P25136	Q4FV24
P33050	Q90873	A4ZUC6	Q23544	Q94AS5	O94255	Q5K130	Q58041
P19076	Q30YK6	P34355	A5F4P6	P94777	Q9J5H4	P27178	P53906
A1YV58	P38216	P42490	A5IYE2	Q9MFP7	Q17766	Q8C7B6	G2TRQ9
Q08361	P34163	Q10197	Q8AXQ3	Q9HD23	Q9HKE4	P41711	Q57913
Q755J5	Q5UQB6	P0C7R1	Q9CWQ8	P27833	Q32LH4	P11528	A8E283
O29156	A0LDU6	Q8EQY4	Q09116	P0AAR8	O50161	P02925	A6Q574
Q9W358	Q5UP04	Q8SU90	P30113	O01839	Q8LA14	Q54K67	O60260
Q9G058	Q6H849	O60110	Q10510	O46584	Q12114	P52418	Q9P0W0
Q58726	Q5XPK0	Q9Y238	Q9Z896	Q5UP95	P44293	Q04691	Q9K6W2
P93319	Q5UQM2	Q9U2T3	Q9P6N4	P75991	Q04RM8	P09962	O14269
Q4WVT3	Q9VWE6	A3KNS2	P16005	P28993	Q58862	Q3TTY0	Q5V213
P34138	Q8NHQ8	Q9SF78	Q82XD4	Q0V9K2	Q96DC8	P0CY93	O60178
Q553R6	Q8VJ51	Q06758	Q4L4W4	O83819	O43103	O88338	Q5JIH9
Q5UQ74	Q24X59	Q9XGZ9	D5AQY8	Q9Y7B3	Q9UBX7	P52311	B5XQ62
Q04921	Q8IUC8	O55760	P0A4N1	Q6FJ19	P44842	Q9FHX2	O17624
Q32LF5	Q5UQJ8	Q6CQN1	O10284	Q9FH80	Q5UQC0	P38148	Q9UJ41
P35800	Q8TD86	Q2KIJ2	Q7NAZ3	Q92213	Q12047	Q9I969	Q8BGR2
P13486	Q8GW57	B2RIV3	Q5VTM2	P32972	Q9CAG5	D4B5L8	Q9NQ30
O32266	Q9UKA4	Q9C1W3	Q914L3	O74507	Q9RQ88	Q9VR82	Q03686
Q4N4N8	Q9Y6R7	Q5UQG1	Q8BGW2	C4NYP8	P27894	P35733	Q13163
P48329	Q876N4	Q7F0R1	P31004	Q890M6	P97435	Q8ZDB2	P45944
Q6CGE7	Q6PHG2	Q9NV44	P40527	B3A0R8	O94537	Q8MNU8	P20206
Q7M758	P75995	P25565	Q54UC9	Q55EL3	A1L2S8	G2TRM7	Q6F059
P40603	Q7XWS7	O94280	Q5URA8	A4D0T2	Q2RBE3	P15234	A1L2E4
P08794	C0H465	Q92502	A7SDA8	Q8VZ95	Q8EVS4	P38194	Q47887
B8HPI0	Q5R4J9	Q2FED6	P0DH87	Q1RHI6	Q6BT11	P25666	P00121
Q44243	Q57738	Q1IEC3	Q8BCV6	Q08373	Q8WWU7	P40995	Q6QNF8
P37711	Q8VYP5	P32812	O09452	Q9H9L7	O49326	P0CP05	Q8N8H1
Q504F3	P45950	Q8TX56	Q67YC0	Q0WQQ1	Q00749	Q4IJW4	P0AA33

Q0Q465	P22231	P36678	O05510	P44791	P09662	Q58377	Q87KN9
Q09230	Q9D478	Q9UTQ8	O61902	O13986	A6NMD0	P71079	A3DHI6
P68434	P20049	Q0B746	P19824	P10836	O07014	P15897	O67428
O31067	Q0P4K8	P38943	Q57427	Q54M18	A2VD00	O02387	P0CF96
Q10263	O08358	D3SYZ8	P28908	P94951	Q29236	Q53HC0	Q13610
A8XKV0	P29010	O28606	Q25BG2	Q9SI02	Q6FJ28	Q00733	Q18014
B2ITV8	P83977	Q4WQL0	Q0IHQ3	O48881	P45905	Q9Y2J2	Q03218
Q2YIT6	Q7XYM4	Q680Z7	Q07108	P40531	Q8Z5A6	Q17QN4	Q59601
Q9P6K1	Q75EN9	Q58980	Q10935	P33757	P07394	P41949	Q9LU94
O66758	O18824	A1A4L0	Q6CUE1	A1S0T1	Q8QVL7	Q09835	Q9J4Z7
A2WYE9	Q9A5D7	Q6ZRP5	P54734	Q8S5M8	Q8T4E1	Q9NDH7	Q9BPQ5
Q95224	A7TN63	B2STP6	P38464	Q9CWL8	P43302	O74750	Q0WLC6
P19276	P83903	P39083	P60172	Q9XTN2	P45524	Q96266	P75077
P0CW76	Q76QZ8	P59037	Q8VHG0	Q48464	Q72VK0	P44747	P36935
P43446	Q52983	Q94AA1	Q9IAY4	P44942	Q45774	Q8IUH5	Q9ZE18
Q93PU8	Q86H39	Q2FLN5	O22959	Q48IV1	F2Z3M2	A7HDG7	Q91F73
O83992	Q80Z19	O49603	Q9A354	Q9ZPR0	O74497	Q9UTD8	P0A5Z1
Q6BYP8	Q54F97	Q07454	P28265	A1XJK0	Q55792	Q00245	Q8CDZ2
O87908	A4W0K2	Q9D727	Q9CAT6	Q4UJ67	O34849	Q9UTG3	Q05473
Q9NZK5	Q14SY0	P76219	Q3EBP0	Q6R7I1	O13553	Q6NTF7	Q9U943
P42575	P38109	Q10244	P32793	P81913	Q8GWL2	P71065	P38992
C8ZJM1	A6S3E0	Q27995	Q7TSL0	O14944	Q54KD4	Q93YW0	Q8QVL4
A5FYE6	Q9BKJ9	P96913	P96010	Q197E0	Q73J26	P71014	Q5FWH6
P54338	Q24K16	O30280	Q5XA33	P12533	P41473	P53929	P06484
P47056	P19363	Q13SY1	P38752	P29739	P87027	C4R591	Q8VI84
Q9RMZ8	P20843	Q8N131	Q5H913	B0SDA2	Q6FLP6	P0CY46	Q7NI46
P0A617	P28732	P07077	A5U0B2	Q4U3U9	Q9QYJ6	Q0W4W4	Q54W04
B5X3I1	P82974	Q8K1R7	P48362	P53681	P43975	Q55BF2	P55709
P12058	P86888	Q55988	Q9SZ95	P0C853	Q57321	P08399	Q54GE8
G2TRN3	P46058	Q5UQG9	Q8MR31	P29912	Q2TBA0	P42109	Q9QY39
Q6CRE9	P61154	Q55DQ4	P32083	P70583	Q6R7K0	O52071	Q10474
Q9AJD6	Q91FU0	Q9URY8	Q24342	Q08E20	O13932	Q5UP68	P75347

O30173	Q8RKH1	P32912	Q69554	Q54UF5	Q6X4T0	Q9LVG9	P18192
Q9USX0	Q9K548	Q94AX5	Q7S1I0	Q55GT2	P13411	Q9TUH9	O57774
P26489	Q6GZS5	P00541	Q2KIS9	Q9VAS7	P75139	P96638	B1P1B0
Q6FYR2	P41708	Q10352	P15977	Q9MBG5	Q50232	Q4GWU5	P83179
Q9I492	C4YG73	P48672	O26106	Q10435	Q6FVJ1	P11437	Q54LD5
Q8SSH7	P02518	Q54WR5	P68357	Q914G0	P79058	P40001	Q9A8J2
Q80Y83	A1CQC3	Q757H2	O26773	Q197B1	G5EEM5	Q9AS36	P0ADM9
P31624	P86203	Q5BL29	Q72RH6	Q86DA7	P39139	Q0WRB2	Q5L9E0
P96656	P55376	P19557	C5DQ18	O15273	P46378	P97056	P97636
P28549	Q96C74	O75157	Q5WF04	P23644	Q1MTR7	A9BJX6	Q9HM28
P07620	O67669	Q10269	P02855	P44114	P20356	Q57942	Q6CHP1
P81511	Q5R0Z7	P54360	P76164	B0G159	Q10417	Q54EX5	Q54QR1
Q89AV7	C5DRG2	Q96QE4	A2ZFY7	Q98930	P23327	Q9NPH9	Q2MJU7
P49193	Q229Z6	A3KGK3	Q6GZU8	Q55CW3	P23550	Q32KS0	Q96N64
Q6R7H6	P81281	Q89AF0	Q5UP91	Q8VC88	P38700	Q45271	Q03956
Q8EXQ7	Q9K6A3	P45586	P42837	P47533	Q61DP2	O29740	P40488
Q96PV7	P00721	P12049	Q8TBB1	Q9HDW1	P54326	Q5XJK1	Q8TWX0
O67523	P73894	P17118	A8AK82	Q1A261	B3STN7	P41561	Q12YW2
P86982	P95008	Q940P5	Q197A3	Q5P5G6	P18169	Q6DBN5	P86953
P46557	Q06SD4	Q38668	A7X0P5	Q2KIH1	Q59683	A5E057	Q9R9I1
Q10408	Q9XPI2	P11372	P39160	P54337	Q58FG1	O06708	Q89EW7
Q5JSL3	Q9P7W4	Q8I467	Q197F8	Q54757	O02769	P0AGM6	P53755
P24583	P15821	P81232	P96611	Q5ASP0	P22776	Q8NI77	P04683
P01049	Q08734	Q8VYJ1	Q10497	P54373	Q97B34	P48935	P32629
Q94402	B0BRI0	Q8KLB6	B5XAM2	P54330	Q8RBR1	P08948	P51736
P19040	P57222	Q58098	A2XNL6	O94602	Q9V979	Q9P6J1	Q9SCY5
Q6ZWK4	Q6P0S3	Q9H321	O34988	P36881	P39107	Q2L897	Q21890
Q60370	P71979	Q8LFA9	Q6S9Z5	Q08EC4	Q04835	P34610	O13843
Q54P13	P77694	O30602	Q9UUI8	B0G126	Q6BTW5	Q46812	P0DJ88
Q9H1Z9	Q9C1K8	P37558	O27318	O29669	Q90577	P03998	B8CAZ5
O80927	O29730	Q7NCS0	Q91WT9	P20829	Q92J60	P39602	Q54X31
P64427	Q01025	Q9KU25	Q49AS3	P89679	P07167	Q46927	P87239

Q09802	Q48943	Q16048	P21824	P03412	Q2M238	Q553J1	Q4P8R5
Q9PX43	P48305	P76042	Q2V369	Q08DF7	Q9D9Z7	P13341	Q9NRW7
P31759	Q91400	Q9FLF4	Q12305	P0C7G9	P47121	Q9M9T0	P41366
P0CI02	Q8N9K3	O60132	Q8RWS8	Q8IX29	Q6BIT1	Q08345	C9SPU8
Q9XIE0	A0LSL0	Q57785	Q50585	Q58984	A8AVK0	Q6Z3A8	Q3ECI7
Q07536	B2VEU3	Q54BW1	Q9E6N5	O07604	Q9US12	Q6CIJ3	E6WMH4
Q2TB02	Q9LU68	Q6UY62	P06936	Q04948	Q3E8B0	P36003	Q9AVB0
B0R5N7	A5PHT0	P60589	Q14439	Q925F2	P28723	Q9M122	Q9JWE9
A4IIV4	Q58764	P38452	Q9VP04	Q2NHN9	Q9P6Q0	P83997	Q93UZ0
O14145	Q54SK3	Q8TDM0	Q9VAU9	A1BAK9	Q9ZU90	Q01467	C7C422
Q3V4S2	Q0IIL4	C1JJY7	Q55EA1	Q6ENX7	Q00298	Q8MII8	Q0UE25
O29665	P40580	O86362	Q65T32	P81581	Q44902	P27103	P04094
Q12450	Q584D5	P03854	Q9VEG6	Q01353	Q8VZP6	Q2KNE5	P16065
P39163	Q18J06	Q9C919	Q26896	O83691	Q9HCI7	P37491	P45913
Q09464	Q86YW7	Q9FY68	Q9Z994	Q57066	Q8YX97	P71044	P0ACX3
Q9NL39	P18963	O94627	Q9WIB9	O13632	P45704	O46587	O67893
P44623	Q8X092	Q7VG29	Q72K16	Q58571	O96363	P28495	O73622
A3MAS4	P43484	Q1LY51	Q96N19	O69740	Q8TDR0	Q8EUM5	P17334
Q12202	P0C5K7	Q9D9A7	P0C2S6	Q60DN5	Q9J517	P16552	P42529
Q7VFP7	Q12058	Q9ZC49	P52047	Q9BV10	O13808	Q9CA63	P76364
Q5UQP7	P64546	Q91X13	Q54SS8	Q8DG35	P75463	P76499	Q9KYX0
Q6MDK7	Q8NA29	Q9AIP8	O66457	Q75EJ1	Q53E76	P48353	Q54K35
O84543	Q9Z868	Q9P7Y8	G3XDA7	P42864	P94488	P25910	Q98855
P64923	P85227	Q87AR8	Q06852	Q76LV2	A6QAM3	P76092	Q67YI6
Q9ZKW3	Q9LSQ6	Q2TBS3	Q9HC29	F7V999	P86195	P31383	O31917
Q8PU66	Q5UPS1	Q19683	O66975	A2SY66	Q8QV02	P42535	P60154
Q9HHT0	Q54TG1	P80544	P14209	Q02596	P42664	P44446	O83827
P51203	Q97BG7	P50505	Q03376	Q9NTX9	Q60312	Q5PQQ7	Q9YDX7
Q3E7A8	Q9SR01	P50053	P16699	Q5JT25	P23283	Q7TQN8	O74913
Q23TC2	P21849	P77716	P33010	O24575	P41735	Q9XF89	P0AEA0
P0CB23	Q05497	P82632	Q7U0N5	P25338	Q9PHW6	P01633	C9D7C2
Q91VC9	D2Y2B6	Q54DF2	P96825	P96285	D4GXC5	Q9LE59	C0H3Y3

P55502	P22751	P97799	P03597	Q2RSB3	P81589	Q9CNW3	Q9QJ50
P55149	O33833	Q8IYB1	O57593	Q6C1E4	P24620	P0CK12	Q08220
O29292	Q46631	B1P1J4	P20748	Q92932	Q2UQY2	Q8N8X6	Q9RZA4
F3Y9Q5	Q6H7J6	P15442	Q8GXR9	F4HTM3	Q9C109	O34559	P32562
B3A003	Q48899	Q8BGK9	Q9KK91	Q6W0C5	Q8N6C7	Q54NK3	A8E657
A8JUV0	Q8VY13	Q9D3V1	O06875	Q04413	Q95PZ0	Q9WU47	Q86IG2
Q8XL46	Q19468	Q07940	Q8X8D5	Q09662	Q8WZK5	B7FRE8	O94670
P0C5N1	Q57716	A6QGC0	Q54XI9	C6A4F1	Q9KFF1	Q9WZR1	Q4VT39
O34779	O32239	P33336	Q0IIL2	P20758	Q54JQ0	Q8SVY8	P0DJ66
Q8RX01	P58701	P33518	P27816	Q92360	Q8PZ55	P39726	Q19PY9
P71063	Q8VBT1	Q9NQ60	Q9WUS4	Q9ZT49	Q01267	A4ZU89	P18152
A7TGR0	Q92356	P43055	Q24298	Q24278	P33366	O85728	P76319
Q8TYM5	B0R758	A6ZQE9	P45386	Q9UNE0	Q9UK39	Q5UQF5	P55515
P75049	A2ZBG5	Q54XR2	B0WC46	P30219	Q9ERK0	P06558	Q05298
Q9PJJ6	P50864	P53510	O58968	Q9M0D0	Q96DN0	Q5RCW9	P37298
O74446	Q58310	Q9P7E9	Q8K2H3	P85190	Q9SYG9	Q45583	Q9C6I2
B4RTA0	Q8GR69	Q91X34	Q8CAS9	Q9FFZ2	B0G194	Q93U24	P43045
Q3SHI2	P26723	Q8GEK8	Q71RG6	P55694	Q6P1H6	Q9QXE0	O31695
Q5VU43	P42253	Q5UQT1	A5UZ79	Q9P3E4	B2KIK3	Q06152	Q8CFW7
Q9Z469	P31956	O51060	O74917	P33362	F2JXJ3	P0CY58	C4Z3C6
Q91F69	Q6AWW0	P15333	Q92367	O53474	Q9H095	Q06734	O14146
P36324	Q8RB27	Q6NRH7	Q9LNC0	Q04005	O69481	P37020	Q29175
Q3E960	Q5BGR2	O94664	O69653	P85992	A7Z9Q6	C1H3X3	C3P6X0
P19385	Q58402	Q10136	A8JAM0	P34436	O42970	Q86B91	O94888
O34402	P00127	P48799	P0CN93	P46104	Q755M5	B3A0S4	Q44335
Q7T2D0	Q3E793	Q8BU27	Q2J7K4	O30135	P0ACE8	Q97YC7	Q54SZ9
Q3E8Y5	Q9CEL6	Q8R8N2	Q573C1	O32083	P59935	Q8JZZ7	Q75B70
P58094	Q6SZ87	Q9LVQ8	Q9Y572	O07684	Q58717	B2KE54	Q9W7S2
Q6FLQ1	P30879	O34626	Q6DIB5	P83304	P38767	Q9DHF5	Q03304
Q9X1D7	Q55EI3	Q52926	O31769	O93967	P34658	P13007	Q940Z1
Q7TS68	O29261	Q93WI0	Q7RTP6	O66741	O29350	Q69YW0	Q197E9
C0Z7G8	O75970	O60721	P30291	Q9KER1	Q7ZVC2	P30305	Q96Q40

Q5UBV8	P58337	B9MS71	O31948	Q5V4L7	Q6ZMY6	P0A928	P03046
P87178	Q96NE9	P08959	Q00147	Q30KJ8	Q9P329	Q09499	Q9ULL4
Q5VT28	P41413	A8NS89	Q2TWP5	Q0IWF3	Q13316	P29518	Q1RJQ4
Q8G6J6	P38842	A7YWP4	Q8N6G5	Q9P2N2	Q70LB4	Q6AUW3	Q54JZ1
A7XYJ6	Q6GZU5	P05782	P26703	Q8N6S4	Q8K2L8	Q9I4U2	A2SMA7
P52143	P96642	Q9CHL6	A8C927	Q18F28	Q01819	Q9HCN6	A5D2M7
P50907	P34654	A4STH4	Q03904	Q14153	D4GUF6	P53536	Q01024
Q712K3	C7GJG9	Q0V9S0	Q2J9S4	P56620	P92535	O29485	Q08428
Q96Q07	P0AEE4	Q497N7	O30176	P64541	Q8GWK1	Q65X23	Q04697
G8FZS4	Q50701	P45217	P63708	Q46888	Q40189	Q06GK1	P47450
P0A3D4	Q8IU80	Q95Q39	Q6NYU6	A7MBI7	O83081	Q9ZKG7	Q99394
Q54UQ4	Q6NDF6	P34357	Q8CFM6	Q9LX93	Q18319	P26223	P54007
O31725	B3GSH5	P34494	Q8TVI1	Q9C6W9	P60979	Q9LEX6	Q9HPH7
Q18685	Q6BQJ1	P55577	O28482	Q9ZU29	P25728	P55588	O42563
Q58056	P91416	O48397	Q5SW45	Q54ZM4	Q2KJ25	Q9SJC6	Q9GMU6
Q9NJD8	O60303	Q6WG17	Q9UW12	B7F138	Q9UUB6	P64176	Q9P2Y5
Q9HCM1	P31686	Q54JU0	P97224	Q57124	Q9C660	O28768	Q6UDG1
P79679	P93283	P77087	P09551	Q04266	P80481	C0H3T2	Q5UR52
P03942	O66819	Q9BX66	Q01802	Q94AA4	Q6CM46	Q7VHR4	O29259
Q6URH7	Q9UI32	P0A2U5	Q09420	Q9VG55	P0CM51	P96972	Q0QLF7
P80675	Q804S2	Q5UPW6	Q6BSK5	Q5XIP4	O54912	B2KBZ0	O17901
P38769	P32450	P52731	P38350	Q9FI39	Q5UPQ2	P89113	Q9RXG6
P06459	P21299	P87264	P37297	Q6CMB5	Q6FWS7	Q09214	P09818
O31719	Q9P7T1	Q9W6R5	P03725	P96747	P39494	P44174	Q12184
A1L4Y2	P26688	P17213	Q9P3B1	P81131	O34368	Q9UP38	Q2KIK2
O86225	O66853	Q7ZWG6	B1Y382	Q1MTR5	Q9ZBL1	P32155	Q09556
P26486	P52961	Q9M392	P00771	P03413	P28735	Q1BU34	Q58642
P39564	Q9XWQ1	Q7XAK4	Q53222	P15518	Q4RU86	P59047	Q54YP8
P75354	P75253	Q5ZHN4	Q6ZMU1	P11449	P24720	Q94A64	A8XWC4
P22184	Q11104	P0C891	Q44141	P83516	Q9LQ47	Q10094	A8XNJ6
Q4V8G0	Q54KB9	P94576	B4EVU5	D6C4I7	P48236	Q831A2	Q7Z3B3
Q58514	P09522	O01949	Q68FR2	Q52WX2	C4R492	O14093	B3LLP9

Q2W3D5	Q03311	G2TRQ0	G1C1T2	P29817	Q8I145	Q40297	Q14687
P08390	A0RXK2	Q8BXQ8	P0A4V1	Q54TR5	B0BXL6	P40781	P75193
P43787	Q11YP2	Q93LL2	Q19459	P57018	Q6ZUU3	O74876	A1IGV8
Q9VGX3	Q76NV7	Q75JK0	Q3SZ44	Q65670	Q08316	O53904	Q6NRI1
A2VCK2	Q58102	Q9HB20	P54432	P69594	O94400	P0C8P2	Q2HJM9
O13990	A7Z061	Q9RMW2	B0S296	P62499	Q9A3S1	P60967	Q9D0B6
Q1HPL8	Q9LK94	Q4R910	P53271	F1MUS9	P23647	C1G2L9	Q5VVB8
Q9DCS1	Q9CQ90	P45597	A4FU01	Q6B2I9	Q9CNE3	Q9UU85	Q57KZ6
Q3TTL0	O46166	Q9SR77	O53278	B0R5L0	P27599	Q9TM18	Q7SGZ5
Q9NRU3	Q9VGU5	Q96IV6	O08340	P83742	A8A9U0	Q8X7G2	Q58537
P14220	A4W435	Q7ZWY2	Q9H2M9	P0ABF5	Q9P9T6	P38269	P81921
Q70LD6	B2HHZ8	A1A5P5	Q0P3P6	Q1I0U3	Q6UUW5	Q1BUR8	Q67706
P46838	Q9FGY4	O34640	P42724	Q8ENS1	A1B3I3	Q3E829	P30789
P98052	Q6ZST8	O83607	G2TRP8	Q05785	P67446	P0DJ41	P16760
P30235	Q09851	P39742	Q05119	Q17R89	Q6GDZ1	P0CAH8	Q0JEZ8
D1FNJ9	Q9XA16	Q7NQB1	O13641	Q9NS75	Q9UHR6	P03787	Q07596
P25361	Q80XH2	Q99186	Q9Y9D9	P22474	Q12261	O36385	Q4L7X2
E9Q6B2	Q9C1S9	P25475	P17157	Q50290	Q9VR52	Q1LZF3	A8MRC8
P42093	P58335	O59697	Q97U03	A8XWD1	Q0UEB6	Q54EQ1	O88575
Q91FU6	Q66654	Q8RKI7	Q9ERE2	Q0AYD5	Q88894	Q1EIQ3	A7FM05
P11365	P17407	O69227	Q9KI13	Q9CER7	P54488	Q9DUC2	Q9XXN3
O67892	Q8CHJ0	P84117	Q8NGC0	Q9UF83	Q8N394	Q0TRE4	Q38488
Q6CNC8	P55185	Q03548	O78420	Q938C8	Q5NGW7	P44263	P16064
P46680	O81025	Q86AY4	P55607	Q20332	P54492	Q68EN5	P96659
Q9NUV9	Q8QL27	Q06451	Q556R9	Q5TAA0	Q4JVQ1	Q9SMJ3	O06391
Q94KJ7	Q96AE7	Q3E7A2	Q54727	Q9D992	P16172	O13824	Q5E9A4
Q05613	Q91ZF1	Q6XCF2	P55479	Q9AT00	Q01076	P16746	Q14108
Q6ZVF9	P47048	Q96253	Q62077	Q94A65	O66127	P81171	Q21339
O22151	P39261	P46802	Q86H65	Q48677	A7RM45	Q95000	P23163
Q6BUM6	P0C7U5	E7Q2L9	D0ZI38	Q93NB7	P21686	Q8T2I5	P08800
P12616	Q9PL35	A6MWT0	Q24475	P27194	Q9H808	Q40546	O05524
P32418	P58036	Q65674	P38232	P45374	P30646	P57999	Q5UQR1

Q54JY7	Q55F71	P34328	Q8NA54	D3UX49	P64749	Q9X697	Q9UTD4
Q008X5	Q12218	P50100	Q96GE6	Q9KD45	A6NG13	Q10932	Q54ID7
Q1C7P8	P30748	Q5UQT9	P10392	Q9V6G5	Q58304	C5D5S0	Q08ED0
P48368	Q54LD8	O15926	P21362	Q9C0W1	Q6ZRH3	P59590	P75606
P48416	Q9EXD5	Q9P7V8	P12780	Q9P7Q2	Q6R7D6	P09945	P05841
P06875	Q9CR76	O62682	Q8UEU8	Q10S58	Q7XSV4	Q58DN7	Q3UPL5
Q08416	Q64766	P45590	P71696	Q4JXJ7	O14336	Q3TDX8	P49584
O31858	Q5ZIR9	Q9LUL6	Q5B288	P26725	Q9LRB0	P0A5D7	Q88FX8
A1K3G9	O45924	Q25BH9	P38233	Q9YW20	P07663	Q3Z992	O94483
P91127	B9MRX9	P51702	Q8C8T7	Q46971	O74922	Q44007	Q04341
A6X935	Q52118	Q9UKI3	O14256	Q5I2D0	O77480	P93028	Q58729
O34593	P39975	Q9GZU5	Q58EG3	Q04383	Q05B54	P15155	Q08736
Q8I817	P45515	P53348	P22365	Q75A58	Q9SZN7	O83461	Q49939
Q01752	B6ULW4	A7KAN7	Q9I747	P87176	Q8NEV9	P58593	O53573
P10835	P82998	Q5UQT0	A5PJN0	Q6FEP2	D5L5Q8	P0ADM2	Q95SS8
P85317	O77775	P53969	P47044	P51131	O34414	P76097	P39752
Q54VL5	Q86A83	Q9FIQ1	P75208	A5WFM3	O05246	O74411	Q58005
Q09917	Q89AL4	P59025	Q8NDB2	P0A3R6	Q23917	Q58318	P81898
P31186	O94600	A3CQ04	Q9UKK6	Q32NC0	O06629	Q3T0K9	P38451
A4D250	P50544	Q09373	O75678	O74395	Q84168	Q9Y117	Q01109
P0AC25	Q9SRE4	P0C198	P33115	Q8U2Q8	O94486	O95466	P40523
Q969H0	Q9C0Y2	Q9XUJ7	P71380	Q7SEY9	Q06317	Q9D2H2	Q24211
Q9ZDG6	P27443	P26143	Q924P3	Q9FKJ0	Q3E9F0	Q6BEV5	Q54SR0
A7XQ02	Q27571	A1SI37	O05795	Q96MS0	Q9FPS9	Q9WYG7	P51282
Q2USI0	P77416	Q55914	Q6ZS62	P09516	Q99N46	P86204	O64221
Q51887	Q58157	Q57947	Q9UTC4	P13212	Q554K2	P22573	P34713
Q68EK9	Q63661	Q10215	Q9LUD7	Q03CB3	P40085	Q8T197	Q0ZGT2
Q86YS3	Q47860	P87050	Q84JR3	P25639	Q874Z5	Q10364	Q06401
O31994	P0AEJ7	P20194	P03936	B4UN83	O75264	Q55CK2	O14836
P37363	Q06696	B9DK35	Q9WIJ8	A8MZ26	Q32L10	P32782	A6QQP7
O04086	Q5UZH5	P0ADL0	Q07084	A6NKX4	O32087	Q034G9	Q32905
Q10190	P47166	P16151	Q17389	A4ZUD9	P29824	Q5F4A1	P0CK58

P22919	P13504	Q8IN23	O74981	Q08DK0	Q6EBC2	Q17851	Q5UR01
P0C724	Q8BJL1	Q55FT5	Q90ZD5	O32221	Q54K76	P41729	P42101
Q9CIR9	P53184	Q56714	Q54EF5	Q47899	P18924	Q5WJQ2	P04288
P29122	Q9H0C8	O07917	Q9JIL5	B6H6F3	Q6ZTK2	Q1BS37	P81943
P00132	P34363	Q4PHC3	Q8TR06	A0M3K2	P47022	Q5UR74	Q2YL70
P40125	Q9UTN7	O60165	P22963	Q5L521	Q94CH5	Q17Q91	Q89250
P40180	P0CE96	Q9SZ52	Q3V4V7	Q18007	P0AD71	P21647	O28022
A3DIM5	Q2FVK2	O94394	Q21059	Q9S4D5	Q8TGL8	Q91G50	Q7L273
O17264	Q8L5Z7	Q59284	P75745	D4AKU9	Q0VA04	Q50657	Q57755
Q9KFG3	O68215	Q8TC26	P97844	O13898	Q64761	Q6C3D7	P28151
P94393	Q86283	Q8H0T9	P71053	Q5P5G2	P11384	Q8N1V8	A9CM07
P17687	Q9PQX9	Q8SWP1	Q45FA5	Q9VJJ7	P55454	O83463	P34973
Q1QPM0	Q6H6R9	Q940Q3	Q9H089	A8H9I2	P53156	Q6BYF0	P37617
Q09774	Q62101	Q0VCP9	Q5UPJ6	Q9NQ76	A4H7A3	Q54C45	P90761
Q8KG79	A7TQP0	D4GQ16	Q9FGN8	Q9EQJ0	Q04756	Q9RVF9	O05503
O58371	A9GFF3	P09294	Q46JW9	Q6FKB4	Q6YFE5	B3EU56	Q86SA8
P0DJQ8	Q9LXJ6	D4H539	B1AJZ1	A8FRQ9	Q0IHW6	Q9DF69	Q6SW14
C0S902	O49696	Q9LSS5	P55491	P50834	P48611	P41698	Q03442
Q8W471	P18612	Q9GYV5	P40977	Q6UDL6	Q7TSQ1	B5FY93	O67189
P14184	P37749	O52057	Q10917	Q9LTV6	Q68FQ8	Q65RN5	Q136A2
P43253	Q55B06	P80866	Q58DH7	Q01594	P08F94	Q9UTI9	Q9P7T0
Q5F3D1	Q7L099	A0JM08	Q9HYH5	Q9UFD9	Q9BYM8	Q9ZDX7	Q5UQ04
P33705	P34001	P19278	A0LDW8	P38732	Q54J37	Q9Y6Q2	P22099
A8Y3V5	P0C1E8	B0G0Y8	Q15043	Q9V4P1	P24132	P45685	O10303
Q914J8	Q9LNE6	Q9W5G6	A2A7Z8	P80872	P17148	P37645	Q14684
O31506	O83273	Q17QK6	Q9H400	Q9YA72	Q8FHF4	Q9UBG3	Q10668
Q04401	Q95ZE3	P55335	Q6MX07	P25676	Q57860	Q5HY92	P38844
Q58711	Q56Z59	O23461	Q1DME3	O74832	Q50336	P81912	Q02752
Q0C8J3	Q6H3X3	P76569	Q8YT42	Q86XR2	P56134	B0T0X6	A8MDI3
Q09551	P55689	Q54IN6	Q750R7	P40867	Q5ZJB4	B9L1X1	P23263
Q9WYH0	Q5JE64	Q751A7	P81058	O17816	P55472	Q96HU8	Q8ZW59
B9KHS3	Q6CQ13	P48933	P77598	P27508	P43992	Q8IVF2	P05986

Q9ZCF9	P0DH65	P86742	P39993	Q8IV32	O28688	P34380	Q9ZLU4
Q58DF9	Q8TDN4	Q2IL01	B2WKX9	Q9CX56	O34429	Q1KVU2	Q9ZCY5
Q9Y9T6	Q6ZMV9	O31969	P45267	Q9CZX2	A0N0X6	Q32L52	P34430
Q8STE8	Q47824	Q810U2	Q54W36	Q3V4R5	P21058	P97032	P0A144
Q58639	Q6PDN3	P46204	O94527	Q96NU1	P28848	B3DTV3	P0DJH2
Q97T80	Q8TAV5	Q96QF0	A3DLP6	Q9LYU9	Q8IV77	Q9STY0	P42826
A8XMF2	Q10043	Q940Q8	Q8R0K2	P81323	Q9PR82	Q00273	Q3UMG5
Q6CG20	Q99P87	Q2Y8L4	P27058	Q6NPN9	Q96MC9	P54580	Q6NUQ1
Q9JLS0	B1ZU31	P04888	Q6L6S1	A8XV40	Q5R829	Q86TU6	P19693
Q59051	Q8GUJ2	A6NEL3	D5ARG9	Q9NZV5	P08354	Q10937	Q5UP69
O95081	A1Z8E9	Q17061	Q7DLR9	Q8N7X8	Q9P8K9	P24418	P77596
O13561	Q9YDX6	P44248	P43217	P01149	P09876	Q9Z0X0	Q91145
O96923	A6ZKI3	Q53683	Q9FVX2	Q11YP3	Q54M77	Q6FNP6	P53293
Q80XI7	P10681	Q24751	Q9HV11	Q755Y8	Q54BL8	Q51700	Q9P799
P54883	C5DU06	Q1DF98	A6ZRG6	Q0P5E7	Q1RIF2	O31516	Q9ZCW4
Q02407	Q9D8U4	Q7U6V8	Q9BY27	Q8S7E1	Q5UPI4	B6KAM0	O70324
Q8GXC7	Q57640	Q0VBY7	Q10949	Q2LGZ2	Q1RMM5	P31621	Q9S597
Q810B9	Q7N4V9	Q9SCU1	Q92903	P04540	Q9NYJ7	Q9W2F2	Q24323
Q9YH58	Q91V98	Q8K4D7	Q9LJV9	A1TC01	Q17060	P54820	O83911
Q5UQ27	P55691	Q91743	Q3BTU8	Q9E6M2	O67088	P39695	Q09690
O67264	P38868	Q0II44	P80232	Q8ILI6	P74917	O95715	Q96L16
Q9USR1	P52552	D7GXG5	Q36284	Q05224	Q9ZJ48	P41734	Q3YEC7
Q6H8M7	Q5HXN6	Q58902	Q12233	O69062	A8ERK3	Q9HXY5	O43687
P80015	F5WT83	Q7P0S6	P57019	Q5K6N0	Q5B5W2	O27458	A1ST89
Q77PU6	P90895	Q86WH2	Q5UPD9	Q57965	Q6ZQK0	Q54PP6	P74002
P58832	Q8N2E2	Q7M4F4	Q24524	Q03212	P0A5G3	P55568	Q6GPH4
P22402	Q54KV6	P49269	Q7LKZ7	Q5UXB8	P11459	P18348	Q3MJ13
Q20170	P55341	Q6D913	B6VQA5	Q6GQ49	B5CY73	Q9HLZ7	Q9U6B8
Q3ZCD0	Q6FJL9	P94965	P82170	Q5DU57	Q9QJ28	Q7P1R6	P18243
P33275	O28690	Q90WJ8	Q6ZRU5	Q63ZV0	O13683	Q9XJS1	Q5UP37
O23610	Q3V0C3	Q93ZB1	Q0UNW1	O95236	P75142	O29990	Q00099
Q9KWN0	P87140	P42713	O34621	P77564	Q6NZ09	P53951	Q9PFQ9

O34906	Q01477	O82533	Q9Y7T9	O76368	P53144	Q66638	Q9NW75
Q86YT9	P81248	P0AEE1	Q77FQ3	P55585	Q0VCZ0	Q27968	Q9YGD3
P0CJ06	Q7VEP8	P28964	Q5UQH7	Q9GUC9	Q9UBS9	O64600	P19296
E7RAI2	P31414	Q31YW9	Q01969	O54820	F4IXT6	A6H754	Q9FIZ3
O81775	Q45757	Q5UQS4	Q6MGM3	P0C0X6	O84000	P46652	Q10659
Q8BG28	Q9WZ56	Q04SA4	O74426	P29337	P96769	Q07897	Q8IXL6
P42937	Q8BVA5	Q58149	O60320	Q9BVV8	P19844	Q9W6M9	Q8S2G0
O14929	P55609	Q7TSP5	C5DGI8	Q05239	P54072	Q54VG4	Q72B27
Q573G8	Q03667	Q8C494	Q58859	Q02728	Q5HI96	Q9VZE7	Q8V3U3
P25929	Q9I7X6	Q75DG4	Q9KRJ0	Q52328	Q8Y3L4	O34753	Q6C216
Q2HJ18	Q75G46	Q05491	Q5I4E6	Q03085	Q1L8H0	Q8BL99	Q55026
Q1LZD1	B7K911	Q8TB61	Q98829	P14708	Q402B2	O68943	P09485
P30326	P57260	P0A8Y3	P09729	Q06691	P33989	Q9VS59	A8FK97
Q52892	A2BM05	Q5Z6F5	P71677	D7URV0	Q0V5W8	Q00130	P83592
Q6L147	P24764	Q867X3	O87690	Q45347	Q68Y86	P04506	P96680
Q00848	O34554	Q58580	P22833	Q924C5	Q3B7S5	Q61476	E1BDF2
A8MQ11	P38986	Q03478	Q4FM64	P29068	P34622	Q5UNV8	Q99748
Q629K1	Q556K9	Q90ZB9	P47829	Q9P324	B2V8C0	P47371	P11037
Q9QZ39	Q8BH50	P14898	O29378	C0H3Y5	P58990	P46103	P20605
Q58009	Q9RTK2	P22804	P83098	Q9BT17	Q8LPI0	O19094	Q9ZK14
P37016	Q99MS7	O83921	P30813	Q9FG65	Q8QZV6	Q8NFZ6	Q83885
Q9LF38	Q38065	Q54G42	Q83AC1	P25354	P45622	O43451	Q5ZJH7
Q00224	Q8LAW2	Q9UUK4	A8FRR1	Q54XT6	P20744	O83637	O13576
P71017	Q8MMH4	D3RQU0	Q22885	O20142	C0LGH2	Q5UQI1	Q9U616
P23481	Q9U2R0	P39458	A5A8Y8	Q55G83	O13999	Q5UPN4	Q6UDH1
P20714	Q65961	Q2FYR2	Q62240	O74837	Q6ZTN6	Q8NEA9	P0DKP3
Q9D4V3	Q13QU8	O34055	Q9Z0G4	Q02889	Q9C500	P0C091	Q9LPH0
Q5UNW8	P34052	Q8R089	O94596	Q57940	Q96N35	Q9H0R3	Q50644
O27993	Q00516	P35753	P11670	O84071	Q9ZUT4	P41755	P71019
Q7ZWB7	P80361	Q8VYD6	P94691	P55717	P11469	P16480	Q04385
Q8RMH6	Q54V85	Q9UUG5	O04200	O80526	P98080	Q47866	O94330
Q9SZR7	Q2FEZ8	Q5EAE9	P39234	Q15942	Q5TBB1	P11160	Q556J6

Q58985	P59672	Q8YFD8	Q54YH4	Q969R2	Q9Y4P3	Q6PC62	Q6MTB9
B5LZ79	Q3E7Z8	P39479	Q9NDE8	Q58462	P31077	O32104	O13530
Q9I489	A8NWK4	P58224	Q28NF0	P15618	P38186	Q1MTQ0	Q9USP3
P30930	P75483	Q9LV12	P11272	Q9STS6	Q9P3U8	Q1ZXK1	E6Z0R3
Q5T2R2	O83478	Q9C7V5	P50500	Q6MQD1	Q9ZH21	P14230	Q9ZUU8
P75979	Q50422	A0JPH3	O74783	P39789	Q8G6K4	O28156	Q8DKP1
Q86SM5	Q96H72	P73930	O36414	Q8FT98	P96207	P34890	P15415
Q32PF3	Q25464	Q86Y82	O14155	P75843	Q57773	Q54B33	P54447
Q6JVE6	P55905	P0DH78	Q12110	Q99LC9	Q17745	Q5UQU1	I0CME7
Q9USD4	Q5DTI6	Q45670	Q682H0	P0CS25	Q03778	A1JKE3	O94587
O94509	Q00229	P51862	Q1MX22	P0ADQ9	P37836	Q25092	Q58794
Q96M85	Q1MTQ5	O34897	Q2QNS6	Q58463	Q91ZR4	P46322	Q9NY35
B8H9E0	Q3E7A7	O74941	Q5UR51	Q938B4	P78218	P0DKN1	P05528
Q8U410	P23110	Q5SRN2	Q12512	Q9U5P2	P65031	O83558	Q9W0M2
Q9S752	P34586	Q75JQ0	P0DJE4	P27919	Q97QZ0	P25238	O94418
A6NMU1	P14677	Q58399	Q5RC98	P25946	Q3S2Y2	O51468	Q10683
Q54C64	P40738	O28852	A1L2Y1	Q12100	Q20EX8	Q8TWL9	P95195
Q9LPC4	Q5HY64	P25562	Q76P07	Q8VC26	Q6FUS3	Q5BQU9	Q57733
Q55CQ7	P68624	Q9QJ43	Q5UQ84	Q9DCA7	Q57621	O06294	Q53VY1
P0C6B2	A5D8P8	O80800	Q84DC4	P26551	C9RPK4	P31949	Q9HAQ2
P86110	Q9SAA2	Q26626	Q09175	O28147	P29944	P75273	P03292
A4ZUB1	Q9SS86	O31702	A0ZSF6	O74363	P40417	Q8GYL3	P45844
P73545	P16395	P47380	Q84ZM7	Q8L844	Q29S11	Q98AV2	Q32KQ2
Q8ZY96	Q9BYE9	Q51ZN8	O13705	Q9ES28	P02861	Q57775	Q1QE80
Q9X448	O34855	P17624	O76612	O32047	P11922	O81908	P57752
A3DNJ9	Q5TBE3	D8R814	A5E0N1	Q8I7F8	Q9S142	P0C5R6	Q9QZM0
O74751	P14016	Q5T292	P19520	Q5VWC8	O28685	Q04174	P19401
Q9UHB6	Q02783	P44573	Q69YJ1	P36897	A4F7P5	P35811	Q6QGC0
A5DSN2	P00144	Q12168	P52625	P46374	Q24246	O08466	P82629
P40577	Q6R7H0	P50547	P0A3V4	Q86WT1	Q89DG5	Q8YN70	O61063
Q8U3V3	P0ABD2	P43167	A0T0R5	P40226	Q65Z59	P34655	P53098
P54436	C5DLM9	Q51645	Q09777	Q89PK5	O29469	P23038	Q51664

Q08722	Q96DL1	Q9VWK6	Q9PQZ3	Q24372	Q54Q89	D2C1B0	P54997
A1JLD6	Q3MJ40	P30138	P32040	P01867	O88484	Q45131	P38321
Q876Z1	Q5DW47	Q5ZMH6	P20400	P28983	Q6DAH4	Q5RAQ5	Q0WQM8
Q7ZUK0	P45289	Q03308	Q62894	Q54KF7	Q8K375	O07603	P0AEL7
Q5UPU4	P0CP11	P28629	Q52778	Q6BPL7	P41849	Q493S0	F4IQV7
Q09789	O35094	Q1XDS0	P21175	Q4P5B3	Q9USZ2	Q9ZUW3	Q8X958
P15363	Q3UST5	Q5EA46	Q9Y252	P77434	Q753H5	P73639	P29233
Q5XET4	O29171	Q99W47	P75863	Q551B5	Q96MT7	G2TRS8	Q5BKU6
P53540	O28062	P22244	Q2YBP2	P0A5D3	Q6UDF8	O01999	O67122
P0CJ99	Q8RXR2	P0CR59	Q8WV99	Q9NUT2	A1JQE6	Q9T1T6	Q08637
O88853	Q640L5	Q9W4T0	Q9XYH7	Q3E750	P0A0C2	P29939	Q58790
P87128	Q9W5U2	Q8WWN8	Q8TF21	Q2YDF7	P0CM91	O87877	Q9D9P8
O07753	B1ZUW6	Q196U9	Q59H18	P49018	P16425	D1Z4I6	P29495
P85964	Q9P376	Q1G3Y1	P20569	D4GYH4	Q2G2B2	A4FZ59	Q9ZDL0
Q06686	Q9Y6N7	Q3ZY38	P0CE10	Q6MDQ8	A4IFL2	P38758	O83978
Q2YDJ5	Q9ZQE5	Q2LCR3	P34487	Q37082	Q45710	Q9NXE4	O32271
Q9FLV9	Q99222	Q9V969	Q58775	Q37894	Q9H1L0	P73888	P0ADL8
P14872	P0CI40	P43298	P13663	Q5F5F6	Q6BMF6	P81782	A7TSA9
Q2T9L4	P16681	O77086	Q8WWM7	Q9XVX1	D3Z5T1	Q54WU6	Q9CA58
Q03070	P31789	P0C1G5	Q96KX2	Q4PF83	Q95189	Q84YK8	Q9HTK7
A6NLB4	O65888	O34745	P09915	Q6B4J5	O44514	Q6ZMK1	Q5UPU7
P27113	B1MZ79	Q9TU34	Q75C80	Q5R3I4	Q9ZCL4	P45249	A0LEQ3
Q9DAK2	P07374	A9TKY8	P71633	O67591	Q95TN1	Q12012	O17040
O14281	P46491	Q9AQS0	Q02710	P29064	Q69577	Q54WN6	P71060
Q07408	P0CI35	Q5J5C9	Q6CDX0	Q54WS5	Q9C0W2	P63505	Q44848
A2X462	P87064	Q8TAB7	Q9VZM5	Q60SZ2	P55711	Q67R57	Q9CLV6
Q9Z0H1	Q58CT0	Q58DS3	Q914K1	Q9A1B6	Q04311	Q8TJ52	Q8RVC7
A6GW90	Q5MIZ7	P85511	A7E305	Q9H825	Q3SXY8	A8MYB1	Q1WUM1
Q0QLE9	P37786	A1C3L4	Q5VYK3	Q84M47	Q1DD74	A5DZ21	Q6UDK8
Q8NTW4	O67514	Q09738	O94300	Q499T7	Q9DCZ4	Q9NQ89	O94868
Q42687	Q8LFQ6	D9XF45	Q9J558	P08175	Q8TCD5	Q5UQ03	Q5EA91
P17533	Q9YG68	O06481	P95241	Q86B11	Q9YDW0	Q5H9B9	Q07DW0

Q9VHQ2	Q1L8Y6	Q8IYA8	A5PLE7	Q870Q4	P37514	Q7XJE5	P19285
Q0VG62	P53275	Q01635	Q86PM4	Q4P5V5	Q55D51	P23044	Q8N5C7
Q8TGR3	D2TV88	O68248	P30144	Q8PWX8	Q28C55	A4ZUA3	Q5VU57
Q09240	Q4WNS8	O15482	P10039	Q21973	Q9ZVI7	P26918	Q53FE4
Q9Y3T6	Q5BCI8	Q54EW9	P34638	Q9YDM3	Q7NBF8	A6QLE7	P75919
Q9H7Y2	Q9E6M7	O58056	Q26421	O30086	P37662	P0AA90	A5EYU0
P67097	Q95LN2	P06015	Q00149	Q5HPA7	Q289L8	Q46139	Q107X0
G2TRT7	O59705	P40233	Q94CD1	Q9NS73	O06644	P46324	G2TRT9
Q08199	Q9G2W7	Q9P266	P11309	Q9V5L3	A2VE56	Q6CE38	Q3IKY9
Q9W2U9	P59046	Q9M0R6	Q6AUK5	Q9ZKP2	P38104	O64228	P20701
Q5UR23	Q6ZST2	Q6NTT6	Q86KL7	P0C5E0	P40145	Q0JA81	Q9UAS6
P0C6R5	Q9YEB2	Q07G34	Q6CFR0	Q9INI3	Q84WG0	P05945	P0AAS6
P03619	P31921	Q9R1M3	P0C348	Q49611	Q3J170	P44265	Q9LH39
B3LFC0	Q573F1	Q59014	Q4FPB7	Q1JUP4	Q65Z91	P40309	O55744
Q9KJV4	P14683	P29505	Q9FGQ6	P77147	O16868	P75292	O06983
Q58697	Q58489	Q92H16	Q14CZ0	Q6V0L0	O27945	Q6CMA4	O07565
Q5ZSQ2	P08105	O75355	P49415	O33407	B3A0P4	Q4KEN7	Q6ZNG9
P55707	O33478	Q36675	Q18473	Q2HAR0	Q12229	P22962	Q54V76
P30782	B4IL62	P10762	Q9U1M2	O13582	Q8VQB5	P18640	Q9ZD85
P57742	Q9PTT2	O31771	Q99376	Q2TBG9	Q9FMD5	P51592	P13856
Q5VZT2	Q76LL6	P52794	O69762	Q6YNQ4	Q10026	Q28516	P55049
P49767	A0RZ28	D0NVH9	Q148G8	P44052	Q08750	Q9LK65	P03762
P53081	Q54ET2	P17927	O35569	Q4ZGE1	Q8S2W4	Q9UPS8	Q6BPN7
P0A587	Q7SEQ9	Q09721	Q95PI2	O64837	Q9SH60	Q197C1	P07103
P49053	Q759K4	Q44316	Q8PLH0	O19898	Q5NTH4	P45240	Q8R5I4
O32258	Q8NEA5	O84882	Q9V9W8	Q70KF4	P29347	Q0C8Z1	P80059
Q57688	Q8W3M7	Q04977	Q5WEF9	Q08CH7	P04365	P13619	O50999
O68983	A6QPH9	Q9NPA1	Q9C112	Q6GI30	C5JHY1	P0AET9	D4AZK1
P73001	Q4J9C4	C0LGV0	P27730	O30083	P0AAR6	P15381	Q46670
Q9P7C0	A2AU72	Q9U5P7	Q2T9W7	P16548	Q8WWF5	Q58223	A6YG74
E9Q8I9	Q74LZ9	P09162	P39590	A7YW81	Q5UQ70	Q6NLC1	Q54S31
Q9XZH7	Q90233	P15610	P04109	Q07738	Q8TCJ2	Q9E2H5	P0C9R3

P53120	Q9YPD6	Q04PB3	Q08DY0	P0C8Q9	Q5W0A0	Q553Y7	Q54KQ7
Q09359	Q9ESN9	P16354	P36362	O05393	Q8EUB0	Q1IJG5	Q3EDF8
Q02197	Q6R7J3	P62557	O83425	Q2YMX6	Q7XU38	P26904	P41733
P44115	Q9BUN5	P52368	Q9Y484	P47232	Q9VLT8	Q42676	Q09423
P36807	P13313	Q12350	Q06670	O14067	B7VKH1	P0CAZ9	P26142
O34656	O34834	Q9ZD66	P47152	A7F4S1	P47876	P35874	Q10731
Q9J509	Q5UR73	Q058N4	P94404	P23779	P05630	P34483	P87355
Q9M5Q1	Q7DDQ4	P44843	Q8NBI6	O28078	Q554L2	Q8NDV1	P52609
P47485	P34130	P33300	Q9RRA9	P54452	Q7Z8R5	Q3EDH8	A7XCD9
P25371	B4MTY2	Q57583	Q5HKF0	Q75QN6	P36964	Q6GIX3	Q5DTT8
Q8N8J0	Q86QS6	A7A1V1	Q6BK07	P69327	Q8N350	Q5C9L6	Q09352
Q55DU4	Q57ZQ7	Q83HY3	P16369	Q4Z8K6	Q0VDD7	P19325	P31877
P14501	P21078	P0C7Q9	Q9PPE0	P13090	D0ZWR8	G2TRP5	Q2V493
Q9TSV8	Q40357	Q5R9N7	P01070	Q53U19	O80345	Q9NVK5	A4HUY0
P87012	Q54CI5	Q85A69	Q6ZR85	O71195	Q8WVS4	Q9ZD73	Q9Y106
Q5X4E7	Q5JGB4	Q54ST2	Q10736	Q22830	O64203	P55321	O60155
O30050	Q57867	Q10079	Q3UJD6	Q93S26	Q10918	Q8NCR6	O05512
P30907	P80387	Q9M571	Q91FD9	Q9SR43	Q8IV56	P49108	P41467
P14705	P50655	Q9DAR1	Q8QL31	Q04359	B0TQP7	Q58713	Q12523
O94111	A1A4P9	P22152	Q06199	P40985	A8MRP4	Q24212	P83049
Q0P8U6	P28255	Q8GS60	O64393	Q9L5N1	O74889	Q3SYA9	Q3ZCQ2
P25307	P90666	P21976	D3ZKF5	Q2RAR6	Q8GWZ6	Q6CVZ8	P37092
P51234	O13827	P61366	P0CB46	Q28H35	P31692	Q9LUS7	A6KYY1
A7Z439	Q89669	Q5UQF7	P59912	O14367	A2BGP7	A9P1V1	O16519
Q5UQJ5	O34352	Q57SD8	Q9WVL7	Q9LUJ0	P23689	A1JU77	Q9H379
Q5XDZ5	Q6A555	Q86VQ3	Q00140	Q09686	Q8C1R3	Q6R8G5	Q00946
Q54FP4	P75459	B8NJB2	Q1M1A0	Q696W0	P17366	Q7T2A3	O74381
O77783	P12826	O26317	Q9KS61	Q9XWV7	P41714	Q9VIF5	P35001
O05398	Q9VSH2	P75299	B3QLQ8	A5U6S3	B3LNV9	P74858	A9SNJ1
P36343	E0D877	Q9HJW8	P54678	Q2QWE3	O83251	Q9SJL0	A3LQ44
Q60994	A7XDQ9	Q54Q48	O07293	Q86VD9	P0C0K5	Q9C691	Q54HT5
Q9ZM68	Q8NE79	P34566	P0A311	Q11188	P50162	O74368	Q86KA2

P87237	P51229	Q9FF17	P96684	Q6P2K3	Q3V125	O43088	Q9SMZ4
P76004	Q73Q17	Q9LT96	P0C1T5	Q90WN7	A2TJV2	A1TL08	O90760
Q9KG52	Q3IW22	Q3E7Y9	O83564	Q9F5K6	O43075	P44262	Q09671
Q58051	P22745	Q07872	A8ALN9	Q06578	Q9CAA7	P0CF20	Q6UB35
P59960	Q96LM9	P50677	P20699	Q54WC3	O28876	Q5MKM5	Q5UQZ1
O08742	P95158	P14352	Q8ML92	Q6IED9	Q07933	Q6AXX1	Q28314
A5DR04	P36018	Q06676	P76556	P19740	Q02122	O29522	C7RCP6
O54339	P54591	Q6FEZ6	Q69YH5	Q6R7G9	Q04624	Q5FC62	O34853
Q2FQE3	Q9P7N6	Q8IYB4	P45475	O59708	Q5XA93	Q9ZT50	P77567
Q59022	Q9UMZ2	P05305	P06442	O34528	Q9CAZ0	Q59003	P47614
Q6EPW7	P46376	P71602	P19302	Q10522	P0AE28	P73879	O78459
Q9VHR8	B9WZ56	O26918	Q55BA0	Q9KPW1	Q8V9R9	O17405	P04886
P28285	O84467	P96657	Q9J560	Q4V7A5	O31814	Q978J0	Q25BI2
P36353	Q54JM9	P60063	P46504	Q08379	Q9K0V0	Q37893	P29489
Q57051	Q3SZ68	Q80Y86	Q9F5K5	Q0P5L8	Q920H1	A8MR93	D6RRG7
P29632	Q9XXV8	Q95029	B3LTC5	A8XYX3	Q56839	Q39054	A4H202
P0C9G7	P93823	O06343	P90661	Q8S8S1	A1CGA8	Q6MII5	O59740
Q02JU1	Q9ENK7	Q5RBP6	P75082	P94512	P33447	Q8MN60	Q3Z997
P14172	Q96D05	P29993	Q8IX95	Q09318	Q9H078	Q56WD3	Q9LSL9
O67720	P24240	A6VTV5	C7CM32	Q57223	Q313K2	Q91892	P55474
P21075	O78493	P04461	Q9HE05	O05391	Q01006	Q9VW71	Q8TZ52
Q24570	P0AFN8	Q9ZST8	P37496	P44621	Q9ZPR5	B1ZWP0	P83861
O32136	Q28524	Q9JMS8	P0C582	Q9R2W4	Q04949	Q86KL1	O50917
A4H1Z9							

Test dataset (TeD_2664)							
1332 DNA-binding proteins							
Q63003	P21228	Q9UBL3	Q6NKN9	Q54T16	Q14781	P56721	P11420
O30477	Q37989	O80341	Q9YHT3	B2JU32	Q6VB84	Q92258	P0C746
P15860	Q5HK76	O73932	Q24753	P12746	P09088	Q9WVG9	P52551
Q9SYH4	Q5NB82	Q8IN94	P22177	Q04049	P86252	P21538	P13606
P24928	Q9NSC2	Q24U48	O94714	Q5F4B2	Q9NAH2	A1RY32	Q9VH70
Q8GZ38	Q9SGQ0	Q9SKD9	P43011	P55610	O59780	P34437	G5EBU4
Q8WUU4	P13682	P14196	P26275	P09775	Q9M0R0	Q54G94	O34994
P46946	P64387	Q64759	P30314	P93007	P49885	Q1LMQ4	Q13283
O15499	P34470	P10178	D0VYS2	Q8C208	Q94LW4	P40971	P38304
P25846	Q700D9	Q9FGJ3	Q9M1B0	Q9FYJ2	P32896	Q7SQ98	P25502
Q8RY99	Q9X1E1	P07040	O75446	Q69GZ5	Q9BXG8	Q4P328	O73718
Q39234	Q76IQ7	Q12151	P28938	Q93WU9	Q9YFV8	P55631	O31480
O34549	Q9H171	A2CEZ5	P51505	P20945	Q765S2	Q9LVR0	Q0WNR2
Q5XJ97	P32797	P24716	P16989	Q66611	A7U6F1	Q9CA27	Q86US8
C6BQP8	P03670	C5MCK5	Q8SWA6	P31538	Q6H6Q7	Q7TP98	P03052
Q8EQQ3	Q14814	Q759V4	Q86HX9	O82248	Q24423	Q64201	Q9VK71
P16088	Q9HGP0	P35901	Q6S4P4	P13121	Q2G2B1	Q7UQV4	Q9UUD1
Q63520	P79742	Q9FX53	Q12888	Q07956	P13892	Q8VWQ4	Q58884
P55701	P77730	P43458	O43829	Q9Y3M9	P45277	B0W1V2	O95076
P78962	Q9FKQ6	Q5JLB5	Q9UXF8	P11115	Q55BA5	Q96KC8	Q25BI3
P43064	O14108	P03870	P94426	A7TQK0	Q8SVD3	P27609	Q67TP9
P05827	P08155	P40333	P0ACH9	P26359	Q54HP1	P25210	P28267
Q9X5P2	A1YF08	P03355	O97764	P41055	P22336	P69202	P41371
O14362	Q54QY7	P23393	Q95PX3	Q96EK4	Q17308	O48652	A5HBE1
Q9LZV6	Q58948	Q57949	Q8X4Z9	O59746	Q9ULJ3	Q5T0B9	P18750
P09087	P15699	P18848	Q9SFZ3	Q9FWS3	Q8LFV3	Q99089	P0AD02
P25047	O27276	O73691	A0R666	P46908	Q08605	B7ZQJ9	Q8SW51
P14150	P22813	P0CF62	Q2MHH4	Q2QW53	Q52110	Q99MT2	P04198
Q5E9X1	A8MTQ0	Q29480	P52662	P03710	Q6J9S1	B7GSG2	Q54MH9

P06153	Q38740	Q26603	Q9R9T9	O05052	Q54YG9	Q2TBI2	P37245
P52087	P95003	Q93WV4	Q2YV56	Q9FKP8	P96686	P76138	Q9Y2Y4
Q9H8N7	Q9BUG6	Q01593	Q3U0L2	Q9GPH3	Q8GT73	Q9SIV5	P47112
Q08CW8	Q6NQ88	P68350	Q6R7C9	Q7Y0W3	P10157	Q9I9K0	Q87039
Q9C882	Q5ZKW8	O64702	Q02953	Q8RXS6	Q26228	Q8S151	P22896
Q12175	Q7ZVR6	Q9H2A3	Q8IUM7	P76086	P13976	Q91WC1	O43502
Q4JYF5	P40348	Q03465	P43472	Q08143	P12954	P75433	Q9BQ70
Q8NA92	Q48514	P45612	P51721	Q32SG4	A0RCV1	Q9FGD2	P22645
Q7Z142	Q5SVQ8	Q32KV8	Q07231	P49777	Q6W4T3	P46897	Q9LQ08
Q9SK74	P23939	O94130	P35638	O80928	O59610	P13002	O00321
P29176	Q9ZB11	P15360	P15038	P52926	P42147	Q5BAZ5	Q75IP6
B5DR03	Q12041	O74502	P20428	O45521	P87558	Q9P8G9	Q97FU2
Q28147	C3P8Z9	B0S909	P22670	P78022	P09077	O05840	Q864P9
P06990	Q9SJK7	O57568	Q52873	Q83950	Q62481	Q9FL26	A0M2C1
Q057N0	O31494	O34920	Q96K62	Q6IV72	Q8NBB4	Q148M8	Q24645
Q5IS79	Q66GR3	Q10B98	P53567	Q9VWW0	Q92771	Q9M2U1	Q779J8
Q9UKW6	Q9Y603	P55317	P69781	O33149	Q02577	Q8LDF9	Q28772
A7TJI3	P10105	Q76MX4	Q9XUB2	O34829	P28698	O17573	Q60641
Q9C1A4	Q04803	O32504	P06838	Q6MAG9	D2HNW6	P66808	P52686
Q6PV68	C0SPB6	P19705	Q9M1U4	P36598	P03010	O65420	Q8L3W1
P19544	Q8GWU1	Q58038	Q83KJ8	O31943	Q64321	A1YEQ3	P0AC51
Q4FZR5	Q8I1N6	Q9GQN5	Q9ASX9	Q0JD07	Q6FUG1	A8A8H1	Q9Z1T5
Q84K52	Q9YUS3	Q10655	Q03828	Q9PSY4	Q6QPM2	Q9X3Y3	Q5TGS1
P26586	P31504	P0CF88	P18816	O05103	Q89AK2	O02786	Q9ZNU2
O17931	B8DSV1	O31178	Q4V7W5	E9PZZ1	P31244	Q0B0Z2	P49330
Q9AE24	P34228	Q5SXM2	Q9BWW4	Q49434	Q9LSD5	Q96MW7	P33786
A0QRY5	P27336	P23841	Q5V5E2	O86228	O31500	O31865	Q9P1Z0
Q8BI67	Q6NH62	P23196	Q22258	O22901	Q7XPK1	Q874J6	Q9M374
Q6FIN4	Q9LSL6	P22373	Q9LYD9	Q89AD1	Q13461	Q9LRH6	P42944
Q04832	Q00423	Q25514	P39212	P42167	Q9WU40	Q4UMJ0	O42837
O49255	P0A9G2	P38082	Q8IMA8	Q9BVI0	Q60636	Q02398	O67837
Q6CIL8	P66812	Q12MQ7	O74365	P73145	Q99335	Q9VLR5	Q17RP2

P54294	P38078	P68926	Q8R890	O68025	Q9W352	P39959	P42097
Q9R0G7	Q6ZNH5	P46106	P05052	P01551	Q5EA15	Q75K81	Q2N2K6
O69644	P37947	P40059	Q9P6L6	P39788	P40427	Q8ITI5	Q07928
Q75WM6	O49403	P27610	Q9HU78	P96629	Q8MLV1	O94160	Q5AI97
Q707Y7	Q7F2L3	Q55C24	Q08595	P28958	Q57SD4	P06601	P10862
Q55681	Q9YCB6	P17211	Q01105	O30919	Q8FLP9	P36145	Q9FMX2
P47980	P10512	P51705	P27079	B6IPE2	O53509	Q58177	O42648
P21260	P67854	Q6P0X2	Q3J6K8	P61802	Q9SSW0	Q9CAA4	Q6L5G1
P49453	Q5IFN2	P39140	Q5JET0	Q6FCX7	O86820	Q60GC1	Q9P0K8
O49741	P27204	Q657C0	P0CY11	Q9PWD6	P15314	Q96JN0	Q708A1
Q6C6V8	Q707Y0	Q9M126	Q9N4Q7	P52693	P09274	Q8ST83	P35856
P06839	Q9CL21	Q6F598	Q9UST5	A6X980	Q54X41	Q8EWT6	P41895
P0ACQ8	Q8LJT8	F4I7L1	P51894	P15482	Q73NE4	Q10528	Q6Z3U3
P54722	P45903	Q9P243	Q9D0B1	A9KTE6	P35319	Q9W0K7	P06709
Q9SD61	P0DJH6	Q46901	P31460	Q9HLK5	O82132	O54839	P06229
Q6VFT5	Q95JA5	Q92522	Q67U94	Q03372	Q1KKZ2	Q58DJ0	P08638
Q9HG13	Q6FNR0	Q07243	Q5CD17	Q09565	P54988	Q9Z7M0	Q6F6A2
P79373	P41410	P16922	Q03316	P43609	P0ACH3	P40656	Q9WZ73
P11257	P47988	Q5F410	Q6E434	P06921	P78714	Q9CG78	Q7A3C7
Q54YS0	Q1MTM9	P64588	P10754	Q5EBM4	A5VHK2	O13412	O07465
P95163	Q94C33	Q9D6K9	O32222	P06966	O94263	Q9LQZ2	P06494
F1Q514	Q8K3Q3	P91623	P40267	P0A6X3	A6NHT5	Q28598	A5F9F9
A4LBC0	Q6WRX6	Q9ZWA6	Q06455	Q6NQK2	Q10902	P20824	Q9PB63
P05066	Q84WJ2	Q4IJ84	Q9V029	P79051	P52108	Q7N125	A2TED3
P81390	P24536	Q06184	P03008	P14373	P03123	B9MN06	Q64029
Q0DXB1	Q7EZD5	P76639	Q5UR40	Q96MM3	Q9GM03	Q4L4Y3	Q5JMM1
Q32LD1	Q9SW80	Q8GVZ8	Q9UFW8	Q9KSW4	Q54XG7	O51498	P61827
P07903	A7TMJ6	Q298W7	P25555	P09590	Q14526	Q91910	P50209
Q93348	P53411	P52685	Q3U3N0	P39897	Q5E9A1	Q22127	Q9YDF6
P22995	Q51786	Q8NIZ4	O93748	P03045	O14087	A6R7F0	O24629
P35712	P13275	Q08082	Q86WV5	P37376	P34818	P28830	A0QTG3
Q9HM14	Q9ZV39	Q58335	O42953	Q09602	P17010	Q9P0T4	P05552

P46910	Q9W6D8	Q9LZM8	O65036	A9X4T1	O32192	P64315	Q9S1G2
Q24573	Q6NLD5	Q9JMQ1	Q9C009	Q9Y5B6	Q9P0M6	G5EGD2	Q5ZI72
Q6IEI0	P38144	Q68G74	P0AE71	P53035	P55323	Q8N7R0	Q94407
A1L2F3	P18493	Q8GZM7	P17888	P21867	P54884	Q9CA66	Q1HVG0
Q9LD95	P52932	O02799	Q9SR34	P09009	P05846	B2I4R3	Q80920
Q9ZX29	O43543	Q57748	Q9XFB0	Q5L299	P54992	P18733	Q5T7W0
Q9Z103	A3BH85	Q8R1H8	P39842	A3BUD2	Q12873	Q8NI51	P23792
P21189	P13320	Q9C9I2	P94548	Q726D5	Q27403	Q6DN03	P95258
Q00839	Q1KKY1	P86938	Q9HDV4	Q9LW00	O73592	A8ADG3	P0AF30
O45460	C5DKJ7	B3EWG9	Q84LH8	P74397	P55922	O33348	Q13129
Q5RE34	O34843	P23497	Q70GP4	Q75LL6	O76332	P0AAR2	Q10621
Q8BDG6	D9J034	O93257	A1SDH7	P30864	Q9USU7	P46372	A7SBN6
Q9UEG4	Q6ZN18	Q47VK9	B7ZS37	Q8L4E7	Q338N2	Q3L8U1	P49956
P87393	Q97MB3	Q00400	Q8LBQ7	Q8BHL6	A7ZIA5	P35547	P02280
Q23405	Q9P7P2	Q98SH9	Q0VBZ5	Q27355	Q8LA53	P16400	A7HAB8
Q54DA1	O02279	P28947	O57685	P76611	Q4QN02	Q9LUM4	P0CAP3
A5PJU7	P15593	P94370	B9DI20	P38699	Q9VH20	P28815	Q9YB01
P79769	P22161	O81788	P0ACI5	Q9WYG1	Q09922	O07570	O32067
O95409	O15015	B4JQ42	A2CG63	Q8GUC3	O60158	Q9LUZ4	Q7YU81
P38877	Q8LK56	B1ZN03	Q96PT4	Q9LW49	Q8NFZ0	Q05192	Q9N658
Q3SZB8	P34474	Q9F8R8	P09020	Q93WJ9	Q95QD7	Q9SNC0	Q9UMN6
Q9ZLL4	P0A3R9	Q9GTD4	P41995	O18381	Q05749	Q8WN22	P22605
Q84WP3	P89479	Q13950	P05709	P37554	O74252	Q5M948	Q8MIC2
P34184	P34739	Q196Z2	Q6X7K1	Q05335	Q8TK90	Q10096	P0A9V7
P40950	Q6FRL4	Q6AZW8	P0ACK4	Q84JT7	Q5ZM39	Q24206	Q657B3
Q9NRG0	Q09728	Q801F8	P28040	O77051	Q9SVQ0	P32028	Q8U526
Q9FN69	Q8TFF3	Q10007	A2YN17	Q1KKV1	Q07053	P0ACL7	P13658
P38564	Q73MQ8	P38830	Q9XUK7	O34777	O24463	P12540	Q9P326
P18516	Q9STF1	P19474	B9MRB2	P06701	O73790	Q6AYD2	Q16594
Q63505	O34204	P26651	P04530	Q6X7J9	Q056Q2	P0A654	P40574
P31453	O32255	Q9Y2L8	Q8N1G0	Q9AT76	Q0WNR6	P14353	Q02479
P09085	Q924A2	P10180	Q32LE6	Q19196	Q62814	Q94ID6	Q10134

Q9Z726	P47806	Q12180	Q14774	P28468	P18867	Q9Y2K7	Q9V5M6
O42954	Q8EQ23	A7NPT5	Q3MHN7	Q9BJK5	Q9NJB5	O83046	Q58NQ5
Q8BII1	Q3MCB5	Q8H2D0	Q5JMF3	C5CFS3	Q62232	Q1RML4	Q8WNV1
P72191	Q9D8P7	P16472	Q9TX44	P03632	Q9FFK0	Q8TWM7	P44680
Q10144	Q9UTN0	O34643	Q96NC0	Q3U381	Q40170	P41739	P39404
P55201	P0C6J7	Q9RC52	Q7SDY3	Q8JI10	Q4PFV5	Q9FNY0	Q3E703
Q99581	Q72TG0	Q8NBF1	P24488	P20270	Q67UX6	P71229	B0X0K1
O44952	O42709	P54794	Q9SM27	P29503	Q88FX7	Q01610	Q53PH2
P54277	P74839	P29558	P24042	P35398	Q8EWC5	B8H172	A2YGR5
P36011	Q02457	Q01664	Q9KRB2	Q9ZIB7	P03695	Q8W0F1	Q9XIK5
Q8TIN0	O13658	Q796Q6	P39592	P52739	Q8TF20	Q9Z3R4	Q8L9Y3
Q9LNJ5	O14981	Q9IG42	Q8QGQ6	Q9CWW7	Q9Z8M9	Q9Y767	Q27368
Q9LTC5	O42861	O86810	Q5NAN5	P14064	P17488	Q9XH38	P68463
B2CW77	P42584	Q24849	P13469	P81394	O95644	Q89WI8	O16810
A2ZMR9	P25425	P80001	Q9HBD1	Q67591	Q9RFJ6	A3PCR4	P52286
Q2R3Y1	O76808	A6H907	Q06339	P34534	P25980	P27426	Q93WY4
Q54UG3	Q9ZR14	O31443	O31626	P96725	P52747	Q96H86	Q5A4F3
Q9LYP5	Q700E3	P41726	P06946	Q42498	P0A9F5	C5CH91	O93746
P13055	P11308	O55208	P33244	P37582	Q04996	P41934	Q5ZM33
P28990	P52605	P96653	Q62724	P32333	Q9SPG2	O94916	P28348
Q55BR6	Q84513	P17208	Q5B8L0	Q18919	P06847	P50512	B5YKF0
P71049	Q9S758	P46676	P32086	Q24318	Q5UQE6	P05048	Q9MA75
Q9C5T4	Q6A8L0	Q04296	P52696	P39356	P42105	O95125	Q9ULD5
Q8RYD6	P30338	Q9LTS4	Q9C7S0	Q9CWL2	Q9ARI6	P0ACN9	Q5Z3Z8
O00874	P0C6Z0	Q6C764	Q9HUW0	P44780	A4FUF0	Q41558	Q22811
Q45881	Q9DGT6	Q9JJZ6	Q32MZ4	I0IUP4	Q5A1A0	Q9LXV2	Q08D88
O15226	Q86AD6	O41056	Q14863	P68588	P03040	P18629	Q9ZLC8
Q90415	Q9W734	Q01JD1	Q9C5P0	P38114	Q5XFQ6	O61660	Q7TMI3
Q06861	O22921	Q2QMT6	Q7XKC4	O94490	P39529	P37499	O75362
Q6DD87	Q9Z0E3	Q9HEV5	Q9ZPW3	Q54WN7	P32504	P41830	Q29CW0
P59966	P03261	P49882	Q9S7C9	O60129	Q9LW31	Q9Z7G4	Q557C9
Q93356	Q9US25	O94704	Q13886	Q38796	Q91573	Q54PT9	B0G0Y5

Q9SY59	P78426	P43684	A5UMB4	P23009	O69055	Q08904	P36279
Q58559	Q9NR83	E1X022	C4VB43	P32591	Q19691	Q74MZ3	Q12WQ0
A6NJT0	P03110	Q8GWF1	Q1G3Z6	P55411	O31472	P35995	Q6AHZ1
Q9NRM2	Q96IR2	Q6GLW8	Q2EGB9	Q84RD0	Q5BUB4	P25545	O23463
Q9UI36	B1AHY7	Q7Z5Q5	Q05958	B8PBQ6	P26487	P54574	Q46259
Q869W0	Q22910	Q9W539	P39343	Q9Y5W3	A4WER5	Q3YC04	A9A1T2
Q38851	Q6P4R8	Q8TAU0	Q54P62	Q973F5	Q7Z3K3	O04681	P38086
P03627	P20434	Q9Y2V3	Q6PGB8	P37956	P09547	Q9EPZ6	A2BLW2
Q9YE67	P52906	P03108	Q93WT0	Q57089	Q9ZD50	O78417	O34949
Q6P3Y5	O14978	Q6ZT77	Q6KAQ7				

1332 Non-binding proteins							
Q4FUR9	P16154	Q55DP9	Q24247	Q69ZZ9	Q7NFT5	Q1RKC5	Q54YF2
F1QGZ6	O14492	P06652	Q6DR03	Q9Y871	P55679	Q07729	P15273
Q058P4	O67776	P81922	P47049	Q8VCH5	Q38E56	Q680C0	B3QQR6
Q6CXH7	O53526	P44520	Q59570	O43067	Q55E31	Q09383	P71631
P0A8Y2	Q9WUZ7	Q52828	Q08974	Q9PQY5	A7RZS0	B0S115	P19815
Q9UW00	Q54V32	P86223	O15453	Q2V4I2	O00626	P85064	O30315
D9U2B0	P12256	O31784	Q559A2	Q5RJH6	Q9UI26	P32383	Q4P683
P52384	Q874L7	P25147	P25306	P42359	Q3MIP1	Q8TX79	B0G140
Q8C5P5	Q9CAR3	Q0WU02	P36630	O59635	Q9FEB5	Q06282	Q39980
Q6NPT8	Q1IHD2	Q89FG4	O43125	Q07560	O74490	Q9Z869	Q8N7B6
P0C0I7	P59987	Q9D3J8	P40080	Q8IXL7	P0CZ10	Q9UUN5	Q57889
Q5UQZ2	Q54B20	Q05296	Q9GQ63	O67257	Q9Y5J6	Q9XGX9	P0AD11
Q9USW6	P39495	Q9Z8J7	Q6GZU9	Q8IW35	A1A4S6	Q9V6K3	P42593
Q26643	Q6CIF3	Q11124	Q04080	P09296	O74867	Q6CPK7	Q59UG3
P50168	P24651	Q57857	Q9V576	Q9FGL0	Q61190	Q04212	P34366
O14242	Q8N614	P54661	A5F660	Q56902	Q5UNY7	P14503	O15537
Q5SNQ9	P15633	Q68G75	Q08815	Q9Y442	Q84TF4	P34645	P10018
Q2YAV2	O66410	P53225	P17983	P16805	P0C8P8	P0AAE1	Q10212
Q9IBG7	O88492	Q00143	P0CD62	Q07895	Q7NFT6	Q6DIV5	Q2NL17
Q5L586	Q6CRV9	Q76PC4	Q8KCC4	Q83HJ9	P30651	Q9LM63	Q08317

Q619N4	Q9I1M2	Q9UZC1	P46086	Q8D2U9	Q6PC40	Q64281	Q9WXE6
Q65NX1	P11435	Q8VPF3	Q64537	Q32L08	Q17R16	Q5GC94	Q8R3J5
Q9X016	Q8WMH2	P0C6Z1	O28254	P0AF00	P23208	Q54XC4	Q9BM90
Q60AU6	Q58680	P93290	Q2KIQ8	Q9XJS6	O28642	C0H3Z7	A4QI94
P53616	Q8U1R0	Q54XA2	P39059	P46023	Q9LY32	Q3TCV3	O73683
Q04322	Q9Z2C4	Q8PXD4	F4J117	Q5UP41	Q9M190	Q54L83	P12611
P37197	Q4FUR4	Q5VQY3	Q08467	O95727	Q8TYR8	O05156	Q9UHP6
O83811	Q9RND2	O82368	Q90665	P24665	P50524	Q7X9V3	Q47AR4
O64243	P42719	Q8JFZ2	Q54I54	P44193	Q3KPS4	P64741	Q03605
O31790	Q9AST6	Q00189	P56544	O26618	D3YUG0	P44465	Q8LFL4
P80565	Q3E7A5	P41820	Q3JV52	Q7TNJ2	Q3UFQ8	P41885	Q0WVL7
Q9VPW1	Q6NUN7	Q22949	P85500	Q08CL8	Q6NUJ5	Q63135	P91276
Q60401	P80354	Q9WZS2	Q9QZK7	O13340	A0JPP4	P35826	A4QDF8
P49583	P25358	A8MGE1	Q9YAA0	O06652	Q8N144	P25143	Q9UUH2
P73555	P09126	O47558	P91851	Q2TAM9	P86729	E5KIB9	Q8UC57
Q6GLM5	P80519	Q6VAK4	Q6ZVL8	Q5UP07	Q55FM5	A8MXB1	P81311
O32066	O18495	P95262	O66477	O07899	O31723	O14157	Q0IQN5
Q15032	P32454	Q54RE1	Q5U5R9	Q6C315	P10505	Q6FJW2	O44783
Q8MYY6	P90970	P50466	O52908	Q9H9V9	Q10138	F5HFA5	Q9W2B1
Q9CF93	O31710	Q9K499	Q2YJ76	Q0VCR6	P77515	P64829	Q86282
P35182	D4GQ17	P15328	Q54JC2	A0SIF1	P53224	Q7WR85	Q6PBD1
Q0IIX4	Q54QQ6	F5HGQ8	Q5R7Q6	Q97VM6	Q9H8Q6	P00217	Q83I01
P86247	Q32L75	P0DJ93	P0C5M2	P54494	Q9I3N4	P00782	P50725
Q92576	F4I8B9	Q8BPQ7	Q7KT91	Q02875	A0JMD4	Q7SX85	C0LGH8
Q13QI0	P63186	P03975	Q7YQK3	Q9C5J4	Q5EA84	O34474	O76878
Q0V7M1	Q18262	Q54I86	Q92348	Q54MY4	Q5BK32	O94283	Q9ZVJ6
P19802	Q3KPI0	Q09369	Q96G27	Q2KIB3	Q06171	Q5SPV6	Q50834
Q1ECF1	Q5UP27	Q5FF65	Q9DA64	P21063	P0AAR3	Q1AYN1	O59669
O74416	P81730	Q00305	P40147	P48336	Q2V3Q8	Q8WVI0	Q57800
Q55DA6	A6WYX0	Q68DN1	Q6TS43	Q56307	Q9Z138	Q61072	Q47VK1
O94830	Q14CZ7	P17571	Q6K762	Q79FZ4	Q51693	Q9FZD4	Q8X6R3
Q3SWT5	Q0IIC2	Q54NU5	Q54J33	P15443	P32804	P35538	P25344

Q55DV8	Q6PK18	P55190	Q54LT8	Q8TPU8	Q9LW55	Q9I106	P43993
P0A3U6	Q9ZN54	P20633	Q5R0C0	P15262	O62771	Q5I148	Q9SRD8
Q1ZXB9	Q17QE0	P61471	Q8R404	P21324	P44082	P19731	P06424
P0AAQ1	P07503	P77368	A7Z4X7	Q968X7	Q2QAV0	Q7ZXF1	A0R5G1
Q9LUJ2	Q03042	Q9XWN6	Q5H7C3	B3DPJ1	Q8R5M4	Q9NWM0	Q6DCC6
Q8NSL3	Q7Z1V1	A6QLD5	Q5UQR5	A2RAY7	Q9KJE8	O34929	O16785
P67098	A7TP86	P25619	Q12182	A9M186	O62246	Q54IV8	P64785
P20334	P17418	Q196Z8	Q7XSZ4	P32024	P44187	Q3V4V8	P0AB36
P44203	Q9R168	P40083	B5XG43	Q9T068	Q9C0W4	P49329	O32132
Q5MJP5	P0A243	P0CAY5	B8GYW7	Q86YZ3	P45103	Q2XVR7	Q86286
Q75B79	P0C550	P45182	Q54KW6	P0CB21	D9SM34	Q7XJM2	Q84NC9
Q75AS3	P09870	Q9WZ43	Q55587	P34390	Q8MIT6	Q84WW1	Q9BRQ0
Q55368	A1SVX3	P49071	P38066	Q5UQP2	Q05957	O28550	Q9UIY3
Q9IAX2	Q10322	B3LM44	Q58766	Q9X0Y7	O67171	P45023	Q9HPL2
P28893	P21760	P08930	O35013	D1FNK4	Q6A5A3	P18006	P00136
Q9T1T2	P30345	C0H3W7	Q5PH63	P55527	P19048	P0C2W8	P81726
Q2WGJ9	P75296	Q96S38	B8MR69	Q09815	Q9Y5S1	A0T0H4	Q7TMS5
Q20035	Q10338	Q9YGC1	Q567G6	Q2FDU6	P37536	Q18LD4	P21890
Q5UR07	Q07510	Q9YBL2	C5DKA0	P08452	Q12476	Q942P9	Q9GZN4
Q9K9E6	P79400	P77414	P42910	Q8LAD0	Q96MU5	P37663	P19273
Q86I14	P13249	Q2NL13	Q914J6	Q8AXS4	P86837	Q9BXW4	Q51795
P0C5E1	Q914K4	Q12225	Q55842	P59646	O05755	H3BU77	C0H400
P25184	P43790	Q9C093	Q5B993	Q1LXZ7	Q09822	Q13099	Q54MV6
A4Q9F3	Q2M243	P34256	Q9US48	Q96S66	P19517	P58368	Q9NQZ2
Q9LQQ1	Q9CIT4	Q9LT79	Q8SQZ4	Q9NP31	P48510	P96853	E9AIE8
P42071	Q7VHY7	P54004	Q833Y3	Q07275	Q3JEM4	O07533	Q9SP35
O24915	Q9T4W8	P63753	Q9P7K1	Q6GMV1	O73707	Q57457	O67076
P75594	P05462	P92563	O13548	Q2FK96	P55759	Q58518	P25965
P31279	A6MWS7	P15156	A1VZY1	Q8YA32	A3EXG6	Q09147	O13776
Q5TU55	Q496Y0	P03322	Q9W5A5	Q8SR40	Q9V2X0	Q8IWA6	Q98ST5
O23491	Q96A99	Q0I442	Q9X0H9	Q6DFW0	P15698	Q9NX36	P0C8Q0
O34478	Q9UUN9	P14491	Q6ZUC2	Q9REB3	P54513	Q18LE4	Q830R7

P45067	P89036	Q9ZD54	P54607	P77174	Q600K7	B3PY58	P58073
Q9HKK0	Q56206	P33968	P74897	Q9ZB81	P50658	P41424	P48928
P55794	P35722	Q8WSV0	P39088	Q9XUB9	Q9M4E3	Q7M732	Q8JGS1
Q8CHD8	Q7DDB6	Q9SWG3	Q0AC83	P23469	Q9QZS3	A2VD23	Q8RWD6
Q9SZT9	Q1LZI1	P28817	Q9LVS3	Q936X3	Q6FL09	Q70D51	Q4PI64
P31391	P22623	Q8GXI9	Q9P7S8	Q9BXJ3	P87236	P28945	Q8VI88
P11900	Q9QJ13	Q9N598	Q7NB93	P43685	Q6MPS6	Q1PEU9	P05044
A4D0T7	Q54NW0	Q54DU9	Q54T60	Q54JV8	Q3SZ13	P71038	Q8SW19
O59764	O28167	P0C2D7	Q54L43	P10307	P39426	P39709	Q87CA2
P53705	Q86SQ0	Q9NTJ4	P32917	O64584	P52519	Q6GVH4	P32382
Q8INB9	Q13651	B3LJR0	E5EZX0	P0AEQ0	P21978	Q9PJN1	Q8WWN9
Q86XS8	C4Z0F4	Q10151	Q5UPA5	P76015	P53731	D4GPW1	O83848
P0A9I2	Q58283	Q0P5A4	Q31696	Q8N1D0	Q95NH6	Q1CAP8	Q8GXN6
Q495Y7	Q9K794	P02516	Q9M0Z6	Q12157	P42298	P13432	Q58166
P0A3P0	O31877	P55885	P73634	A4ZUD4	Q56K04	P68628	D2Y3T7
P21303	Q8ERW3	Q85FG7	P0CM75	O08684	Q9V3R1	Q9FZ57	A0BKG2
Q7VMF9	Q86X29	Q9FND8	Q86VW1	P97531	O80436	Q09623	Q6Z4A7
Q02891	Q96329	Q57493	Q5M9Q1	O12000	O13375	P06579	Q9D9W0
O66891	O64471	Q5E9Y2	Q75CF5	O15194	Q5UQS3	Q05019	P44570
Q9USP6	Q8C1T8	P0ADJ1	P53949	Q11118	O83451	Q1X6Y5	P04459
P25601	A8QW39	P96647	P23935	O30138	Q63751	P00168	Q5RAW7
P49801	Q86H87	Q9VA27	Q5JF37	A0MSJ1	P43597	P19061	O81643
Q6GLQ4	Q54MH0	C4A0D9	P76393	Q58458	Q8TEU8	P33941	A7U6F0
Q5UNU5	O69729	Q4P5F5	P38349	A4FUZ6	Q15RS7	C5E2E7	Q66HT8
Q76NW7	Q9SYB1	Q6DG60	P58030	Q8CF25	Q197E3	Q54Z85	Q96WS1
P54549	P46016	Q9BEG9	P31925	Q5XIX8	Q9FFR6	Q91ZE9	Q9KWH3
O22891	Q0A8V6	Q3J164	Q57659	P0A1L2	Q58199	Q5UP96	P09031
P41908	P64457	G2TRR6	A4IFH6	Q8REI1	Q5HJ91	P35992	Q6R2V6
Q9SHI1	P32509	Q13164	Q8BH55	P94017	Q9FI80	Q21624	P38482
P29683	Q8R8R6	P55570	B3DZT0	Q70Z44	Q1DKE6	A7ZPJ9	Q9UT27
Q8T293	Q4EW11	O10278	C5FF22	Q8VII0	Q44773	P0AFR0	P09038
Q4R8C7	Q92361	Q9VMW8	A8MUP2	Q4WNB5	Q9HFF2	Q60369	Q6MSE7

A5DE24	Q9WWW4	A5PN52	Q02586	O33603	P38505	Q9Z0C9	Q8YCQ8
Q8SUM8	P12352	P29907	A2VE22	P81712	P28617	P25947	Q97J00
Q9Y6R4	E7F9T0	Q09573	P54232	Q70EL2	Q8QHJ9	A8XSW2	Q61ZW5
Q08299	P52716	Q8N4S0	C1JJY3	Q9PR97	P34800	Q86A68	Q99KN1
P82982	P04519	Q2NF85	Q9BZ68	Q5V061	Q8N0X4	Q05032	Q96LJ7
Q9CM13	Q54NF1	Q54367	P08353	P31808	Q9CPD1	P0A5Q5	Q04VC9
Q18KN9	Q60453	Q58330	Q5UQP6	Q9SN51	Q86WC6	P34289	P15896
P87268	O76198	P43939	Q54UW8	P30659	Q6R7K7	O29177	O83340
Q8NQ93	P86862	P23678	Q9ULD4	Q54TA5	Q6FIU0	P54371	P36148
Q6NUQ4	Q58313	Q6UDF9	P04023	P0AEC9	Q2FUW3	Q03900	Q196V4
Q9VNB3	Q9QJ46	Q6JBY9	O74916	P09789	P47558	Q9ZE28	Q0DGG8
Q39108	P10297	Q616J1	P37694	Q73G32	Q8XBJ4	P48246	Q6I5B2
Q58382	Q87QY9	P40866	P30170	Q0WTI8	B8D8G1	P76510	P53162
Q74N24	Q9I644	Q5UR80	P53105	P19307	Q8NMM9	Q4UV74	O32278
E5KIB6	C5PEI9	Q2PC93	Q22366	P36168	P28876	Q71F54	Q4W9B8
Q9R0E1	Q9LSB8	Q9KIG4	H2KZB2	Q9LHI7	Q9P903	P21840	Q6LYC4
Q8G5R2	A5PJI7	P53124	Q9SMU4	P55086	O07737	Q7XT08	Q54CS7
Q9LQW4	P54857	Q06703	O34574	P29370	P26093	P51861	Q555E8
P0AFY4	Q9FNH4	P47352	O34452	Q1DB04	Q54FK2	P0A0Q8	P09821
Q9BVM4	Q58727	B3RHU5	Q0D2G3	P0C8E7	P46521	D9U2A3	A9JR56
P80427	P44021	Q9PLN5	Q9JLC8	A6R8Q8	O94812	O84462	Q4A696
Q965Q8	O74990	Q75A03	Q92275	P22317	Q6AGI5	Q49XT4	Q54C18
P31845	Q08271	Q94C25	O87875	B1Q006	P13258	Q96WW2	P38180
Q5UP42	O00398	Q9LRB7	Q9N126	Q9RL35	B0S8I0	Q8KA73	Q88BY5
A7AWN0	Q9C5D0	P39379	Q62147	Q59295	Q70LD0	Q06536	B3LPE4
P02588	P50824	P25956	P49781	P54587	O54885	Q8QL49	Q09953
P42634	Q58393	Q23688	Q07946	Q54BW4	P34244	Q7TT23	Q54BN8
Q9SX85	Q95QC4	P38213	Q8WVZ9	Q0WQW5	P43865	Q8MML5	Q2UP89
Q8ZWQ7	P40260	O06151	Q6BW41	P55496	C3MZF0	O42858	B5X216
F2M971	B0SCK5	Q5UPK1	Q97PN8	Q21DK2	P75042	Q5M0S7	Q9D6Y1
P76044	Q00017	Q1DNB0	Q54BC2	O31960	O10354	P22468	Q3K4T4
Q12036	Q196X8	Q94519	P20094	Q5UPH6	P37097	P0AD55	P15611

P81164	Q3E747	P0DKM1	Q4UJJ2	Q44585	Q86AC8	Q8R4P9	Q2M389
Q7XA40	B9DFX7	P21520	Q54BH5	Q9P896	Q9Y1J3	Q9NSC7	P0C6E7
Q09825	P0AE17	P43047	Q9XYF4	O06557	P72835	P48635	P74193
P29285	Q54HW8	Q8BGT0	Q8TC99	A9M3N5	Q54UT2	Q5V6B8	Q86IZ4
P77599	Q09921	Q197D4	A7HJZ9	Q96CD2	Q97CR4	P51547	P34282
A1AU70	P93300	P0CAN9	Q65178	A8FHW9	G2TRM0	O28717	Q06094
P14613	P0A9V0	Q55E72	P38939	Q9NSY1	B4QIS3	Q5H071	Q10003
P50872	Q8NE28	Q8K0L0	Q8FDI4	Q10633	Q9RC53	Q9X2V9	Q3E911
Q2FFY7	Q5VXM1	P73456	Q944H5	P78423	Q9SA41	Q5JXM2	P23516
Q76PC3	Q54VI5	Q8RXD3	Q8STJ2	Q9NPL8	P94560	Q9HLU2	Q04566
P40683	Q8C708	P25614	O08249	Q12064	Q5UPP9	Q035X6	O52749
P04533	P37227	Q9ULZ0	O31909	H2A0P1	Q3V4W9	Q6C4Y4	Q07383
Q3E7A3	Q9T0H2	Q04QD0	O61363	A0JNW5	O74755	Q0E3C8	Q8TF61
Q05682	P17119	P13463	Q14DL3	Q9H2C0	Q28198	P51871	O48956
A2CEH0	Q969A8	Q58275	Q99J72	P50837	Q9C6J3	O68873	Q46840
Q32L59	Q9P7G4	Q652K6	P0A5Q7	Q5N8G1	Q9FL54	Q8V7G4	Q9BXT2
P86176	Q60292	Q9LJQ5	Q00045	Q5UPX2	A0RTV6	P24372	P43984
Q65389	Q6B8Q1	P64905	O34880	P01087	Q08498	Q02598	P18033
P20550	P33957	P64477	O94682				
